# The First Insight Into the Supramolecular System of *D,L*-α-Difluoromethylornithine: A New Antiviral Perspective

**DOI:** 10.3389/fchem.2021.679776

**Published:** 2021-05-13

**Authors:** Joanna Bojarska, Roger New, Paweł Borowiecki, Milan Remko, Martin Breza, Izabela D. Madura, Andrzej Fruziński, Anna Pietrzak, Wojciech M. Wolf

**Affiliations:** ^1^ Chemistry Department, Institute of Ecological and Inorganic Chemistry, Technical University of Lodz, Lodz, Poland; ^2^ Faculty of Science & Technology, Middlesex University, London, United Kingdom; ^3^ Faculty of Chemistry, Department of Drugs Technology and Biotechnology, Laboratory of Biocatalysis and Biotransformation, Warsaw University of Technology, Warsaw, Poland; ^4^ Remedika, Bratislava, Slovakia; ^5^ Department of Physical Chemistry, Slovak Technical University, Bratislava, Slovakia; ^6^ Faculty of Chemistry, Warsaw University of Technology, Warsaw, Poland

**Keywords:** difluoromethylornithine, DFMO, eflornithine, ornidyl, coronavirus, SARS-CoV-2, fluorine theranostics

## Abstract

Targeting the polyamine biosynthetic pathway by inhibiting ornithine decarboxylase (ODC) is a powerful approach in the fight against diverse viruses, including SARS-CoV-2. Difluoromethylornithine (DFMO, eflornithine) is the best-known inhibitor of ODC and a broad-spectrum, unique therapeutical agent. Nevertheless, its pharmacokinetic profile is not perfect, especially when large doses are required in antiviral treatment. This article presents a holistic study focusing on the molecular and supramolecular structure of DFMO and the design of its analogues toward the development of safer and more effective formulations. In this context, we provide the first deep insight into the supramolecular system of DFMO supplemented by a comprehensive, qualitative and quantitative survey of non-covalent interactions *via* Hirshfeld surface, molecular electrostatic potential, enrichment ratio and energy frameworks analysis visualizing 3-D topology of interactions in order to understand the differences in the cooperativity of interactions involved in the formation of either basic or large synthons (Long-range Synthon Aufbau Modules, LSAM) at the subsequent levels of well-organized supramolecular self-assembly, in comparison with the ornithine structure. In the light of the drug discovery, supramolecular studies of amino acids, essential constituents of proteins, are of prime importance. In brief, the same amino-carboxy synthons are observed in the bio-system containing DFMO. DFT calculations revealed that the biological environment changes the molecular structure of DFMO only slightly. The ADMET profile of structural modifications of DFMO and optimization of its analogue as a new promising drug *via* molecular docking are discussed in detail.

## Introduction

In the current pandemic era, the discovery of highly effective and safe antiviral agents is an urgent priority. However, the development of new drugs takes more than ten years and is very expensive (billions of US $). What is more, only one-in-ten potential preclinical candidates (from hundreds of others) can be approved by FDA. Many promising agents are rejected in clinical trials due to toxicity. Therefore, known drugs are increasingly repurposed toward new indications of treatment. It is a time-efficient, cost-effective and safe approach (Li et al., 2019). One of the well-known, still investigated drug is *D,L*-alpha-difluoromethylornithine (DFMO), also called eflornithine or ornidyl. It has been known since the 1980s (Meyskens and Gerner, 1999; LoGiudice et al., 2018). Firstly, it was approved by FDA in the treatment of human African trypanosomiasis (sleeping sickness) (ornidyl1, United States) (Pepin et al., 1987), as a hair growth retardant in female facial hisrutism (Vaniqa; Allergan, Irvine, CA, United States) (U.S. National Library of Medicine, 2021; Wolf et al., 2007). Notably, DFMO has a significant and promising inhibitory effect on diverse cancers, such as leukemia (Bacchi et al., 1980; Xie et al., 2008; Casero and Woster, 2009; Alhosin et al., 2020), skin cancer (Elmets and Athar, 2010), breast cancer (Xu et al., 2008), prostate cancer (Arisan et al., 2014) and pancreatic cancer (Mohammed et al., 2014), cervical, small-cell lung cancer and melanoma (Levin and Ellingson, 2018; Levin et al., 2018), gastric cancer (Aziz et al., 2020), colorectal cancer (Gutierrrez et al., 2019), neuroblastoma (Lewis et al., 2020), glial tumors, such as malignant gliomas (Levin et al., 2018), either as a single or in combination therapy (Wallace and Fraser, 2004). DFMO has anabolic, wound-healing and immuno-enhancing actions. It improves the functioning of the liver and is helpful in the detoxification of harmful substances (El-Saber Batiha et al., 2020). DFMO has enormous potential as a broad-spectrum drug in the fight against diverse both RNA and DNA viruses, such as dengue virus, zika, chikungunya virus (Mounce et al., 2016), hepatitis B virus (Mao et al., 2020), human cytomegalovirus (Gibson et al., 1984), herpes simplex virus (Gibson et al., 1984; Pohjanpelto et al., 1988), coxsackievirus B3, ebola, hepatitis C virus, sindbis virus, Japanese encephalitis virus, yellow fever virus, enterovirus 71, polio, rift valley fever virus, vesicular stomatitis virus, rabies virus, la crosse virus, semliki forst virus, as well as Middle East respiratory syndrome coronavirus (MERS-CoV) (Mounce et al., 2016a; Huang et al., 2020). DFMO significantly reduces viral action (Mounce et al., 2017) and potentially decreases MERS-CoV replication (Mounce et al., 2016a). The binding sites of DFMO on the surface of Covid-19 proteins are considered as well (Wallace and Fraser, 2004; Firpo et al., 2020; Perišić, 2020).

DFMO is a fluoro-based analogue of ornithine, a basic amino acid in the body, useful in the urea cycle as it eliminates excess of nitrogen. It is an irreversible suicide inhibitor of ornithine decarboxylase (ODC), the primary enzyme in the biosynthesis pathway of polyamines, which being metabolites ubiquitous in eukaryotic (and prokaryotic) life, are essential for the cell’s survival. These molecules bind DNA and/or RNA, and thus regulate many essential cellular processes (i.e., transcription, translation, cell cycle, chromatin remodeling and autophagy). Ornithine decarboxylase 1 (EC 4.1.1.17) (ODC1) is a key enzyme that modulates the initial step of the polyamine biosynthesis pathway by catalyzing the conversion of ornithine into putrescine, which is then transformed into spermidine and next into spermine. The homeostatic balance of polyamines is often disrupted in diseases. Hence, inhibition of polyamine metabolism offers a huge opportunity to develop therapeutic paradigms (Miller-Fleming et al., 2015). Firstly, viruses may have similar mechanisms in the context of polyamines (Huang et al., 2020). The investigation of the role of polyamines in regulating viral infections began ca. 50 years ago (Gibson and Roizman. 1971) and has continuously progressed. Only very recently it has been reported that polyamines are involved in the viral life cycle of viruses to promote transcription, translation and viral packaging. Viruses often manipulate the polyamine pathway (Mounce et al., 2016a; Huang et al., 2020). The recent scientific findings have revealed that inhibition of biotransformation (metabolism) of polyamines by DFMO limits SARS-CoV-2 replication (Firpo et al., 2020). Thus, polyamines can be of great value in terms of development of modern antiviral drugs either in current or future virus outbreaks. Secondly, the polyamine pathway as a target for anticancer therapy has also been explored. In this context, DFMO is relevant as well. As a side note, it can be mentioned that the cooperation of DFMO with natural polyphenol curcumin (Jennings and Parks, 2020; Manoharan et al., 2020) significantly increases the inhibition of ODC activity (Murray-Stewart et al., 2018).

**FIGURE 1:**
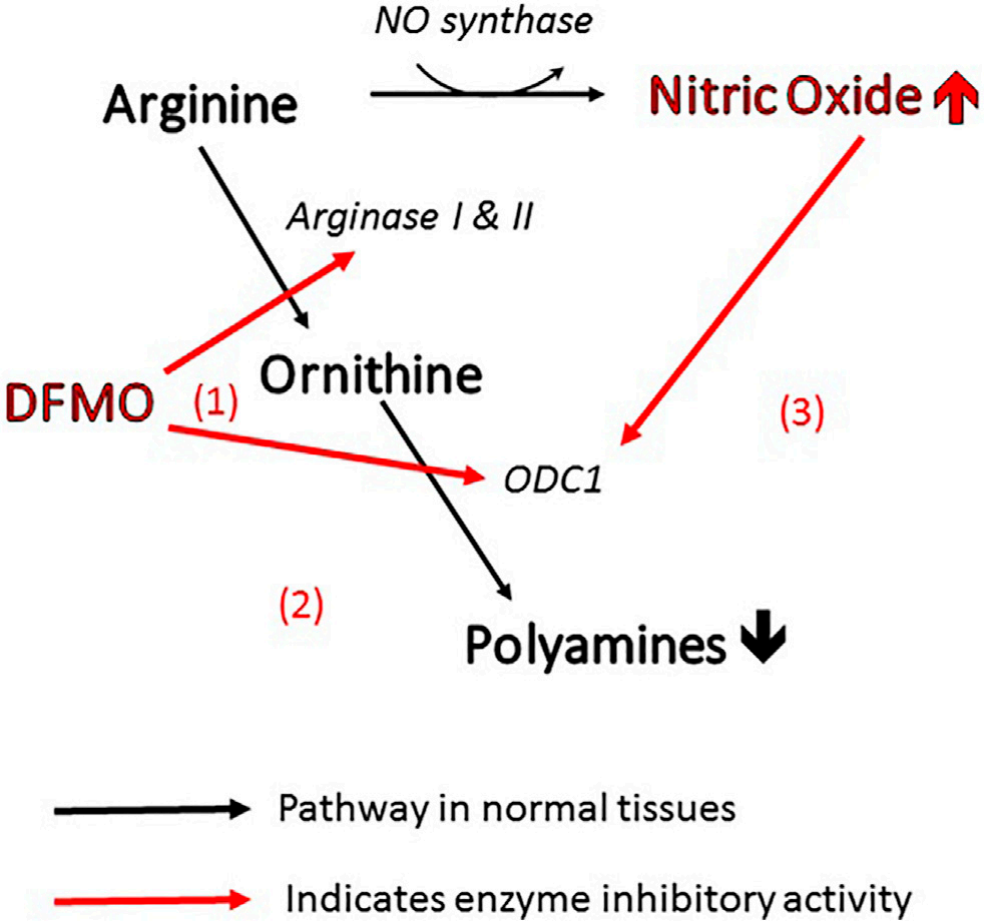
Three ways in which DFMO acts to reduce polyamines and increase NO. All of these changes inhibit viral infectivity.

More specifically, the efficacy of DFMO in altering polyamine levels is attributable to the fact that it can act on the polyamine pathway in three different ways (Figure 1). As mentioned above, it is the archetypal inhibitor of ornithine decarboxylase (site 1 in the figure), inhibiting the conversion of ornithine directly to putrescine. At the same time, it can act on arginase (site 2) to prevent the biosynthesis of ornithine from arginine, thus depleting the pool of ornithine available for conversion to polyamines (Li et al., 2002). Finally, inhibition of arginase increases the concentration of arginine in the amino acid pool and causes an increase in nitric oxide *via* NO synthase (Zhang et al., 2001). Nitric oxide is also an inhibitor of ornithine decarboxylase (Bauer et al., 2001), so the inhibitory action of DFMO is further reinforced (site 3). This trio of complementary influences results in a very powerful reduction in polyamine concentration.

In view of the above, a wide therapeutic range of DFMO shed light on a new avenue for targeting the polyamine pathway for the development of other innovative pharmacologically active agents (Mounce et al., 2016a; Mounce et al., 2017). The antiviral and anticancer studies can have go hand in hand with the advancement of the novel co-target therapy.

Another interesting issue is that fluorine-based biologically active substances are of great importance in the fight against viruses due to their specific features of fluorine. This small (van der Waals radius ∼1.47 Ǻ), highly electronegative (3.98 Pauling scale) and reactive atom has greater liphophilicity than hydrogen, while the C-F bond is stronger and more stable than C-H (Shah and Westwell, 2007). Difluoromethylene moiety provides additional profits in relation to conformation or physico-chemical features (Shah and Westwell, 2007). The F atom, by changing H-bonding interactions, can reduce oxidative metabolic clearance (Meanwell, 2011) and stop the generation of undesired metabolites (Cavaliere et al., 2017). It is noteworthy that the fluorine substituent in amino acids can modify their key properties, such as hydrophobicity, polarity, or secondary structure propensity, and consequently optimize protein folding, protein-protein interactions or enhance ligand-protein interactions (Berger et al., 2017).

Fluorine-based agents have better pharmacological and biological sequelae (Wang et al., 2014; Al.-Harthy et al., 2020). They significantly increase biological activity*,* aqueous solubility, dipole moment, pKa (Hagmann, 2008; Klebe, 2013; Pollock et al., 2015), enhance membrane permeability, metabolic stability, biological half-life, bio-absorption, safety (Al.-Harthy et al., 2000; Barnes-Seeman et al., 2014; Nenajdenko, 2014; Gillis et al., 2015; Westwell, 2015), biological potency (Ferreira de Freitas and Schapira, 2017), and binding affinity to target proteins (Al.-Harthy et al., 2000; Kim et al., 2000; Kirk, 2006; Tressaud and Haufe, 2008; Zhou et al., 2009; Persch et al., 2015).

DFMO administration is also safe. It has relatively good bioavailability and tolerability, low toxicity, mild and reversible side effects (gastrointestinal toxicity or myelotoxicity, a decline in sensor neural hearing). It is available in several forms (oral, topical, intravenous). However, large doses are required in antiviral (or anticancer) treatment, which leads to the deterioration of the pharmacokinetic profile. The *L*-enantiomer of DFMO is 20-times more likely to form an enzyme–inhibitor complex than the *D*-form (Qu et al., 2003), but the *D*-form is suggested as less toxic (Levenson and Shaked, 2003). In order to overcome limitations, structural modifications and/or a combination therapy with other drugs is recommended to achieve optimal treatment outcomes (Creaven et al., 1993; Alexiou et al., 2017; Li et al., 2019; Alhosin et al., 2020).

This work is a continuation of our studies focusing on the paramount goal of supramolecular exploration of biologically active systems, especially short peptides and modified amino acids (Główka, et al., 2004; Główka, et al., 2005; Główka, et al., 2007; Olczak, et al., 2007; Olczak, et al., 2010; Remko, et al., 2011; Bojarska et al., 2012a; Bojarska et al., 2012b; Remko et al., 2013; Bojarska et al., 2013a; Bojarska et al., 2013b; Bojarska et al., 2013c; Bojarska et al., 2014; Bojarska et al., 2015; Bojarska and Maniukiewicz, 2015; Remko et al., 2015; Bojarska et al., 2016; Bojarska et al., 2018a; Bojarska et al., 2018b; Bojarska et al., 2019a; Bojarska et al., 2019b; Bojarska et al., 2019c; Bojarska et al., 2019d; Bojarska et al., 2019e; Bojarska et al., 2020a; Bojarska et al., 2020b; Bojarska et al., 2020c; Bojarska et al., 2020d). The article is the first one, according to our knowledge, to gain deep supramolecular insight into the very popular drug DFMO (**1**) (Supplementary Figure S1), an ornithine derivative (Bojarska et al., 2020b), which is still being developed as a promising broad-spectrum therapeutic agent. It is surprising that its crystal structure has not been determined so far, although the compound has been known for almost half a century. As DFMO is a fascinating antiviral therapeutic, the extended experimental and *in silico* studies are presented from both basic and applied viewpoints. One more important point is a thorough survey of supramolecular interactions forming diverse types of H-bond synthons at the subsequent levels of hierarchically organized architecture. Hirshfeld surface analysis, enrichment ratios, electrostatic potential and energy frameworks were carried out to explore the differences and similarities in the interplay/cooperativity of interactions in comparison with ornithine (**2**), CSD code: ORNHCL12, namely *L*-ornithine hydrochloride (Supplementary Figure S1) (Dittrich et al., 2007). Interestingly, catena-[(5-ammonio-2-(difluoromethyl) norvalinato)-(m-chloro)-dichloro-copper monohydrate is the only known crystal structure of derivative of DFMO deposited in the CSD as IHEPES (Obaleye et al., 2014). Nevertheless, due to its structural and methodological discontinuities, this substance was excluded from analysis. The role of amino acids in biological processes cannot be mimicked by other compounds (Groom and Cole, 2017; Apostolopoulos et al., 2021). Another crucial aspect of this work focuses on the therapeutical improvements of DFMO *via* its structural modifications and optimization. Rationale for modifications of DFMO using additional fluorine substitutions are described in the section on ADMET below*.* The design of novel, potential fluorine-containing, more effective and safer antiviral therapeutics is discussed. In the light of these issues, DFT, molecular docking and *in silico* pharmacokinetic studies were carried out as well.

## Materials and Methods

The *D,L-*alpha-difluoromethylornithine was purchased from Sigma Aldrich-Merck (Poznan, Poland), and used without further purification. The single crystals, suitable for X-ray diffraction experiment, were obtained by slow evaporation of its solution in water at room temperature.

### Crystal Structure Determination

The crystal data for compound **1** was collected on XtaLAB Synergy, Dualflex, Pilatus 300K diffractometer using graphite monochromated Cu*K*α radiation (λ = 1.54158 Å) at a temperature 100 K, with data collection using Crysalis, and structure determination and refinement by Olex2 (Dolomanov et al., 2009). The structure was determined with the XT (Sheldrick, 2015) structure solution program using Intrinsic Phasing and refined with the XL (Sheldrick, 2008) refinement package using Least Squares minimization. The compound crystallizes in the orthorhombic space group, *P*ca2_1_, with one molecule in the independent unit. The most important crystallographic data are summarized in Supplementary Table S1. Geometrical calculations were done by the PLATON program (Spek, 2009), while figures of crystal structures and analysis of H-bond graph sets by Mercury (Macrae et al., 2008).

CCDC no. of the title compound **1** is 2068871. File can be obtained free of charge from the Cambridge Crystallographic Data Center via www.ccdc.cam.as.uk/data_request/cif.

### DFT Study

The geometries of neutral DFMO (2-(difluoromethyl)ornithine), its protonated cations as well as of corresponding hydrate and solvate (2-(difluoromethyl)ornithine hydrochloride hydrate**)** in the ground singlet state have been completely optimized using the Gaussian 09 program (Frisch et al., 2011). The stability of the optimized structures was tested by vibrational analysis (no imaginary vibrations). For calculations of stable conformers in both gas-phase and aqueous solution the density functional theory level of theory (Parr and Wang, 1994; Neumann et al., 1996; Bickelhaupt and Baerends, 2000) using the M06 hybrid functional (Zhao and Truhlar, 2008) and the polarized triple-ζ 6–311++G(d,p) basis sets (Hehre et al., 1986) for all atoms were used. The Conductor-like Polarizable Continuum Model (CPCM) (Klamt and Schűman, 1993; Barone and Cossi, 1998; Cossi et al., 2003) was exploited for the evaluation of the effect of hydration on the structure of the conformers studied. The gas-phase and solvated state molecular structures obtained by theoretical calculations were compared and discussed with the X-ray structure of these compounds in the crystalline state.

### Hirshfeld Surface Analysis, Molecular Electrostatic Potentials and Energy Frameworks

Hirshfeld surface (HS) analysis is a useful tool in providing information not only on nature of close non-covalent interactions but also on the topology of the ligand surface (molecular shape), electrostatic complementarity, interaction surfaces *via* visualization of the boundaries of a molecule (shape) and inter-contacts with adjacent molecules within the crystal (Spackman et al., 2016). Recently, HS methodology has been tested in the extended context – into the protein environment. It can be used to profile the shape of the protein pocket and generate the HS (with mapped electrostatic potential) around the ligand molecule (extracting the model data from the CSD) (Spackman et al., 2019).

Here, a thorough, either qualitative or quantitative, analysis of supramolecular interactions in **1** and **2** was carried out by HS approach (Spackman and Jayatilaka, 2009) using the CrystalExplorer 17.5 (Mackenzie et al., 2017) program, based on the X-ray crystallographic information data. 3-D HS maps and 2-D fingerprint plots (Spackman and McKinnon, 2002) were generated. The bond lengths of the hydrogen atoms were normalized to standard neutron diffraction values. The color scheme of the surface regions are related to the normalized contact distance, denoted as *d*_*norm*_ (a property reflecting the interactions of the molecule) (Spackman et al., 2016), defined in the context of the van der Waals (vdW) radii of the atoms and *d*_*e*_, *d*_*i*_, the distances from the HS to the nearest atom outside and inside the surface, respectively. Red areas characterize contacts shorter than the sum of vdW radii, white – equal to the sum of vdW radii, while blue — longer than the sum of vdW radii.

The electrostatic potential was calculated, and mapped on HS, using wave function STO-3-G basis sets at the Hartree-Fock theory level over the range of -0.025 au (Spackman et al., 2008).

The energies of intermolecular inter-contacts for the 3D energy framework construction were calculated in the same program by use of single-point molecular wave functions at B3LYP/6–31 G(d,p) level of theory, within a cluster of radius of 3.8 Ǻ (Mackenzie et al., 2017; Turner et al., 2017). The energy benchmark was calculated using Mackenzie’s method to scale energy frameworks. Quantitative analysis of interactions was performed on the base of the equation:

$$E_{tot}\;=\;k_{ele}\;E\;{`}_{ele}\;+\;k{\;}_{pol}\;E`{\;}_{pol}\;+\;k{\;}_{dis}\;E`{\;}_{dis}\;+\;k{\;}_{rep}\;E{`}_{rep}$$

where *k* characterizes scale factor values, *E`*_*ele*_ – the electrostatic energy, *E*`_*pol*_ – the polarization energy, *E`*_*dis*_ – the dispersion energy and *E*`_*rep*_ – the exchange repulsion energy (Mackenzie et al., 2017).

### Enrichment Ratio

In the crystal packing, privileged and disfavored interactions formed by the pair of elements (X,Y) can be analyzed using the enrichment ratio (ER) (Jelsch et al., 2014). ER is a ratio between the interactions within the crystal and interactions derived from the theoretical calculations (HS analysis). The proportion S_X_ on the molecular surface, including the proportion of HS contacts (C_XY_) can be described by the equation:

$$S_{X}\;=\;C_{\mathrm{XX}}\;+\frac{1}{2}\sum_{Y\neq X}C_{\mathrm{XY}}\;\left(\sum_{X}S_{X}=1\right).$$ (1)

Next, the ratio of interactions, and consequently ER, can be defined as:

$$R_{\mathrm{XX}}=S_{X}S_{X}\;\mathrm{and}\;R_{\mathrm{XY}}=2S_{X}S_{Y}\;\left(\sum_{X}R_{\mathrm{XY}}+\sum_{Y\neq X}R_{\mathrm{XY}}=\;1\right).$$ (2)

$$ER_{\mathrm{XY}}\;=\frac{C_{\mathrm{XY}}}{R_{\mathrm{XY}}}$$ (3)

ER > 1 when the pairs of elements have high tendency to form contacts within the crystal, and ER < 1 when pairs avoid contacts with each other.

### SwissADMET Profile

Absorption, distribution, metabolism, excretion, toxicity (ADMET) profiles for analyzed compounds and analogues of DFMO were evaluated using the pkCSM web-platform (Pires et al., 2015; Guan et al., 2019). The information was supplemented by other pharmacological parameters analyzed by SwissADME web-based interface, provided by the Molecular Modeling Group of the Swiss Institute of Bioinformatics (Daina et al., 2017).

### Molecular Docking Preparation

Molecular docking studies to establish favorable ligand binding geometries for both enantiomers of eflornithine (DFMO, α-difluoromethylornithine), namely (2*S*)-(–)2,5-diamino-2-(difluoromethyl)pentanoic acid (*L*-DFMO) and (2*R*)-(+)-2,5-diamino-2-(difluoromethyl)pentanoic acid (*D*-DFMO) as well as the developed DFMO-based derivative, namely (2*S*,5*S*)-5-amino-2-(difluoromethyl)-6-fluoro-2-[(fluoromethyl)amino]hexanoic acid (DFMO-analogue), were carried out on a four CPUs-based desktop PC computer equipped with AMD Phenom™ II X4 965 Processor 3.40 GHz and 32 GB of RAM on a Microsoft Windows 10 Professional 64-bit operating system using AutoDock Vina vs. 1.1.2 program for Windows (http://autodock.scripps.edu/) (Trott and Olson, 2010). The respective ligand molecules *L*-DFMO, *D*-DFMO and DFMO-analogue in non-ionizable form were prepared at first with ChemAxon MarvinSketch vs. 14.9.1.0 (http://www.chemaxon.com/marvin/) using general “Cleaning in 3D” option to assign with proper 3D orientation and then calculating conformers with MMFF94 force field parameters and saved as .pdb files. To obtain the minimum energy conformation for docking studies, the initial geometries of the afore-pretreated ligands were additionally optimized in Avogadro version 1.2.0. (http://avogadro.cc/), after adding all the hydrogens to the structure, and saved as .mol2 files. The energy of the ligand molecules was minimized using built-in feature of Avogadro including General Amber Force Field (GAFF) (Wang et al., 2004) with Steepest Descent Algorithm (100 steps). The minimum conformation energies obtained for each ligand were as follows: *E*_calc.(*L*-DFMO)_ = –209.069 kJ/mol, *E*_calc.(*D*-DFMO)_ = –209.452 kJ/mol, and DFMO-analogue *E*_calc.(DFMO-analogue)_ = –204.022 kJ/mol. The visualization of the optimized geometries were performed using molecular visualization software, POV-Ray for Windows v3.7.0. msvc10.win64 licensed under the terms of the GNU Affero General Public License (AGPL3) (see Supplementary Figure S2 in Supplementary Material). Next, the Gasteiger partial charges were calculated with AutoDock Tools vs. 1.5.6 (ADT, S3 http://mgltools.scripps.edu/) (Trott and Olson, 2010), whereas all torsion angles for each enantiomer of DFMO as well as for the respective DFMO-analogue were considered flexible, and all the possible rotatable bonds (8 out of 32 for DFMO enantiomers and 10 out of 32 for the DFMO-analogue), and non-polar hydrogen atoms were determined. The final ligands’ files were saved as PDBQT files (.pdbqt format), and were ready for docking procedure.

The crystallographic structure of human ornithine decarboxylase (h-ODC, PDB code: 2OO0) (Dufe et al., 2007) of the highest known resolution (1.90 Å) was downloaded from Brookhaven RCSB Protein Data Bank (PDB database, http://www.rcsb.org/pdb/). To avoid steric clashes within the model the crude target protein .pdb file was prepared by UCSF Chimera vs. 1.11.2 package (http://www.cgl.ucsf.edu/chimera/) (Pettersen et al., 2004) by removing all nonstandard molecules including conserved crystal water molecules (HOH), cofactor [pyridoxal-5′-phosphate (PLP)] and other small non-protein ligands [i.e., pentane-1,5-diamine (cadaverine, N2P), 3-aminooxy-1-aminopropane (APA; with ligand code: XAP), acetate ion (ACT)], respectively. For comparison of docking protocols, the protein with preserved PLP in active cavity was also prepared. Next, the polar hydrogen atoms were added and Gasteiger charges were calculated with AutoDock Tools 1.5.6 package using its standard utility scripts, and then the final protein file was saved as a PDBQT file. Next, a searching “grid box” was set by using AutoGrid function to perform docking in a (40 × 40 × 40 Å)-unit grid box (final size space dimensions *x* = 40 Å, *y* = 40 Å, *z* = 40 Å), centered on the catalytic cavity located in one of the subunits (namely “chain A”) of the homodimeric h-ODC as target coordinate (center_x = −33.462; center_y = 18.513; center_z = −23.794) with a grid spacing of 0.325 Å.

### Molecular Docking Procedure and Validation

At first, to validate the molecular docking protocols re-docking of the co-crystallized small molecule inhibitor, that is 1-amino-oxy-3-aminopropane (APA), into h-ODC (PDB access code: 2OO0) protein was performed. The docked complexes were superimposed to the original crystal structure to calculate the values of the root mean square deviation (*rmsd*). The re-docking of 2OO0 target protein structure and APA ligand reproduces the original pose with 1.48 and 2.73 Å *rmsd*, respectively. The average *rmsd*-value reaching 2.11 Å indicates that our docking methodology is adequate and can be utilized to search small molecule inhibitors of h-ODC. The overlaps between the docked pose of APA in h-ODC with retained PLP cofactor and the X-ray structure for h-ODC-APA complex are shown in Supplementary Figure S3 (Supplementary Information). Docking was performed into a rigid protein as well as using advanced protein flexibility by specifying flexible side chains. Each docking was performed with an exhaustiveness level of 48 concerning global search. For each ligand molecule 100 independent runs were performed using the Lamarckian Genetic Algorithm (GA) with at most 106 energy evaluations and a maximum number of generations of >27,000 Å^3^ (the search space volume). The rest of the docking parameters including the remaining Lamarckian GA parameters were set as default using the standard values for genetic Vina algorithms (the posed dockings were below 5.00 Å *rmsd*). The docking modes of each ligand, that is: *L*-DFMO, *D*-DFMO, and DFMO-analogue, were clustered and ranked based on a mutual ligand–protein affinity expressed as absolute free binding energies [Δ*G*_calc_ (kcal/mol)] as well as the *rmsd*-values in both modes regarding *rmsd* lower bound (l.b.), and *rmsd* upper bound (u.b.), respectively. The rmsd were computed referring to the input structure submitted to docking simulations. For h-ODC without PLP-cofactor the used random seed amounted to: (i) –324942292 for *L*-DFMO, (ii) –595761544 for *D*-DFMO, and (iii) –2089770112 for the DFMO-analogue; whereas for h-ODC with PLP-cofactor the used random seed amounted to: (i) +1194314488 for *L*-DFMO, (ii) –844931584 for *D*-DFMO, and (iii) –1490556468 for the DFMO-analogue. The best nine poses (modes) were selected according to AutoDock Vina scoring functions mainly based on binding energies and show mutual ligand–protein affinity (kcal/mol). For the protein with the cofactor in its structure, except considering binding energies, also the proximity of amine moiety of the halogenated ligands to electrophilic carbon atom of the formyl group of PLP was examined. Each binding mode was manually inspected to select only those conformations of the ligand molecule, which accommodated in the h-OCD catalytic cavity in the highest possible proximity to the *L*-ornithine substrate-binding site according to the crystal structure of h-ODC co-crystallized with both the PLP-cofactor and the APA inhibitor deposited as PDB: 2OO0. The results of docking scoring of the respective ligands to h-OCD without or with the PLP-cofactor are collected in Supplementary Tables S2–S8 (Supplementary Material), respectively. Additional data including the most important amino acid residues of h-ODC (PDB: 2OO0) involved in a hydrogen bond to the respective ligands as well as the results of the measurements of dihedral angels of *L*-DFMO and its analogue (**9**) docked to h-ODC (PDB: 2OO0) are available in Supplementary Tables S9–S13. The results generated by AutoDock Vina including optimized binding poses of all ligands in hypothetical complexes with the h-ODC protein as well as critical polar contacts between the respective atoms of those ligands and the receptor molecule (h-ODC, 2OO0) were visualized using the PyMOL Molecular Graphics System software, version 1.3, Schrödinger, LLC (https://www.pymol.org/). Visualization of ligands interaction diagrams were performed using Maestro Version 12.6.144, MMshare Version 5.2.144, Release 2020-4, Platform Windows-x64 for academics of the Schrödinger suite (Supplementary Figure S4). For comparison the protein-ligand interactions of the docked model were also analyzed by using freeware for academia BIOVIA Discovery Studio Visualizer 20.1.0.19295 software (Dassault Systèmes Biovia Corp.; https://www.3ds.com) (Supplementary Figure S5).

## Results and Discussion

### Crystal and Molecular Structure of the Title Compound

**FIGURE 2:**
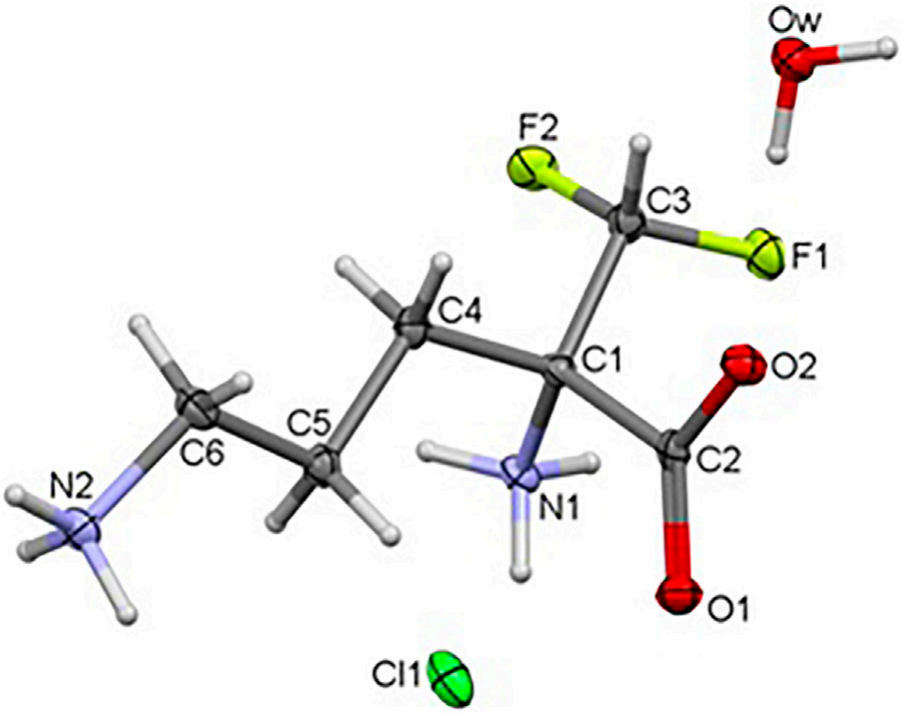
Crystal structure of the title compound **1**.

The title compound **1** crystallizes in the orthorhombic system, in the noncentrosymmetric *P*ca2_1_ space group with unit cell parameters *a* = 10.41500 (10), *b* = 8.96290 (10), *c* = 10.70340 (10) Å. Crystal data as well as refinement parameters are summarized in Supplementary Table S1. The perspective view of the molecular structure of **1** with the non-hydrogen atom labeling scheme is presented in Figure 2. As depicted in the diagram, the asymmetric unit of **1** consists of one independent DFMO cation, a chloride ion and one water molecule. Bond length and angles exhibit normal values for ornithine derivatives (Cambridge Structural Database, Version 5.41) (Allen, 2002; Groom et al., 2016). Selected bond lengths and angles are listed in Supplementary Table S14, while geometrical parameters of H-bonds in Supplementary Table S15. The structure is stabilized by intra- and intermolecular either classical or non-classical O-H^…^O(Cl), N-H^…^O(Cl/F), C-H^…^O hydrogen bonds between each of DFMO, Cl^−^ and water moieties, in the range of 2.6775 – 3.4213 Å. All H-atoms of both NH_3_^+^ groups and O atoms of COO^−^ group are involved in the H-bonding as donors and acceptors, respectively.

### DFT Study

The geometry optimization calculations were carried out using the hybrid meta exchange-correlation functional M06, which describes the molecular structure of organic compounds with high accuracy (Jacquemin et al., 2010; Zhao and Truhlar, 2011). Harmonic vibrational frequencies calculations of the optimized conformers computed in both surroundings confirmed that all of them correspond to minima of their potential energy surfaces. The initial geometries for the DFT geometry optimizations were obtained from the X-ray structure of the complex of this compound bound to human arginase I (Ilies et al., 2011). Because X-ray crystal structures of human arginase I complexed with the DFMO reveal the exclusive binding of the *L*-stereoisomer, this stereoisomer was considered in our calculations only. DFMO, when bound on the enzyme, is present in the zwitterionic form with basic *N*-amino group protonated (Ilies et al., 2011) (Supplementary Figure S6). The calculations were carried out for both, neutral DFMO molecules and its protonated cation at the terminal basic nitrogen atom N2. The important structural parameters are shown in Supplementary Table S16.

The M06 optimized structure of DFMO molecule in the both, gas-phase and hydrated state corresponds to the structure with the linear arrangement of the flexible C-C-C-C-C-C-N chain. The all-trans configuration is manifested by dihedral angles α, β and γ which are close to 180°degrees. The solvent affects the equilibrium structure only slightly (Supplementary Table S16).

The structure of neutral DFMO conformer resulted from the DFT calculation fits well with the experimental species of DFMO when bound at the human arginase I receptor. The DFMO molecule at this receptor is slightly bent, dihedral angle β[C(1)-C(4)-C(5)-C(6)] is of 146° and the C-C-C-C-N chain possess anticlinal conformation. The molecular superposition of the X-ray structure of biologically active conformation of DFMO (3gn0.pdb) and the corresponding thermodynamically stable structure isolated molecule is displayed in Supplementary Figure S7. Thus, the biological environment changes the molecular structure of this drug only slightly.

In clinical practice, eflornithine hydrochloride is used. We prepared suitable crystals of DFMO in the form of the monohydrate of eflornithine hydrochloride, **1**. In the crystal state DFMO and HCl form a 1:1:1 complex with water. The hydrochloride acid forms an ion pair of the Cl^−^ ··· ^+^H_3_N type with the basic terminal amino group of DFMO. The chloride anion in the crystal structure is coordinated with the basic center of DFMO by means of an amino group of another molecule. Crystal water contributes to the stabilization of the molecular crystal via the system of intermolecular hydrogen bonds.

**FIGURE 3:**
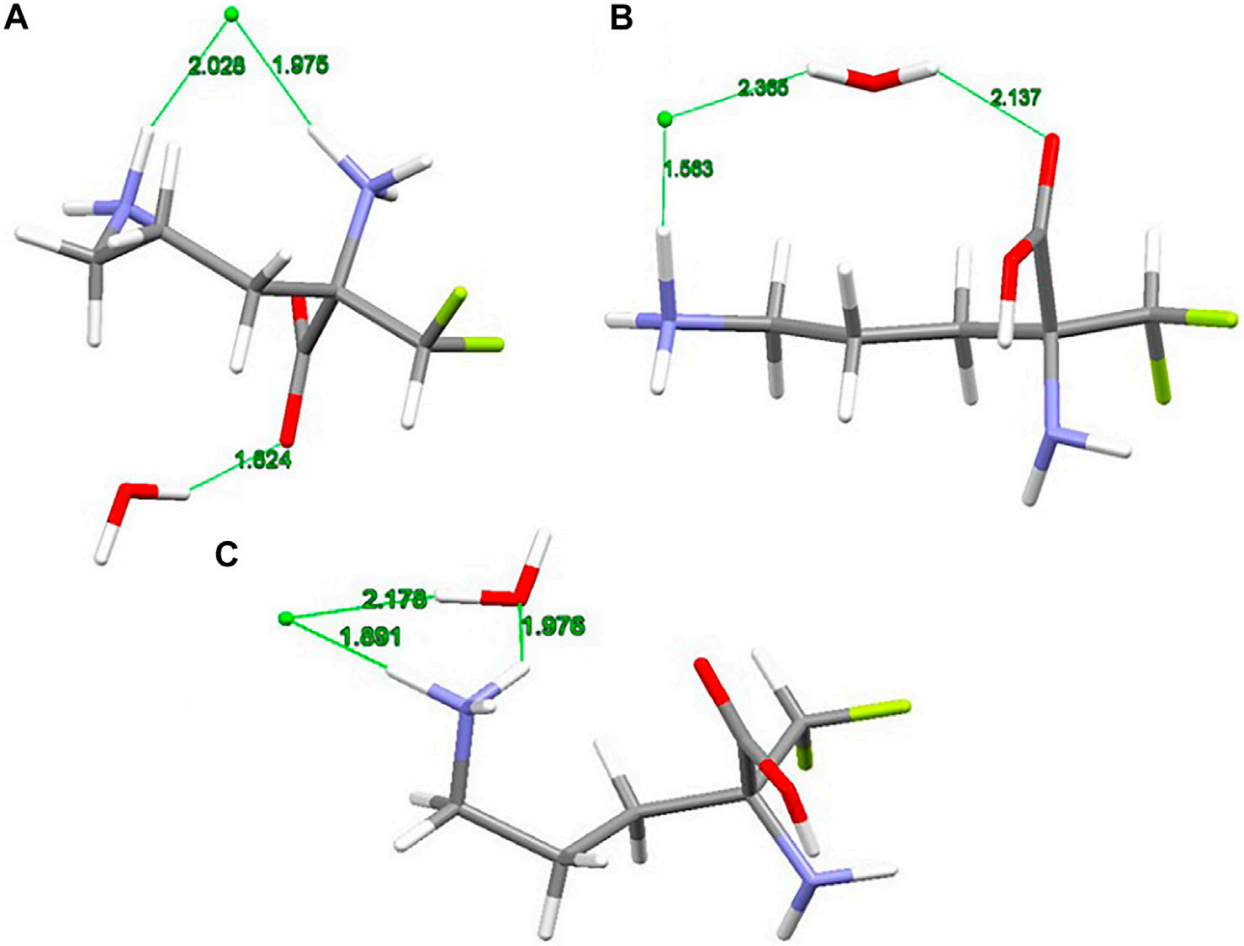
RM06/6-311++G(d,p) optimized structures **(A**, **B** and **C)** of 2-(difluoromethyl)ornithine hydrochloride species studied, distances are in Å.

The X-ray structure of the 2-(difluoromethyl) ornithine hydrochloride hydrate, 1, was used as a starting geometry in our DFT calculations. However, in the absence of intermolecular crystal forces, the complex coordination of chloride anions and water molecules upon geometry optimization of a single 2-(difluoromethyl) ornithine hydrochloride hydrate dramatically changes its geometry. Thus, the geometry optimizations resulted in three different structures denoted as A, B and C, respectively (Figure 3). The complex A mimics the geometry of DFMO in the solid state. The amino (–NH_2_) and carboxyl (–COOH) functional groups of this complex form a zwitterion and the Cl- ion is coordinated to both cationic (–NH_3_^+^) groups. Water forms the hydrogen bond with the anionic carboxyl moiety.

Different starting geometries of 2-(difluoromethyl)ornithine hydrochloride hydrate resulted in two additional stable complexes B and C, Figure 3. The amino (–NH_2_) and carboxyl (–COOH) functional groups in these systems are neutral and stabilized via the intramolecular hydrogen bond of the N-H···O=C type. The water molecule takes part on the hydrogen bonds acting both as proton donor and proton acceptor. In the complex B the chloride anion is coordinated by the carboxyl group of the parent molecule by means of the water molecule.

The relative stability of A, B and C structures of 2-(difluoromethyl)ornithine hydrochloride hydrate 1 is presented in Supplementary Table S17. Based on the relative Gibbs energies the structure B is the most stable species (Figure 3) both in the gas-phase and in the aqueous environment. However, the differences in relative stability of these species are low, and in the gas-phase all three complexes may coexist with almost equal probability. The aqueous environment clearly prefers the structure B. In the crystal structure of 2-(difluoromethyl)ornithine hydrochloride hydrate the entire molecule is nearly planar and close to the conformation of the thermodynamically stable structure B, Supplementary Table S17.

### Investigation of Intermolecular Interactions

#### Hirshfeld Surface Analysis

To gain additional information about diverse types of supramolecular interactions and their contribution to the title crystal structure of **1** in comparison with ornithine **2**, 3D HSs and 2D fingerprint plots (FPs) were thoroughly investigated. In **1**, the bright red circular spots on HS mapped over *d*_*norm*_ indicate the highest close interactions such as O^…^H/H^…^O (Supplementary Figure S8). Red zones on the *d*_*i*_ and *d*_*e*_ surfaces define close interactions in relation to the internal and external surface of the molecule, respectively. The *shape index* property is helpful in visualization of stacking arrangement (*via* blue-red triangles). However, this kind of contacts is lacking in analyzed structures. The fragment patches on the HSs indicate the coordination environment of the molecule within the crystal. The coordination number is characterized by the *curvedness* of the HS. The flat regions of the HS have low values of *curvedness*, while the sharp areas – high values of *curvedness*, leading to the division of the surface into color patches reflecting inter-contacts among the nearest neighboring molecules.

**FIGURE 4:**
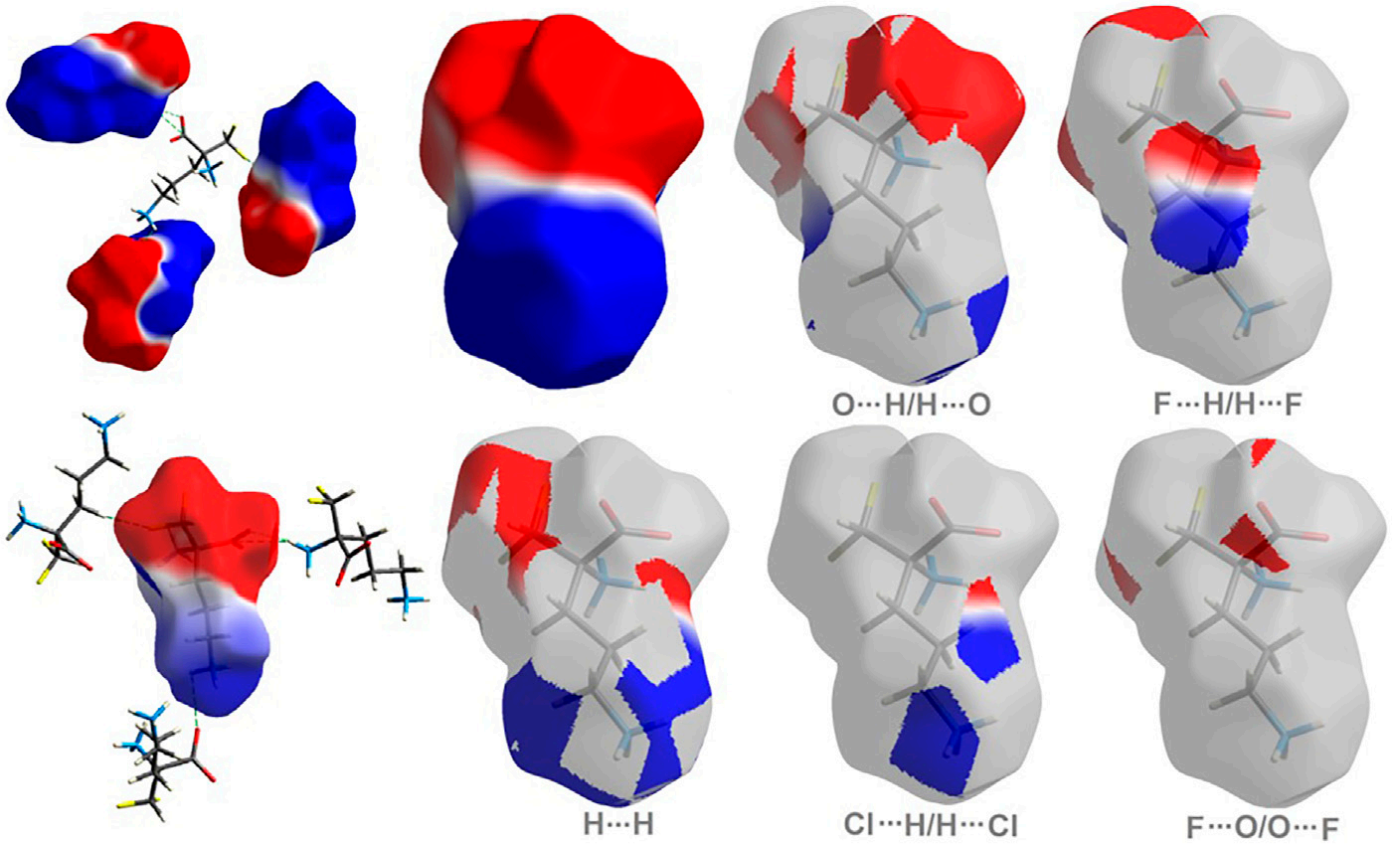
**On the left:** the molecular ESP mapped on the HS of **1**. **On the right:** surface patches identified with decomposed close contacts in crystal **1**. Red regions (acceptors) correspond to the negative electrostatic potential, while blue (donors) to the positive electrostatic potential.

The molecular electrostatic potential (ESP) offers a deeper direct insight into the supramolecular interactions within the crystal (Spackman et al., 2008). The title compound **1** has a dipolar nature, similar to ornithine **2** (Bojarska et al., 2020b), with clearly separated negative (red area) and positive (blue region) electrostatic potentials, representing H-bond acceptor and donor sites, respectively. The electrostatic complementarity of **1** mapped on its HS is demonstrated in Figure 4.

**FIGURE 5:**
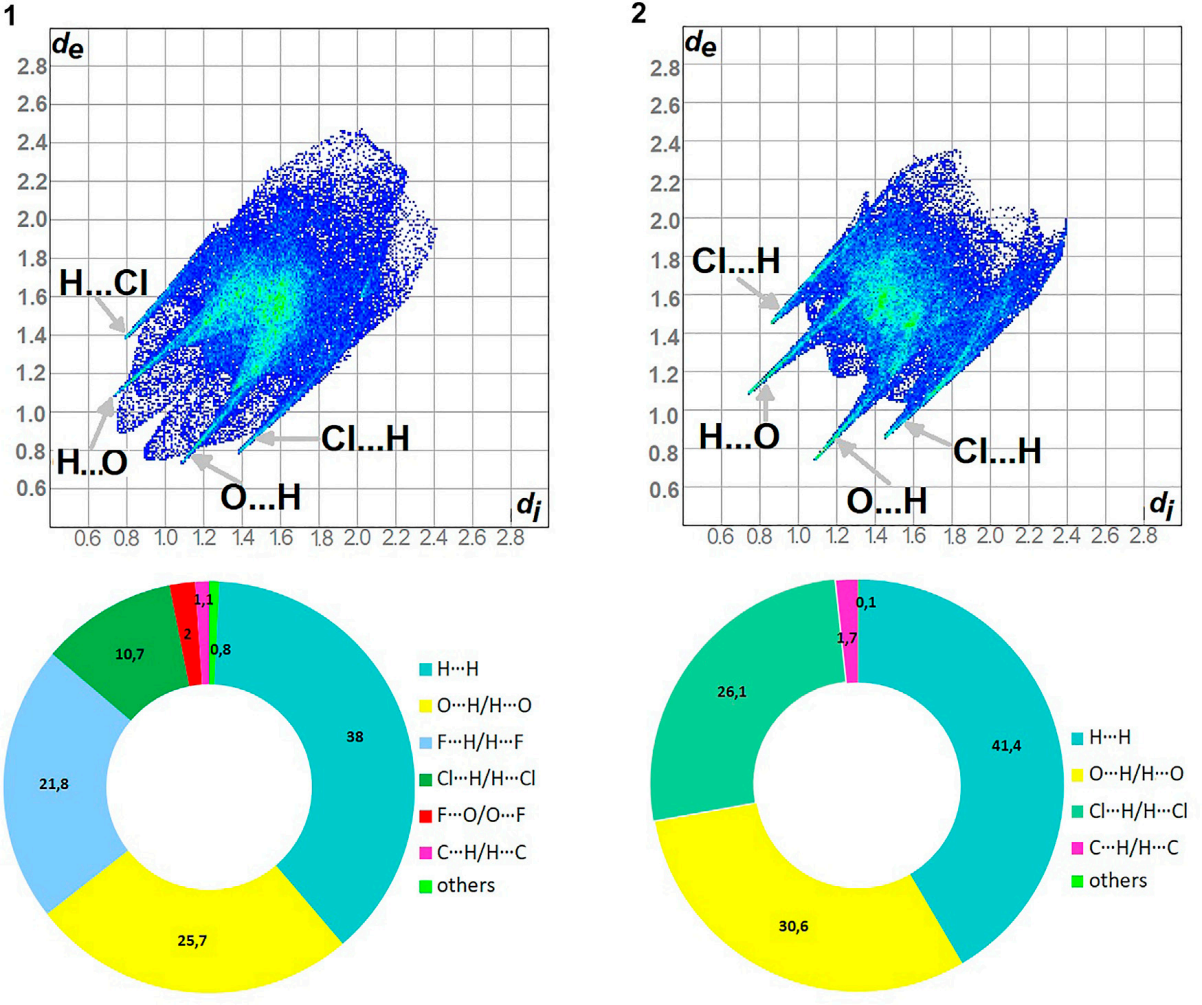
Fingerprint Plots and relative percentage contributions of the diverse supramolecular interactions to the HSs for analyzed crystals (others signifies contacts below 1%).

As shown in Figure 5, H^…^H and O^…^H/H^…^O are the major contributors to the HS in the title compound **1** and **2** as well. Furthermore, F^…^H/H^…^F in **1** and Cl^…^H/H^…^Cl in **2** are also of great importance, at the level of ∼20 and 26%, respectively. Nevertheless, Cl^…^H/H^…^Cl contacts significantly contribute to **1** as well, at the level of 11%. F^…^O/O^…^F in **1** and C^…^H/H^…^C (1.7) in **2** should not be overlooked (2%). On the other hand, in **1** C^…^H/H^…^C factor is only of 1% (Supplementary Figure S9).

### Enrichment Ratio

The enrichment ratios (ER) of the intermolecular interactions in the analyzed crystal structures were calculated on the HS methodology (Jelsch et al., 2014). Results are collected in Supplementary Table S18. Privileged close inter-contacts are as follows:

In **1**: O^…^H/H^…^O, C^…^H/H^…^C, Cl^…^H/H^…^Cl, F^…^H/H^…^F

In **2**: O^…^H/H^…^O, C^…^H/H^…^C, Cl^…^H/H^…^Cl

The highest values of ER are evaluated for the interactions: O^…^H/H^…^O (∼1.5) and Cl^…^H/H^…^Cl (1.5) in **1**, O^…^H/H^…^O, C^…^H/H^…^C and Cl^…^H/H^…^Cl at the level of 1.4 in **2**. The H^…^H contacts are moderately enriched (0.85) despite their highest contribution into the HS in **1** and **2**. The contacts F^…^O/O^…^F in **1** are impoverished, which is consistent with the HS analysis. Furthermore, the C^…^H/H^…^C and F^…^H/H^…^F ones have values of 1.35 in **1**. However, C^…^H/H^…^C are an insignificant contributor in HS of **1**.

### Energy Frameworks

**FIGURE 6:**
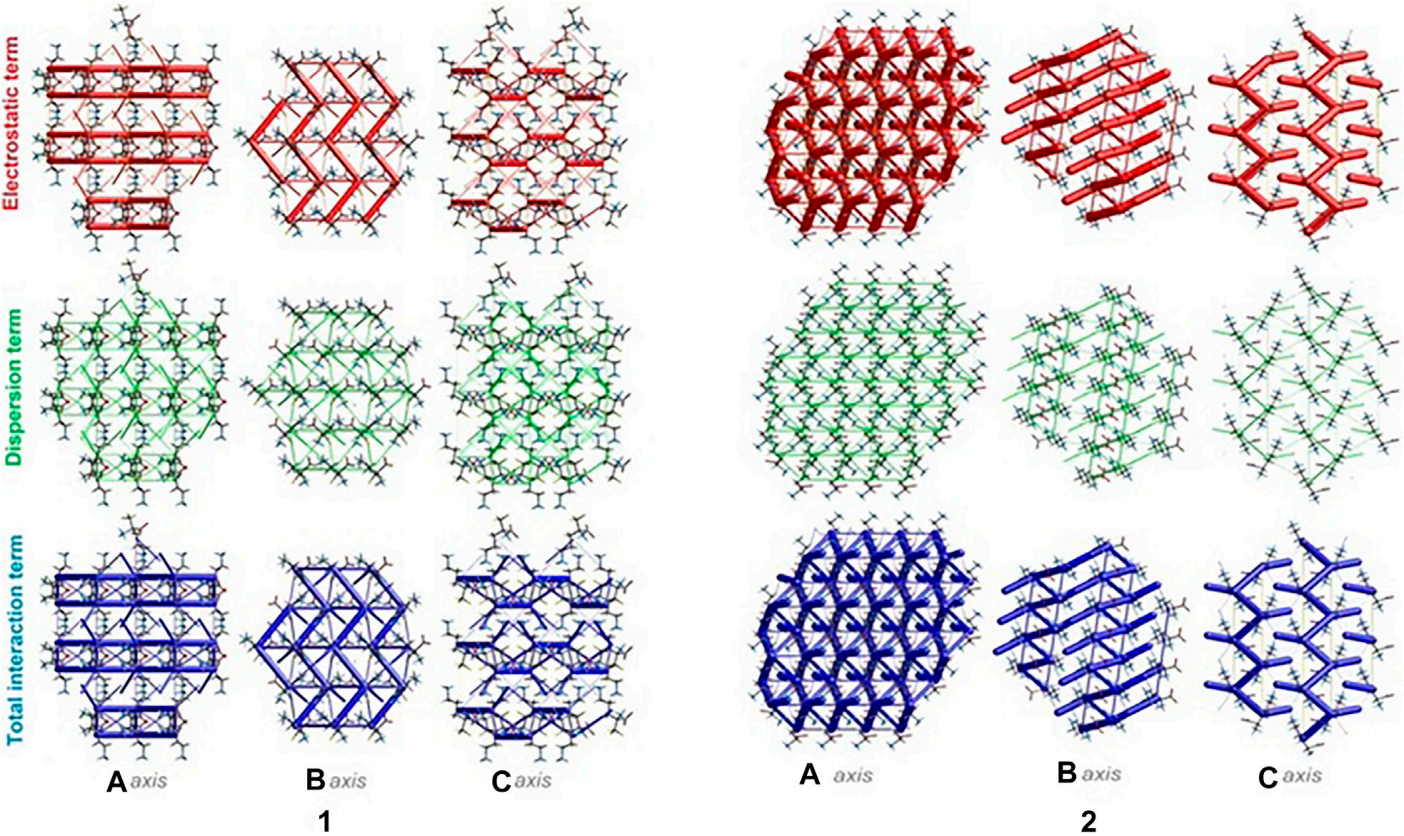
Energy frameworks of crystals **1** and **2** corresponding to the electrostatic and dispersion energy components, and the total energy framework along *a, b* and *c*-axis. The cylinders thickness shows the relative strength of molecular packing.

The energies of intermolecular interactions were investigated (Supplementary Table S19) and the 3D topology of the crystal packing was visualized graphically *via* energy frameworks (Figure 6) to better understand the packing of the crystal structure and the supramolecular rearrangement in all space directions. The width of the cylinders (a size of 100) shows the relative strength of the energy between the molecules. The cylinders with the largest radius demonstrate the highest energy values. The red, green and dark blue tubes denote the electrostatic, dispersive and total energy, respectively. The molecular interactions in relation to the central (reference) molecule in a cluster of radius 3.8 Ǻ are shown in Supplementary Figure S10 (Supplementary Table S20). The molecular pairs are uniquely color-coded. The energies of the molecular pair-wise interactions were used to evaluate the net interaction energies. Generally, the intermolecular energy analysis revealed a significant contribution of the electrostatic energy term, related to the strong classical interactions, in the stabilization of the crystal lattice of both investigated systems. Nevertheless, in **1**, additional weak interactions are observed, which should not be overlooked. More specifically, the total energies include the electrostatic term, namely *E`*_*ele*_ = -134/-359.1 kJ/mol, the polarization term *E`*_*pol*_ = -69.5/-134.2 kJ/mol, the dispersion term *E`*_*dis*_ = -124.7/-106.5 kJ/mol, the repulsion term *E'*_*rep*_ = 161.9/370.5 kJ/mol and the total energy *E`*_*tot*_ = -201.8/-342.8 kJ/mol, for **1** and **2,** respectively. In the light of these findings, in **1**, the difference between the electrostatic and dispersion terms (responsible for weak interactions) is insignificant. In consequence, the topology of the electrostatic and dispersive energy terms in the systems **1** and **2** are quite different.

### Exploration of Supramolecular System of DFMO: Identification of H-Bonding Motifs

Supramolecular chemistry focuses on non-covalent molecular recognition resulting in supermolecules (Lehn, 1995). The latter are defined as the association of two or more chemical moieties into well-organized entities of higher complexity (Dunitz, 1991). Non-covalent interactions are common in a protein environment. Hydrogen bonding interactions (apart from hydrophobic interactions), and N-H^…^O H-bonds as more frequent than O-H^…^O, are the most popular in protein–ligand complexes (Ferreira de Freitas and Schapira, 2017). Therefore, we can say that bio-complexes are supramolecular bio-systems. The structure of the supermolecule can be defined by supramolecular synthons – structural units formed by synthetic operations requiring non-covalent interactions (Desiraju, 1995). In other words, synthon is a recognition unit based on chemical functionality. Homosynthon exists between the same functional groups, while heterosynthon – between two different functionalities (Vishweshwar et al., 2003; Walsh et al., 2003). The Etter’s H-bond rules (based on electrostatic potentials) were useful to determine donor-acceptor interactions leading to synthons (Etter et al., 1990). It is important to point out that the supramolecular synthon concept is promising in the future design of idealized ligands with effective binding inside the protein pockets *via* matching synthonic functionalities (from corresponding libraries) to the model ligands (Spackman et al., 2019). In this context, the potential transferability of supramolecular synthons from small-molecules to macromolecular bio-systems should be kept in mind (Bissantz et al., 2010; Groom and Cole, 2017; Bojarska et al., 2018a). Notably, supramolecular aspects of amino acids, essential components of proteins, are of prime importance in terms of the drug design and development.

**FIGURE 7:**
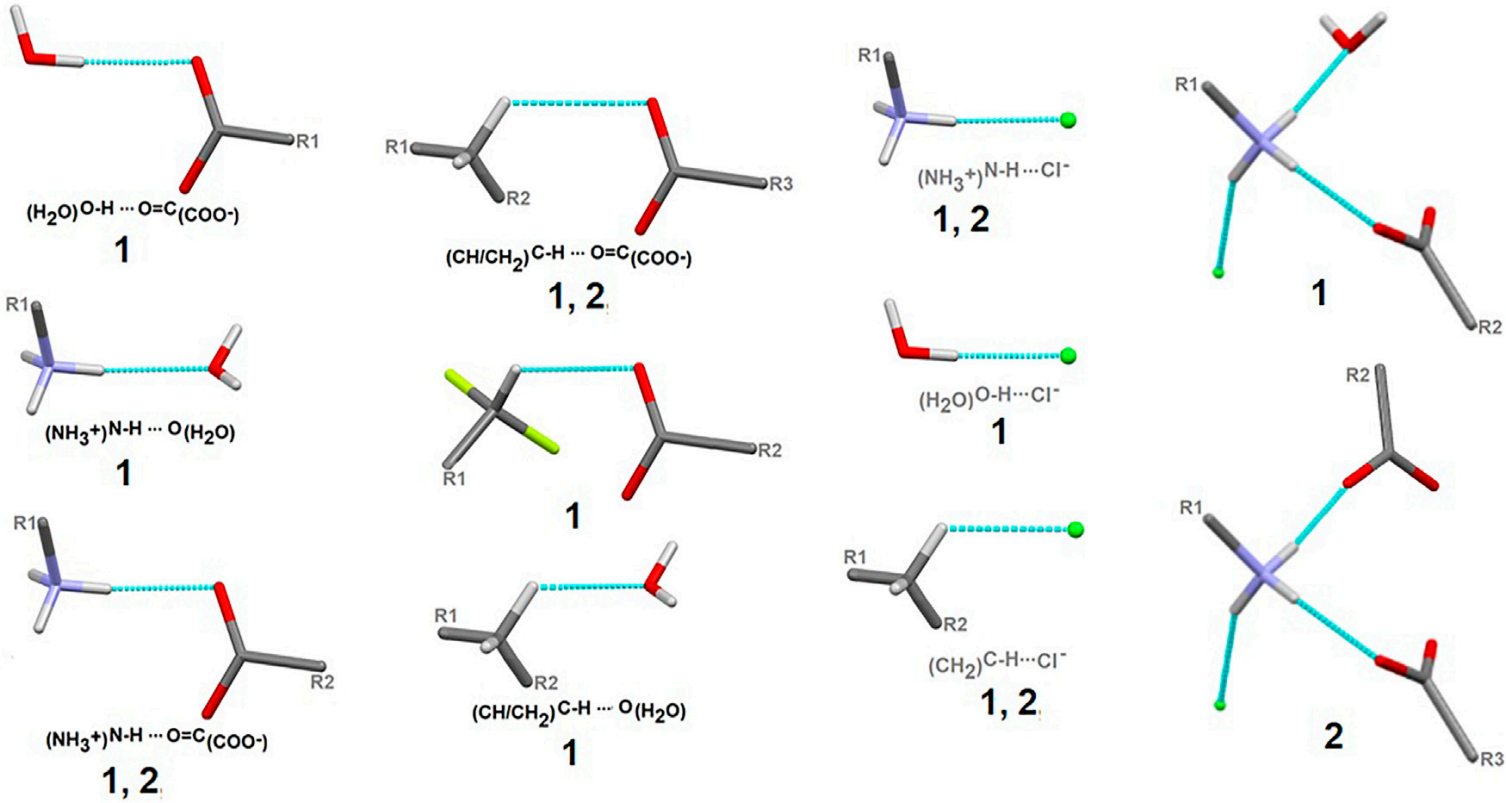
Schematic depiction of representative models of H-bond interactions (synthons) with labels of analyzed crystals in which the corresponding H-bonding pattern is present.

Here, we focus on the survey of synthons driving the self-assembly, their topological properties in the supramolecular landscape in the title compound **1**, in comparison with the ornithine supermolecule, **2**. We report supramolecular relationships and synthon preferences resulting from the interplay of strong and weak interactions. Overall, ubiquitous linear and bifurcated H-bonds are formed in analyzed supramolecular systems. All types of interactions formed by amine and carboxyl groups, such as _(H2O)_O-H^…^O_(COO-)_, _(NH3+)_N-H^…^O_(COO-/H2O)_, _(CH2)_C-H^…^O_(COO-/H2O)_, but also those containing Cl^−^ and H_2_O moieties, such as _(NH3+)_N-H^…^Cl^−^_(CH2)_C-H^…^Cl^−^ and _(H2O)_O-H^…^Cl^−^, are presented in Figure 7. Notably, the amino-carboxy synthons are most important in the protein environment. An interesting additional issue may be the structural arrangement (motif) involving the NH_3_ group, which interacts with the carbonyl group, H_2_O and/or Cl^−^ entities.

**FIGURE 8:**
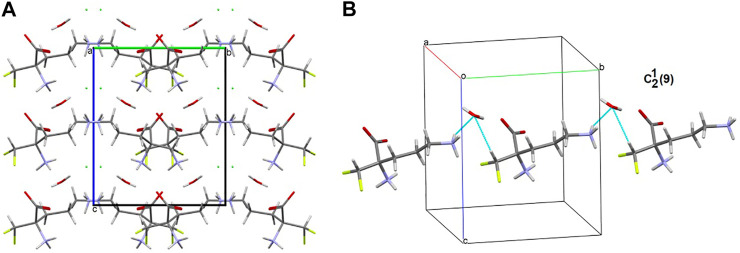
Supramolecular synthon linking DFMO and H_2_O moieties in the crystal structure of **1**.

**FIGURE 9:**
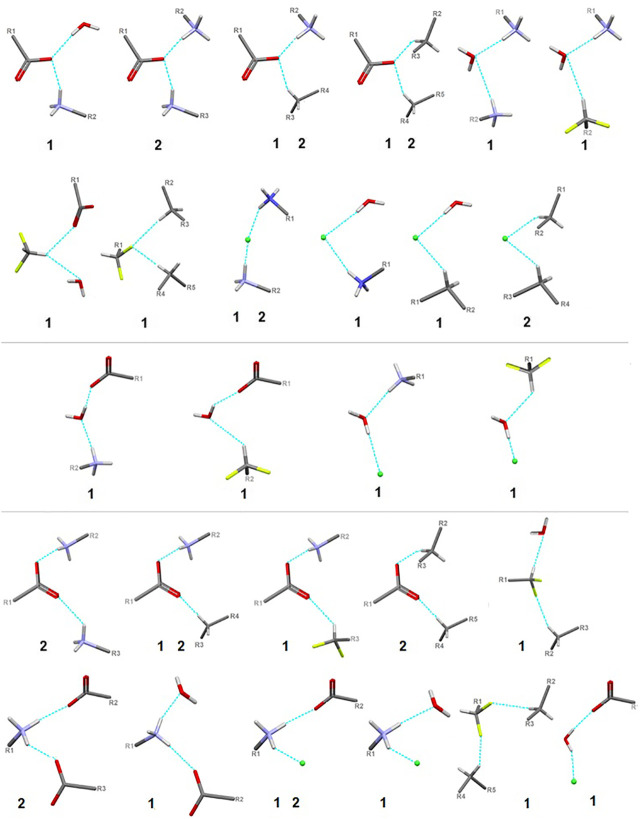
Schematic presentation of all types of interactions in the analyzed structures: above – bifurcated H-bonds (including bifurcated donor and bifurcated acceptors); in the middle – ambivalent; bottom – others (red - oxygen, grey - carbon, blue - nitrogen, greeen - Cl^-^, lime - fluorine).

In the title crystal **1**, *S*(6) and *R* ^2^_2_(7) H-bonding motifs formed by oxygen atoms of COO^−^ group stabilize the structure (Supplementary Figure S11). The primary supramolecular H-bonding interactions are classical strong O(N)-H^…^O=O and weak C-H^…^O(F, Cl) and N-H^…^Cl H-bonds. To be precise, at the first level of the supramolecular architecture, intramolecular cyclic and intermolecular linear H-bond patterns are sustained, namely: *S*(6) by _(CH2)_C-H^…^O=C_(COO)_, *C*(5) by _(NH3+)_N-H^…^O=C_(COO-)_, _(CH)_C-H^…^O=C_(COO-)_ or _(CH2)_C-H^…^F, C(7) by _(CH)_C-H^…^F and *D*(2) by _(NH3+)_N-H^…^O_(H2O)_, _(H2O)_O-H^…^O=O_(COO-)_, _(CH)_C-H^…^O_(H2O)_, _(NH3+)_N-H^…^Cl^−^ or_(CH2)_C-H^…^Cl^−^ (Supplementary Figure S12). H_2_O molecules play supporting roles of bridging DFMO molecules and participate in the formation of supramolecular H-bonding patterns, for example they build the water-linear chain with the *C* ^2^_2_(9) H-bond descriptor, parallel to the *b* axis, (Figure 8). At the second level, diverse types of synthons are observed, including both cyclic and linear homo- and heterosynthons. The latter are dominant. The library of all H-bonding motifs found in the studied crystals is included in Supplementary Table S21. In particular, bifurcated H-bonds are observed (Figure 9). This type of interactions proposed by Albrecht and Corey (in glycine) (Albrecht and Corey, 1939) is represented by the H-bonding pattern, including more than one acceptor/donor (one H-bond donor is bound to two H-bond acceptors, and *vice versa* – two H-bond donors are bound to a single H-bond acceptor). Notably, bifurcated H-bonds are energetically favorable (Feldblum and Arkin, 2014). COO^−^, F, H_2_O, Cl^−^ moieties participate in the formation of supramolecular motifs as a bifurcated acceptor, while NH_3_^+^ CH(CH_2_) as a bifurcated donor. Additionally, it should be mentioned that the water molecule plays an ambivalent role, both of a donor and an acceptor.

**FIGURE 10:**
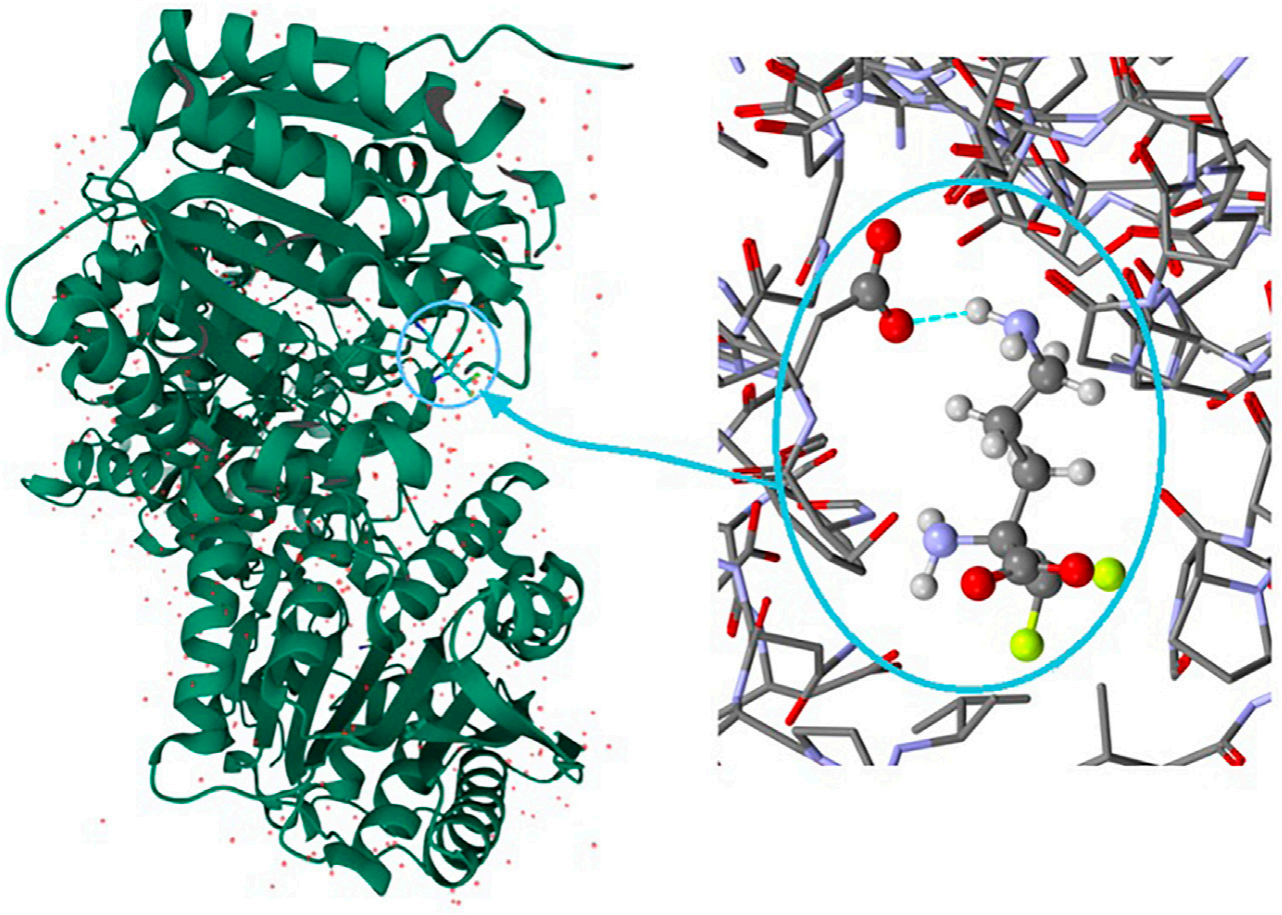
On the left: the bio-complex of arginase with the DFMO ligand. On the right: the supramolecular synthon *D*(2) builded by amino and carboxy groups (PDB code: 3GN0) (Ilies et al., 2011).

It is well known that supramolecular interactions play crucial roles in bio-systems, *inter alia* protein stabilization, molecular recognition, efficiency/specificity of enzymatic reactions (Frieden, 1975; Alberts et al., 1994). A bifurcation (and multi-furcation) is common in bio-systems. The H-bonds between the protein (target) and the ligand (drug) build unique H-bonding patterns, which can be described by synthon descriptors (Sarkhel and Desiraju, 2004; Panigrahi, 2008). In the context of this work, we observed that the bio-complex of arginase with DFMO displays a similar tendency to build the same synthons, such as the *inter alia* discrete chain *D*(2) created by the interactions between amino and carboxyl groups (N-H^…^O-C) (Figure 10) (Ilies et al., 2011).

### Fluorine-Based Synthons

Due to the relevance of fluorine in bio-medicine, we also directed our attention to the identification of the structure-determining fluorine-based H-bonding patterns and careful topological analysis of the involvement of interactions containing fluorine in control of the supramolecular systems. The findings could be valuable in terms of the design of future innovative drugs as well as the advancement of the supramolecular chemistry of short peptides.

**FIGURE 11:**
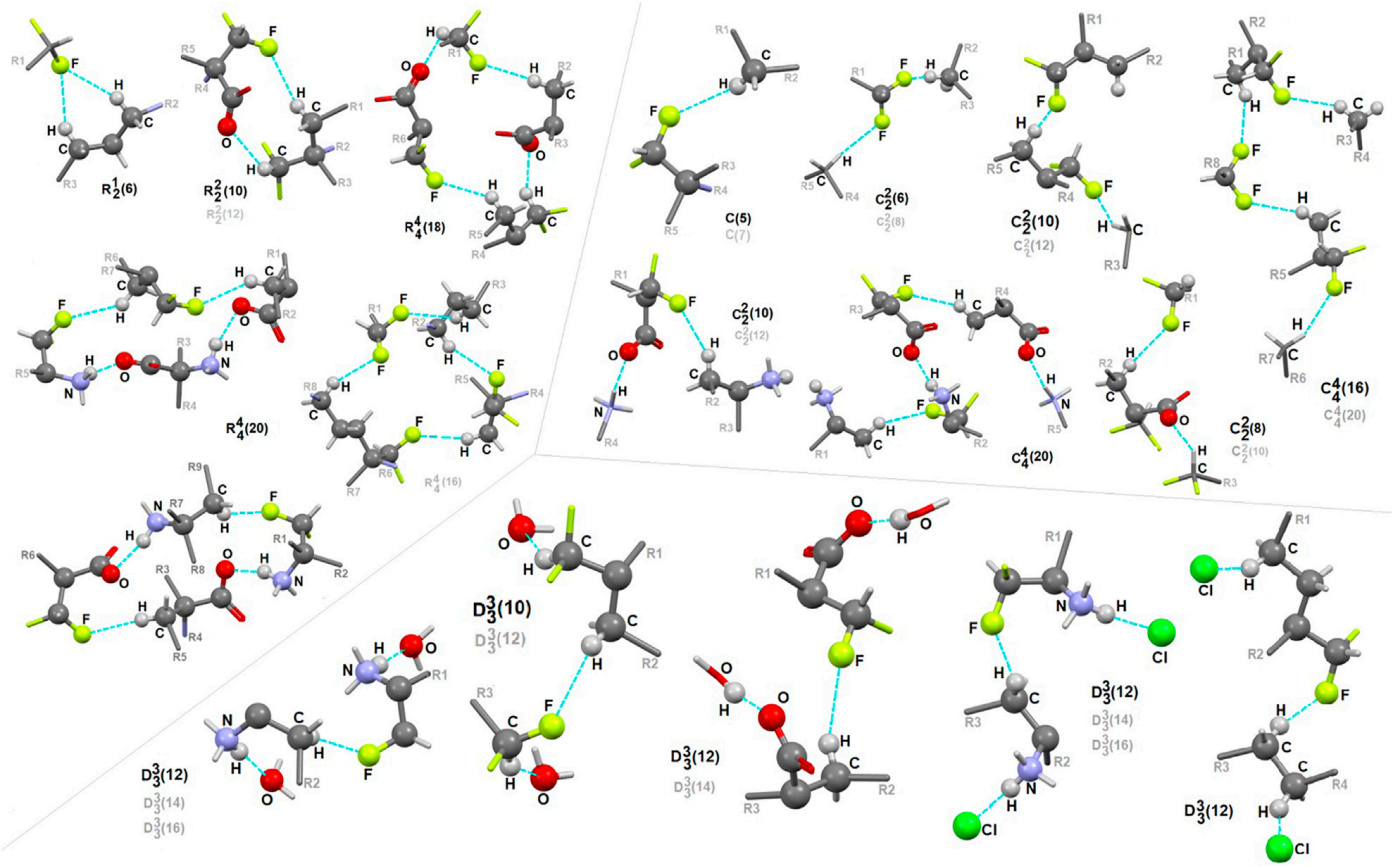
Gallery of fluorine-based supramolecular motifs grouped into ring-, chain-, and finite chain-D- H-bonding motifs, encountered in analyzed supramolecular systems. The atom-numbering scheme for interacting atoms is included. For clarity, side chains were eliminated.

In **1**, the contribution of C-H^…^F interactions (distance below ∼2.6 Ǻ and angle above 120°) at the level of ca. 20% (based on the HS study) is noteworthy. Therefore, stabilization of the structure by defined fluorine-based supramolecular motifs cannot be neglected. Hirshfeld surfaces presenting C-H^…^F patterns are depicted in Supplementary Figure S13 **.** We highlight the effect of numerous H-bonding motifs containing fluorine in relation to the role of diverse/subsequent interactions and their cooperativity in the construction of supramolecular architecture of **1**. The fluorine atom participates through C-H^…^F-C interactions in the formation of H-bonding patterns, both at the first and second levels of the supramolecular structure. In addition, auxiliary either strong or weak (N/C)O-H^…^O and (C)N-H^…^Cl interactions are involved in the formation of fluorine-based synthons at the second level (Supplementary Table S22). 5-membered and 7-membered chains formed by C-H^…^F interactions only are the principal motifs in the studied system. The bifurcated F-atom participates in the building of only the *R* ^1^_2_(6) motif. The structural variety of homo- and heterosynthons, grouped into the rings, chains and discrete finite chains *D*-synthons, are presented in Figure 11. DFMO molecules are assembled into cyclic ring-shaped motifs through the dimer or catemer synthons. On the other hand, the dimer, trimer and pentamer of linear – chain synthons are observed. In the case of discrete chains – D-type motifs, we encountered only tetramer (catemer) units, but the co-operation of solvents such as water and Cl^−^ moieties in this kind of H-bonding motifs should be mentioned. In chain and ring types of H-bond patterns, homosynthons, apart from heterosynthons, are visible.

### Long-Range Synthon Aufbau Modules

**FIGURE 12:**
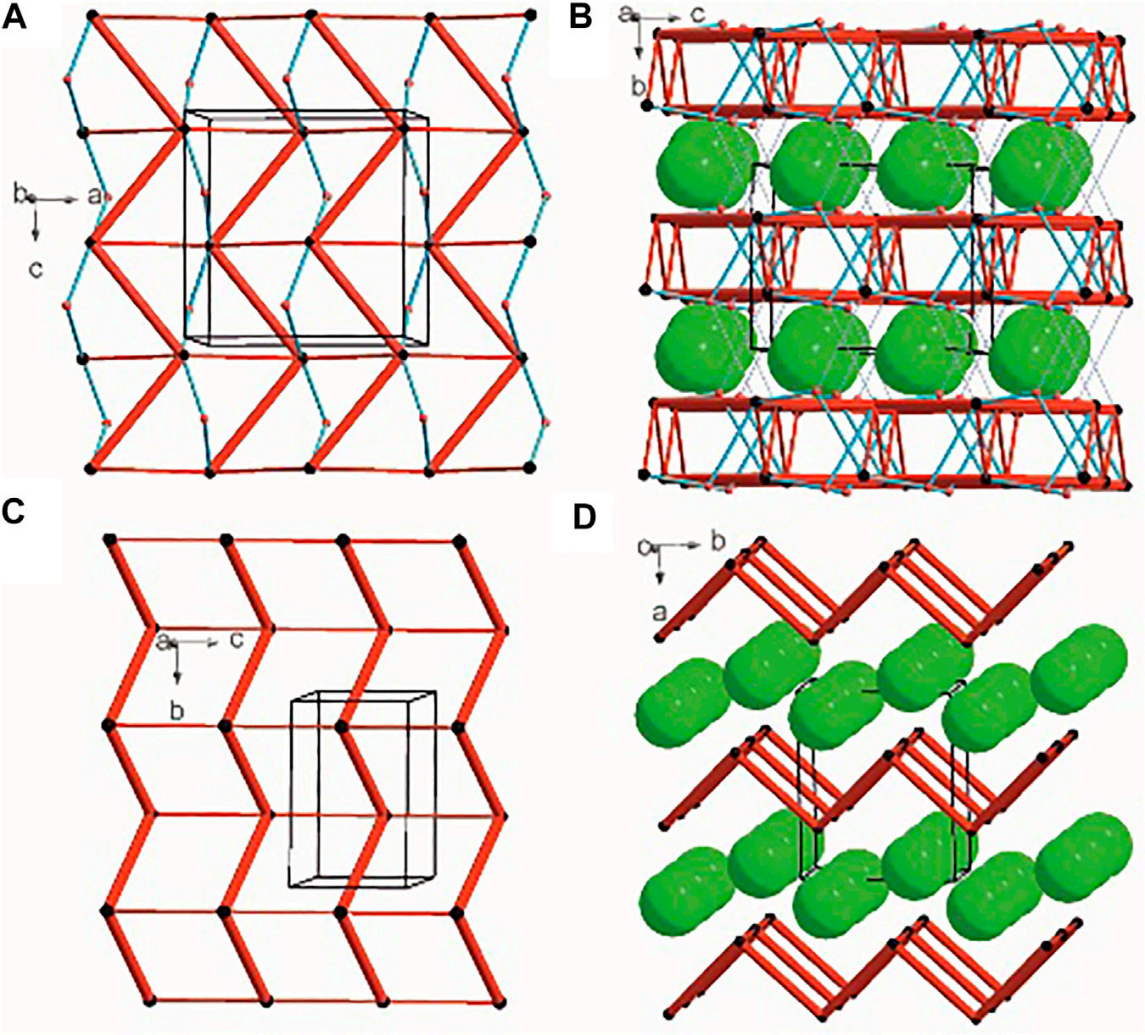
Graph representation of the crystal structure of **1** and **2** showing LSAMs **(A,C)** and their packing **(B,D)**. The black dot represents cations’ center of gravity, red dot – positions of water molecules while green spheres positions of chloride anions. Thick red rods show the most important N-H^…^O interactions, the thin ones — secondary interactions between cations while blue lines stand for interactions with water molecules.

There are three chemical entities in the crystal of **1**: the DFMO cation, the chloride anion, and the water molecule. Both the cation and water molecule act as proton donors or acceptors while the anion is only the acceptor participating in charge-assisted hydrogen bonds. Taking into account the dimensionality of the synthons described at the first level of the graph theory, the smaller species participate only in 0D (definite, D) motifs. In turn, the cations interact *via* N-H^…^O hydrogen bonding forming the first-level *C*(5) motif running along the [001] direction and joining molecules related by the *c*-glide plane. According to Energy Frameworks calculations (Figure 6), this is the most significant interaction in the crystal, and thus might be regarded as a Large Synthon Aufbau Module (LSAM), (Desiraju, 1995; Ganguly and Desiraju, 2010). The symmetry of this supramolecular chain can be described with the p*c11* rod group symmetry (International Tables for Crystallography, 2006). In a crystal, each chain is surrounded by eight others, but at three different distances. The lack of close-to-hexagonal packing indicates the presence of other important interactions between the chains. Indeed, the plethora of weak interactions of C-H^…^O and C-H^…^F type are observed in the [100] direction, joining the cations related by the *a*-glide plane into a zigzag chain. As a result, wavy layers perpendicular to (010) planes are observed, and thus extending the cationic LSAM into two dimensions with the *pba*2 layer group symmetry. Interestingly, the shortest interactions of the cation with water molecules, resulting in the *C* ^2^_2_(7) chain motif along the *2*_1_ screw axis, might be treated as reinforcement of the 2D structure (Figure 12A,B). Between the layers, the anions are located, interacting *via* H-bonds with both the cation and solvent. The cationic layers are also joined into the 3D structure by the longest N-H^…^O hydrogen bonding with water molecules (thin lines in Figure 12B).

In the case of crystal **2** each cation interacts *via* N-H^…^O with four others. The most important supramolecular entity is a chain of rings propagating along the *2*_1_ screw axis consisting of two types of H-bonds. The chains related by translation in [001] direction are joined by remaining N-H^…^O hydrogen bonds, and resulting in a cationic square-grid undulated layer on the plane (100) (Figure 12C). The layer exhibits the *p*1*2*_1_1 layer group symmetry. Taking into account the calculated energies for intermolecular contacts in the crystal (Figure 6; Supplementary Table S19), the chains of the *p2*_1_ rod group symmetry might be treated as LSAMs. However, they do not pack hexagonally, which in this case might result from both the additional cation-cation hydrogen bonds as well as relatively short hydrogen bonds with chloride anions. The anions are located on both sides of the layers joining them into a 3D structure (Figure 12D). The enhancement of these N-H^.^Cl interactions compared to the structure **1** might be due to the lack of solvent molecules in the crystal.

### ADMET Profile of Ornithine, DFMO and Its Analogues

DFMO exerts its action within cells, where the cell machinery of a polyamine synthesis is located. The drug, however, is very hydrophilic, and unable to cross lipid cell membranes. This is also reflected in its inability to cross the blood-brain barrier, as pointed out by Levin (Levin and Ellingson, 2018), and this difficulty in reaching the primary site of action may well reduce the efficacy of the drug, and prevent it from accessing certain target tissues where diseases amenable to its actions are manifested. To address this problem, an investigation looking at the physical characteristics of a range of analogues was conducted, to determine whether any of these could alter the properties of the molecule sufficiently to enhance its membrane permeability. The requirement for the analogue to fit into the confined space of the ligand-binding pocket of ODC, combined with the need to increase the hydrophobicity of the molecule, led to the use of the fluorine atom to substitute for hydrogen atoms in DFMO; of all the common atoms forming covalent bonds with carbon, fluorine is the smallest and most hydrophobic. Further details on properties of fluorine in the parent molecule have already been provided in the introduction. The on-line tool SwissADME and pkcsm were employed to calculate a range of parameters for each analogue, evaluating their hydrophilicity/hydrophobicity balance, and predicting the potential for passive transport across the BBB, and across lipid membranes in general. Results from this analysis are shown in Supplementary Table S23.

As can be seen, according to the criterion of the BBB permeability – one of the parameters specifically singled out by SwissADME, neither ornithine nor DFMO are able to cross membrane barriers, either in the charge or the neutral form (lines **1** – **4**). Introduction into DFMO of additional methyl groups and/or fluorine atoms (both hydrophobic moieties) resulted in an increase in lipophilicity and a decrease in hydrophilicity relative to the parent compound, and these molecules (**5**–**9**) were considered to be able to cross the BBB by SwissADME. However, examination of the interactions of DFMO with the human arginase binding site in the X-ray crystal structure 3gn0 (Ilies et al., 2011) showed that the carboxyl group in DFMO plays a key role in binding, and it was feared that conversion to the amide, as in analogues **6** and **7**, may interfere with this binding interaction. Consequently, these analogues were discounted as potential candidates for effective inhibition, and the analogue **9** was chosen for further study since this showed the greatest reduction in hydrophilicity**.**

### Molecular Docking Analysis

In order to rationalize pharmacological activity of DFMO, **1**, and its novel analogue (**9**), as potent inhibitors of human ornithine decarboxylase (h-ODC), an *in silico* enzyme−substrate docking protocol using non-commercial AutoDock Vina software (v1.1.2) (Trott and Olson, 2010) was applied. In shortening, before docking procedure was performed, the respective crystal structure of h-ODC protein was taken from the Protein Data Bank (PDB) with the 2OO0 code (Dufe et al., 2007) and used as a receptor after initial processing of the crude .pdb file by removing all non-protein molecules (i.e., conserved water molecules, cofactor, unique ligands, and ions) and addition of polar hydrogen atoms to avoid steric clashes within the model (see Experimental section for details). Docking was performed using the centered grid-box coordinates only at the A subunit since both monomers of h-ODC share the same residues and the similar conformation. In turn, ligand molecules were optimized in terms of geometry and further prepared for the docking procedure as PDBQT files by using the *Python script* “prepare_ligand4.py” that comes with AutoDock Tools vs. 1.5.6 (ADT, S3 http://mgltools.scripps.edu/) (Trott and Olson, 2010), and which included the optimization of the rotatable bonds network among the respective ligand molecule. Since DFT analyses performed by us revealed that the biological environment changes the molecular structure of DFMO in a negligible extent; we decided to use neutral (non-ionizable) DFMO-based molecules in all docking calculations.

Importantly, the reason why we decided to choose h-ODC deposited in PDB as 2OO0 and not the apo-enzyme (PDB: 1D7K; refined to 2.10 Å resolution) (Almrud et al., 2000) or its complex with small ligands (PDB: 2ON3; refined to 3.00 Å resolution) (Dufe et al., 2007) is two-fold: the crystal structure of 2OO0 protein is of the highest known resolution (1.90 Å) and it was additionally co-crystalized with 1-amino-oxy-3-aminopropane (APA; with ligand code: XAP), which is an isosteric analogue of putrescine and a significantly more potent inhibitor of h-ODC than DFMO with inhibition constants in the nM-range (*K*_i_ = 10−25 nM) (Khomutov et al., 1985; Stanek et al., 1992; Milovica et al., 2001; Seiler, 2003). The APA molecule complexed with h-ODC is bound in the substrate-binding pocket in close proximity to the h-ODC functional cofactor, namely pyridoxal-5′-phosphate (PLP). Thanks to a high-resolution X-ray structure of the complex between APA and h-ODC we could easily identify, which of the amino acids contribute to each active site of a dimeric enzyme as well as which of the residues play an important role for both PLP binding and interactions with APA. Moreover, the validation of the docking protocol was performed by re-docking of APA inhibitor into h-ODC receptor and by comparing the binding pose of the most energetically stable protein-ligand complex with the co-crystallized 2OO0 enzyme-APA inhibitor structure. As both the X-ray structure and docked structure overlapped within a similar space inside the receptor and at a root mean square deviation (*rmsd*) value of 2.11 Å our docking methodology was considered as reliable for binding simulations of the rest of the studied ligands. Only then, we set the docking calculations with the grid box centered on the substrate-binding site at the coordinates retained as close to those used for APA (see Experimental section for details). To maintain accurate simulations of the ligand−receptor interactions, docking was performed at significantly increased exhaustiveness of 48 with the search space volume >27,000 Å^3^.

**FIGURE 13:**
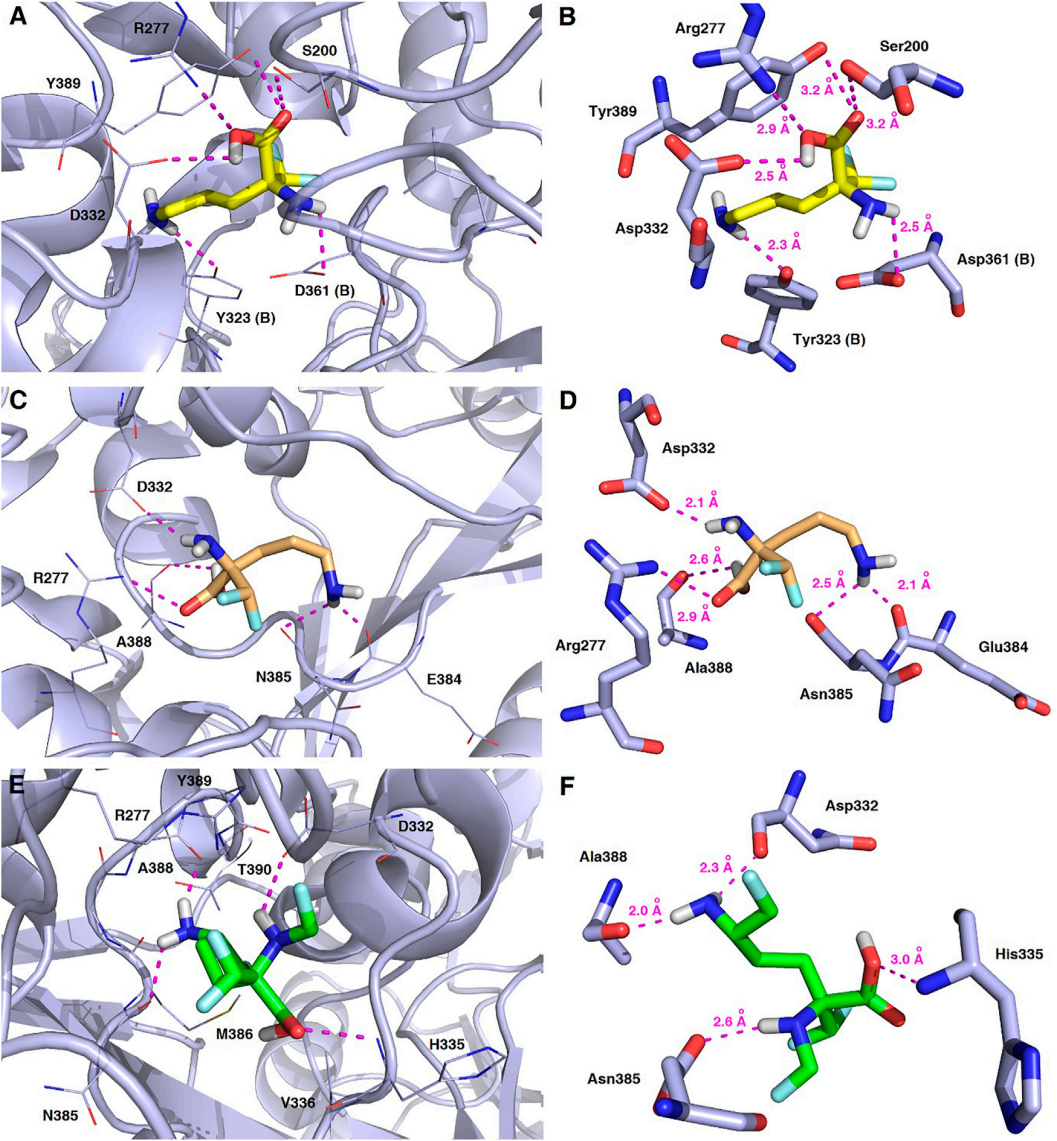
Representative binding modes of *L*-DFMO **(A,B)**, *D*-DFMO **(C,D)**, and the DFMO-analogue (**E,F**) to human ornithine decarboxylase without PLP-cofactor (h-ODC, PDB ID: 2OO0) (Dufe et al., 2007) with close contacts to residues in the active site. All six panels **(A–F)** show inhibition of h-ODC by blocking the substrate-binding site through direct interaction of the ligands with critical amino acids of the receptor in the catalytic cavity. Ligands are shown as sticks colored in yellow (*L-*DFMO), gold (*D*-DFMO) or green (DFMO-analogue), respectively. The overall enzyme structure is shown as a light blue cartoon diagram (see **A,C,E**). The most significant amino acid residues contributing to the stabilization of the ligand molecules in the complex with h-ODC by polar interactions and by CH–CH van der Waals (vdW) interactions are shown in light blue sticks (see **B,D,F**) representations. Nitrogen atoms are presented in blue, oxygen atoms in red, fluorine atoms in light blue, whereas hydrogen atoms (attached to nitrogen and/or oxygen atom of the carboxylic group) in gray. Mutual distances between the amino acid residues, and the respective ligands’ atoms are given in Ångström (see **B,D,F**). The formation of intermolecular hydrogen bonds is represented by magenta dashed lines.

As a result of docking calculations nine of the most energetically favourable binding modes being the relevant docking poses for the ligand−protein complexes for each substrate molecule (*L*-DFMO, *D*-DFMO, DFMO-analogue) and target 2OO0 protein were generated, and the results of their binding affinity energies expressed as Δ*G*_calc_ (kcal/mol) are given in Supplementary Tables S3–S8 (Supplementary Material). The visualization of the representative binding modes of *L*-DFMO, *D*-DFMO, and the DFMO-analogue to h-ODC with close contacts to residues in the active site is presented in Figure 13. The inspection of the productive pose of *L*-DFMO showed that the inhibitor possesses close polar contacts between both the amino groups and the side chains of Tyr323 (B chain) and Asp361 (B chain), while its carboxyl moiety interacted with Asp332, Arg277 and Ser200, respectively. In turn, *D*-DFMO is stabilized by strong 2.1–2.5 Å-long polar interactions between both amine moieties and the backbone atom of Asn385, Glu384 and Asp332 residues, whereas the carboxylic acid group forms H-bonds with Arg277 and/or Ala388. Complexes of h-ODC and the selected top-scoring pose of the DFMO-analogue with interacting amino acid residues revealed that primary amine group forms the hydrogen bonding with Ala388 and Asp332; secondary amine functionality interacts with Asn385, while carboxyl group is H-bounded with His335. For more details see also Supplementary Figures S14–S17 (Supplementary Material).

**FIGURE 14:**
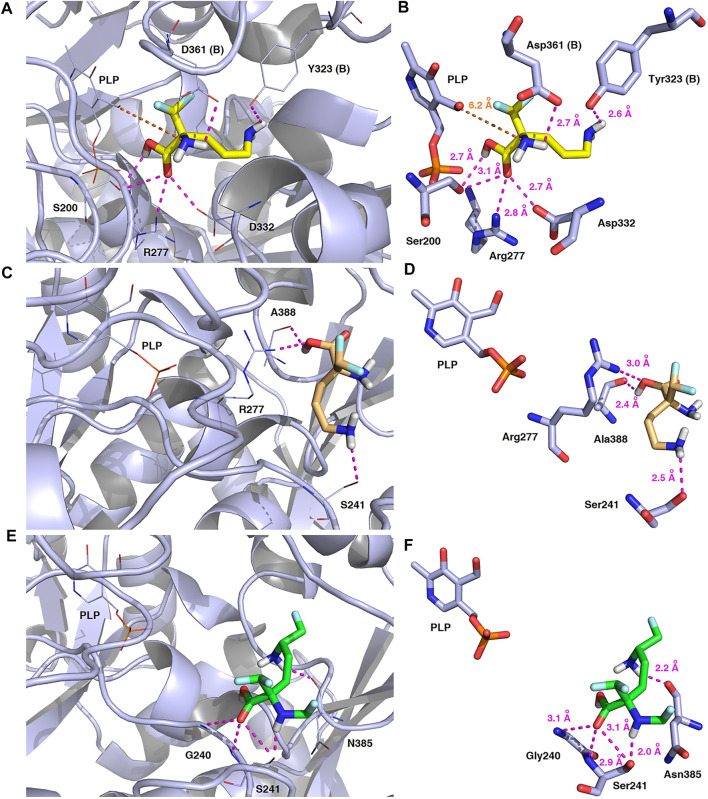
Three-dimensional model of the representative binding modes of *L*-DFMO **(A,B)**, *D*-DFMO **(C,D)**, and the DFMO-analogue **(E,F)** to human ornithine decarboxylase complexed with PLP-cofactor (h-ODC, PDB ID: 2OO0) (Dufe et al., 2007) with close contacts to residues in the active site. All six panels **(A–F)** show inhibition of h-ODC by blocking substrate-binding site through direct interaction of the ligands with critical amino acids of the receptor in catalytic cavity. Ligands are shown as sticks colored in yellow (*L*-DFMO), gold (*D*-DFMO) or green (DFMO-analogue), respectively. The overall enzyme structure is shown as a light blue cartoon diagram (see **A,C,E**). The most significant amino acid residues contributing to the stabilization of the ligand molecules in the complex with h-ODC by polar interactions and by CH–CH van der Waals (vdW) interactions are shown in light blue sticks (see **B,D,F**) representations. Nitrogen atoms are presented in blue, oxygen atoms in red, fluorine atoms in pale cyan, phosphorus atoms in orange, whereas hydrogen atoms (attached to nitrogen and/or oxygen atom of the carboxylic group) in gray. The mutual distances between the amino acid residues (and PLP-cofactor) and the respective ligands’ atoms are given in Ångström (see **B,D,F**). The formation of intermolecular hydrogen bonds is represented by magenta dashed lines. Orange dashed lines represent distances between the α-amino group of *L*-DFMO and the carbon atom of the formyl group present in the PLP-cofactor.

Validation of molecular docking of both enantiomers of the model DFMO inhibitor yielded minimum value of –4.7 kcal/mol for *L*-DFMO, with average Δ*G*_calc_ = –4.50 kcal/mol, while *D*-DFMO docking resulted in the minimum value of –4.8 kcal/mol, with average Δ*G*_calc_ = –4.54 kcal/mol. Of course, the lower the binding energy, the more suitable the binding of the inhibitor molecule to the receptor. Remarkably, *L*-DFMO underwent six hydrogen bonds which represents the highest count in our docking studies using all three inhibitors and h-ODC, respectively (Figures 13A,B). In turn, it is worth noting that the developed DFMO-analogue presents docking scores ranging from –4.8 to –5.1 kcal/mol with the average value for Δ*G*_calc_ reaching –4.91 kcal/mol (Supplementary Table S5, Supplementary Material). These results suggest as the DFMO-analogue might be slightly more potent than both DFMO enantiomers in terms of inhibition of h-ODC catalytic activity. However, when comparing only the values of the lowest energies of the formation of the ligand-receptor complexes without any critical inspection of all the generated poses and general inhibitory mechanism of h-ODC by derivatives of amino acids, one could conclude that the *D*-stereoisomer of DFMO is more efficient in inhibition of a catalytic activity of h-ODC than its *L*-DFMO counterpart. Of course, this is in strong contrary with inhibitory effects of DFMO antipodes on h-ODC established during *in vitro* tests. Considering that the inhibitor dissociation constant (*K*_D_) values for the formation of h-ODC–DFMO complexes are at 1.3 FM0.3 μM conc. for *L*-DFMO and 28.3 DF3.4 μM conc. for *D*-DFMO (Qu et al., 2003), the inhibitory potency toward h-ODC is ca. 20 times greater for *L*-DFMO when compared with *D*-DFMO. Therefore, further evaluation of the binding poses was performed to select only productive conformations of the ligand molecules, which can form covalent coenzyme-substrate adducts that cease the catalytic activity of PLP-dependent ODC. In this regard, the second series of docking calculations were performed with h-ODC containing the PLP-cofactor in the catalytic cavity to include possibility of the formation of the phosphopyridoxyl-amino acids intermediate with studied ligands according to one of the postulated mechanisms of ODC-inhibition by amino acid-like substrates (Wu et al., 2007; Correa-Basurto et al., 2008; Preeti et al., 2013). Details of the contacts between the halogenated compounds and homodimeric form of human ornithine decarboxylase in the most energetically stable binding pose including the hydrogen-bonding network connecting the studied inhibitors and the h-ODC active site are depicted in Figure 14.

**FIGURE 15:**
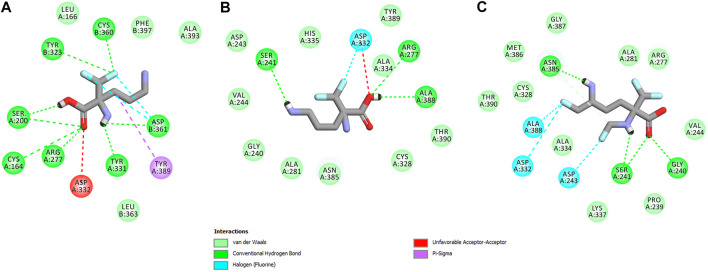
Two dimensional visualization of the h-ODC (PDB ID: 2OO0) (Dufe et al., 2007) binding interfaces as computed by BIOVIA Discovery Studio Visualizer 20.1.0.19295 software for top-scoring poses of the ligand molecules *L*-DFMO **(A)**, *D*-DFMO **(B)**, and DFMO-analogue **(C)**, respectively. Polar contacts between receptor-ligand including hydrogen bonds (green), unfavorable hydrogen bond acceptor–acceptor (AA) clashes (red), halogen bonds (cyan), and π–σ interactions (purple) are represented by dashed lines. Intermolecular Van der Waals forces are displayed in light-green spoked arcs respectively.

In this case, docking results revealed completely different binding affinity for both DFMO enantiomers in favor of *L*-DFMO, which obtained the lowest energies of the respective ligand-receptor complex formation for h-ODC [ranging from –4.7 to –5.4 kcal/mol with average values of Δ*G*_calc_ = –4.96 kcal/mol (Supplementary Table S6 Supplementary Material)]. These results are also impressive in terms of binding affinity when compared to nanomolar APA inhibitor, which interactions inside the substrate-binding cleft of h-ODC complexed with PLP revealed binding energy of only −3.8 kcal/mol for the best mode inspected (Supplementary Table S2, Supplementary Material). What is even more important, hypothetical formation of a Schiff base of PLP with the *α*-amino group of the fluorinated substrates might be achievable only in the case of *L*-DFMO located significantly closer to the cofactor (6.2 Å) when compared with two other studied inhibitors (>14.3 Å). Moreover, *L*-DFMO was stabilized by multiply strong H-bonds of 2.6–3.1 Å distance formed between the carboxyl moiety of the ligand and the hydrogen atom of the –OH group of the Ser200 residue from one side and the guanidine group of Arg277 as well as –COOH of Asp322 from the other side. However, the interactions between Asp322 and *L*-DFMO are rather unfavorable since acceptor-acceptor clashes are observed in this case (Figure 15). In turn, the α-amino group of *L*-DFMO forms a 2.7 Å-long hydrogen bond with the carboxylic acid group of the Asp361 (B) side chain and/or Tyr331, whereas the terminal *δ*-NH_2_ group interacts with the oxygen atom of the hydroxyl moiety of Tyr323 (B) in the close distance of 2.6 Å. In addition, single π–σ contact between methylene group of the alkyl chain and Tyr389 was detected. Interestingly, only in the case of *L*-DFMO two extra interfacial residues, namely Asp361 (B), Tyr323 (B), and Cys360 (B), that belong to the B subunit of the dimeric enzyme, participate in the stabilization of the ligand molecule in the complex with h-ODC (Figures 14A,B). In the case of *L*-DFMO additional halogen bonding interactions between oxygen atom of carboxylic group belonging to Asp361 (B) residue and fluorine atoms of *α*-difluoromethyl group was detected (Figure 15).

In sharp contrast, although *D*-DFMO essentially occupies the position of the substrate in the active site interacting with the crucial Arg277 residue, which is fundamental for the h-ODC activity and overall catalysis, it is positioned far away from the cofactor location and the formation of an aldimine intermediate with PLP is obviously not possible (Figures 14C,D). Other potential polar interactions between the less favored *D*-stereoisomer and h-ODC within a radius of 2.4–2.5 Å involve Ser241 and Ala388. In the case of *D*-DFMO halogen bonding was detected between *α*-difluoromethyl group of the ligand and Asp332 residue (Figure 15). More specifically, the optimized binding mode of the bulky DFMO-analogue revealed that the ligand is also pulled far away from the PLP location, thus accommodating in the larger hydrophilic cavity surrounded by the subsequent aliphatic amino acids: Gly240, Ser241, Lys337, Val244, Ala281, Ala334, and Asn385 (Figures 14E,F). Furthermore, docking investigations revealed that additional binding interactions *via* fluoromethyl group present in DFMO-analogue was observed as halogen bonds between F atom and Asp243 was observed in this case (Figure 15). It turned out that such functionalization of the *α*-amine group in DFMO-analogue has significantly changed the way in which this ligand is accommodated in the h-ODC active site. In contrary to *L*-DFMO molecule its analogue lost 2.7 Å-long H-bonding with Asp361(B) and thus is pulled far away from the PLP functional cofactor. Instead of this DFMO-analogue gained a closer (and thus stronger) 2.0 Å-long hydrogen bond interactions with hydroxyl moiety of the Ser241 residue located in a different region of the binding pocket and few other halogen bonds with Ala388 and/or Asp332 (Figure 15). The main lesson to be drawn from the *in silico* data presented in this paper is that closely related compounds sharing DFMO scaffold can bind to the active site of h-ODC in substantially different manners as a consequence of subtle structural alterations in terms of fluorine atom redistribution. In this case the binding potency cannot be correlated in a straightforward manner just to the number and type of polar interactions observed, but other interactions including, i.e., van der Waals forces or steric clashes in protein structure, should also be included. Probably it is also the restricted size of the pocket nearby the PLP cofactor that forces the DFMO-analogue to adopt different conformation which is unable to enter deeply into this site. Therefore, the only reliable tool for rationalization of the predictions made by computational modeling is the experimental approach including enzymatic activity assay. Notable, the DFMO-analogue furnished promising results in terms of consistency of the formed complexes with h-ODC by spending vast majority of poses within the selected binding cavity, while both DFMO enantiomers occupied the enzyme active site only with maximum 2–4 poses from the best mode according to the calculated *rmsd*-values, respectively (Supplementary Tables S6–S8, Supplementary Material). Since *rmsd* is very important parameter that imparts the information of the overall stability of the protein-ligand complex in terms of calculating standard deviation from the initial structure, it is worth to note that for DFMO-analogue bound to target protein, the *rmsd*-value for the best pose was found to be 1.58 Å with the average *rmsd* of 3.10 Å suggesting its stability. Of course, to estimate which of the inhibitor forms the most stable complexes, the best arrangements of the ligands along with spatially optimized binding cavities of the enzyme molecule should be further subjected to a molecular dynamics simulation (Bitencourt-Ferreira and Filgueira de Azevedo, 2019; Bitencourt-Ferreira et al., 2020; Bitencourt-Ferreira et al., 2021). Moreover, to confirm if binding of the DFMO-analogue has potential physiological relevance, *in vitro* inhibitory evaluations with isolated enzyme must be performed. For more details on the formation of potential h-ODC-DFMO-analogue complexes see Supplementary Figures S15–S19 appended in Supplementary Material.

## Conclusion

In summary, in the face of past, current and possible future global pandemics, further studies on the advancement of an effective and safe therapy is needed. In this featured article, the latest scientific findings related to the polyamine pathway as a target and promising avenue for the antiviral therapy are shortly overviewed. Here, we present the first thorough exploration of the supramolecular system of popular, broad-spectrum, antiviral and anticancer drug DFMO (**1**), the best known inhibitor of ornithine decarboxylase (ODC), in relation to the supermolecule of ornithine (**2**). In spite of the investigation of **1** for almost half a century, its supramolecular aspects have not been known so far. Optimized parameters, using the DFT method, are in good agreement with the experimental data. The compound **1** features an interesting supramolecular well-organized self-assembly governed by classical and non-classical, strong and weak hydrogen bonding interactions that are evaluated in detail by means of Hirshfeld surface calculations. The analysis indicated that H…H (38%) and O…H/H…O (26%), from F^…^H/H^…^F (20%), Cl^…^H/H^…^Cl (11%), F^…^O/O^…^F (2%), C^.^H/H^…^C (above 1%) interactions are important contributors to the crystal packing. Consequently, weak inter-contacts have relevance in the control of the supramolecular network of DFMO molecules, both at the first and second levels of the supramolecular architecture. The difference between the electrostatic and dispersion forces in the stabilization of molecular packing is insignificant. In the case of supermolecule **2**, the classical electrostatic energy framework is dominant. The molecular electrostatic potential mapped on the Hirshfeld surfaces visualized electrostatic complementarities of H-bonding donors and acceptors involved in the formation of supramolecular synthons. The same synthons were found in the bio-complexes. Notably, DFMO is a good multipurpose inhibitor in diverse diseases, but with pharmacokinetic shortcomings, especially when large doses are required for the treatment. Therefore, the necessity of therapeutical improvements, another important issue of this work, has been pointed out. In this context, the design of the series of DFMO analogues by an introduction of an additional methyl group and fluorine atoms was carried out. In consequence, an increase in liphophilicity and BBB permeability and a decrease in hydrophilicity were observed. The analogue with the best pharmacokinetic profile was selected for docking studies. Two strategies of molecular docking were compared depending on potential inhibition mechanisms of h-ODC by fluorinated amino acid derivatives. One employed apo-h-ODC as the receptor, and the other kept the cofactor [pyridoxal-5′-phosphate (PLP)] in the protein for docking purposes. From the series of ligands docked into a h-ODC protein containing the functional PLP-cofactor, *L*-DFMO was found to possess the lowest estimated energy of binding affinity in terms of the docking score with the value of ΔG_calc_ = –5.40 kcal/mol. Moreover, docking yielded very low energy of binding (up to ΔG_calc_ = –5.10 kcal/mol) for *D*-DFMO as well as the DFMO-analogue despite the far vicinity of PLP to which the closest distance is 14.3 Å, which is rather high for any direct interaction to take place. In turn, due to the consistent results obtained from *in silico* calculations with respect to stabilization of the h-ODC–DFMO-analogue complex mostly by several hydrogen and halogen bonds, there is a strong indication that the developed compound may reveal promising inhibitory potency toward h-ODC and thus can be therapeutically useful as an anticancer/antiviral agent. Molecular docking simulations also revealed that even subtle changes in the structure of DFMO-like compounds play an important role in the orientation of each individual ligand inside the h-ODC binding pocket, and thus may significantly modulate their inhibitory potency *in vitro*. Nevertheless, this hypothesis will have to be tested *via in vitro* kinetics studies since docking simulations do not consider several biological factors such as the presence of explicit solvent and ionic strength. Further, advanced studies are in progress and will be published in the near future.

To sum up, these studies provide future drug designers with insight into orientation of DFMO and its analogues in the active site of ODC1, and consequently facilitate the design of DFMO mimic.

## Data Availability

The datasets presented in this study can be found in online repositories. The names of the repository/repositories and accession number(s) can be found in the article/Supplementary Material.

## Abbreviations

ADMET, absorption, distribution, metabolism, excretion, toxicity; CPCM, conductor-like polarizable continuum model; CSD, Cambridge structure database; DFMO, difluoromethylornithine; DFT, density functional theory; DNA, deoxyribonucleic acid; ER, enrichment ratio; ESP, electrostatic potential; FDA, food and drug administration; FP, fingerprint plot; HS, Hirshfeld surface; LSAM, long range aufbau module; MERS-CoV, Middle East respiratory syndrome coronavirus; ODC, ornithine decarboxylase; RCSB PDB, research collaboratory for structural bioinformatics protein data bank; RNA, ribonucleic acid; SARS-CoV-2, severe acute respiratory syndrome coronavirus
