# Supplementary material for: The First Insight Into the Supramolecular System of D,L-α-Difluoromethylornithine: A New Antiviral Perspective
Source: Front Chem. 2021 May 13;9:679776. doi: 10.3389/fchem.2021.679776 (PMC8155678; doi:10.3389/fchem.2021.679776)
Supplement: Supplementary file 1 [file DataSheet1.PDF]

Supplementary Material

The First Insight Into the Supramolecular System of *D,L*-α-Difluoromethylornithine: A New Antiviral Perspective

Joanna Bojarska[1*], Roger New[2], Paweł Borowiecki[3], Milan Remko[4], Martin Breza[5], Izabela D. Madura[6], Andrzej Fruziński[1], Anna Pietrzak[1] and Wojciech M. Wolf[1]

[1]Technical University of Lodz, Chemistry Department, Institute of Ecological and Inorganic Chemistry, Żeromskiego 116, 90-924 Lodz, Poland

[2]Faculty of Science & Technology, Middlesex University, The Burroughs, London NW4 4BT, United Kingdom

[3]Faculty of Chemistry, Warsaw University of Technology, Department of Drugs Technology and Biotechnology, Laboratory of Biocatalysis and Biotransformation, Koszykowa St. 75, 00-662 Warsaw, Poland

[4]Remedika, Sustekova 1, 85104, Bratislava, Slovakia

[5]Department of Physical Chemistry, Slovak Technical University, Radlinskeho 9, SK-81237 Bratislava, Slovakia

[6]Faculty of Chemistry, Warsaw University of Technology, Noakowskiego 3, 00-664,Warsaw, Poland

SUPPLEMENTARY FIGURES AND TABLES

FIGURES

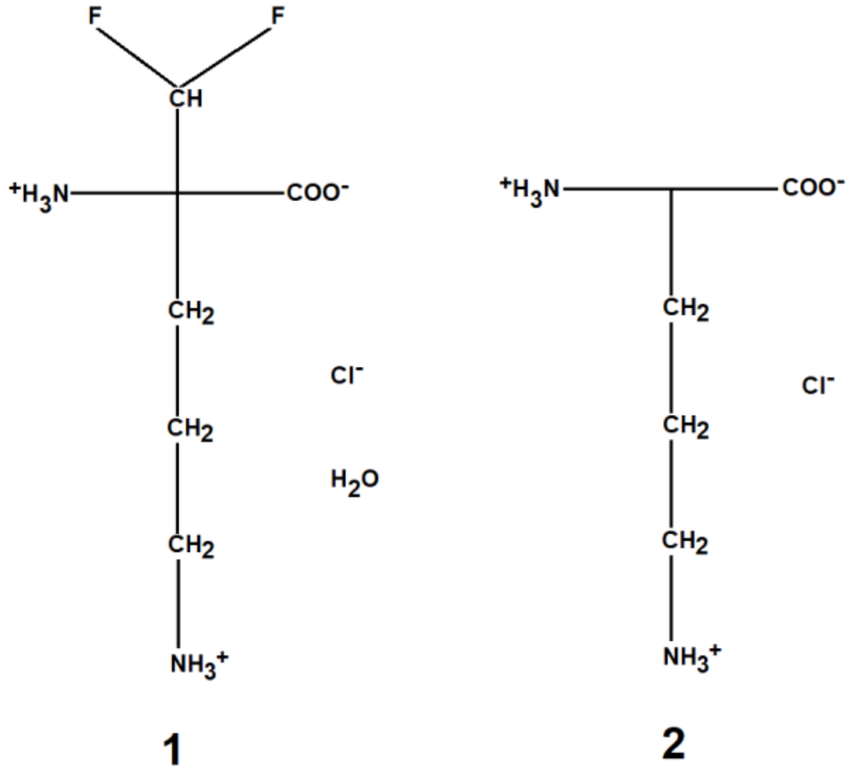

FIGURE S1 Chemical structures of DFMO, called **1** and ornithine, **2** (Ditrich et al., 2007).

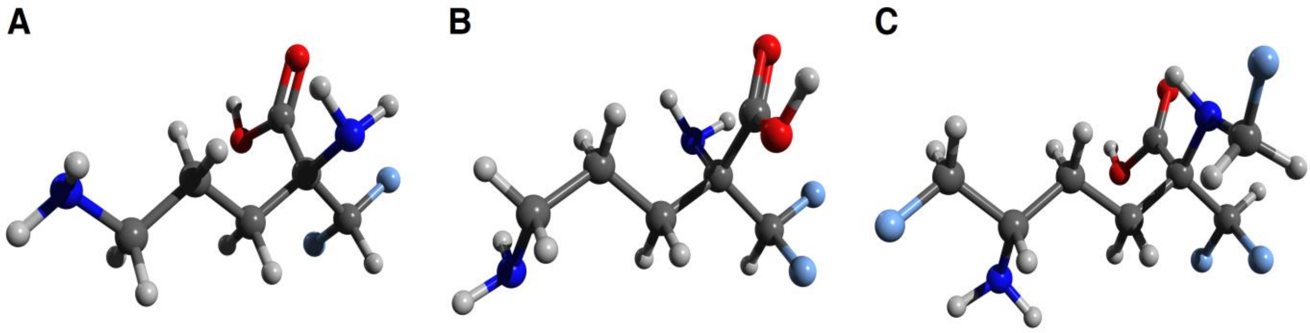

FIGURE S2 The geometries of *L*-DFMO (**A**), *D*-DFMO (**B**), and the DFMO-analogue (**C**) optimized in Avogadro – Version 1.2.0. The figures were prepared by rendering them by using molecular visualization

software, POV-Ray – Version 3.7.0. Nitrogen atoms are presented in blue, oxygen atoms in red, fluorine atoms in light blue, whereas hydrogen atoms are expressed as light-grey balls.

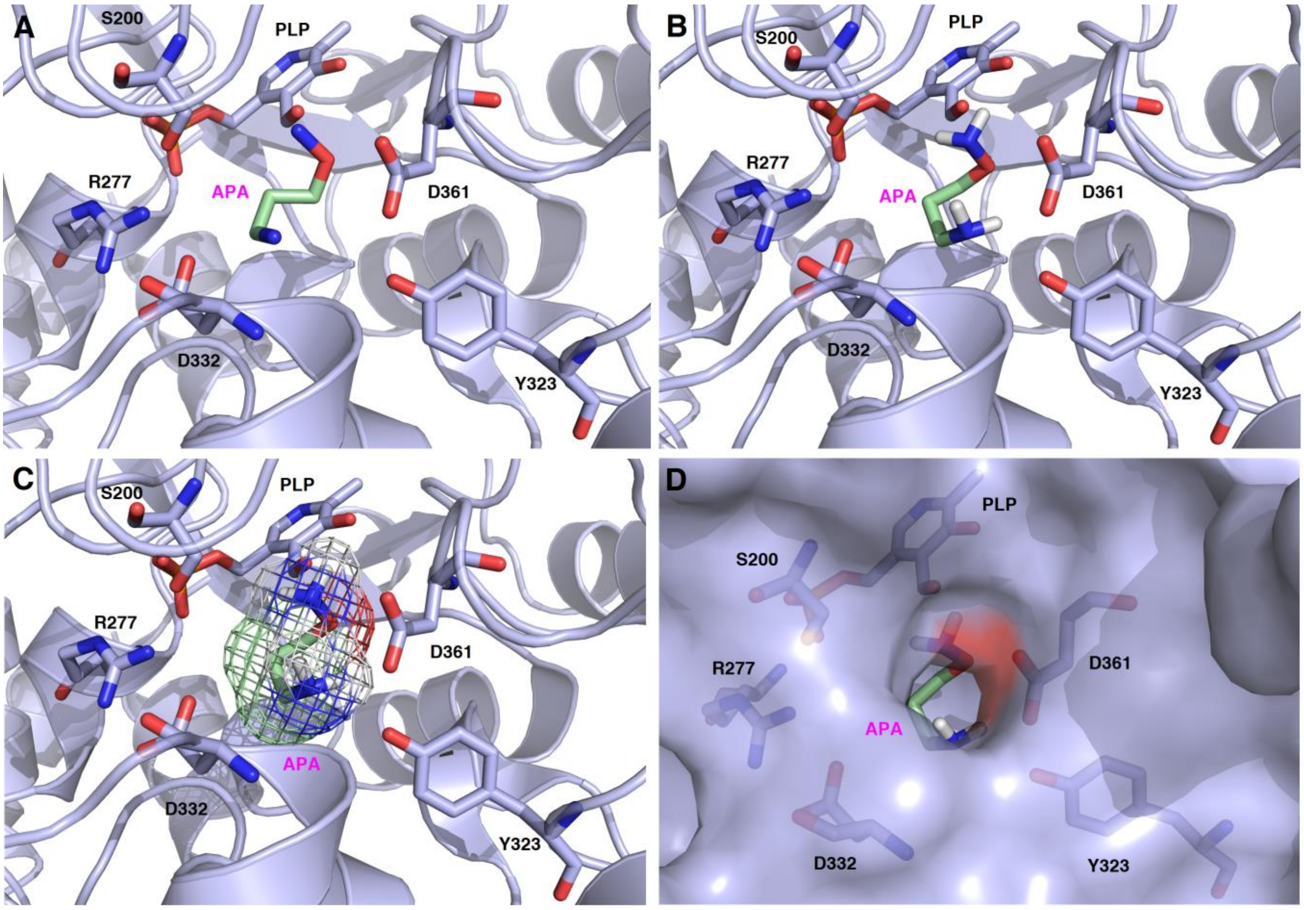

FIGURE S3 Structures of h-ODC (**A**) co-crystallized with 1-amino-oxy-3-aminopropane (APA; with ligand code: XAP) (PDB access code: 2OO0) and (**B**) with docked APA. Both protein structures are shown in the same orientation. The overall enzyme structure (protein target) is shown as a light blue cartoon diagram (see **A**, **B**, **C**) or as the surface diagram (**D**). The most significant amino acid residues contributing to the stabilization of the ligand molecules are shown in light blue or sticks (see **A**–**D**) representations. Nitrogen atoms are presented in blue, oxygen atoms in red, fluorine atoms in pale cyan, whereas hydrogen atoms in grey. The docked pose of APA overlapped with the pose in the X-ray crystal structure (PDB 2OO0) at an average root mean square deviation (rmsd) value of 2.11 Å. The APA ligand is denoted in light-green stick representation, whereas surrounding residues are shown as light-blue sticks. The F_{obs}–F_{calc} density (omit map) of docked APA is contoured at 3σ level and shown as multi-colored mesh (**C**), while the (**D**) overview shows the surface diagram of h-ODC with docked APA ligand and surrounding residues shown with stick models. Interactions of APA inside the binding pocket of h-ODC revealed binding energy of −3.8 kcal/mol for the best mode. Concluding, it was found that the conformation of APA ligand (B–D) docked into the active site was almost identical to the original ligand (A), suggesting the reliability of this docking protocol.

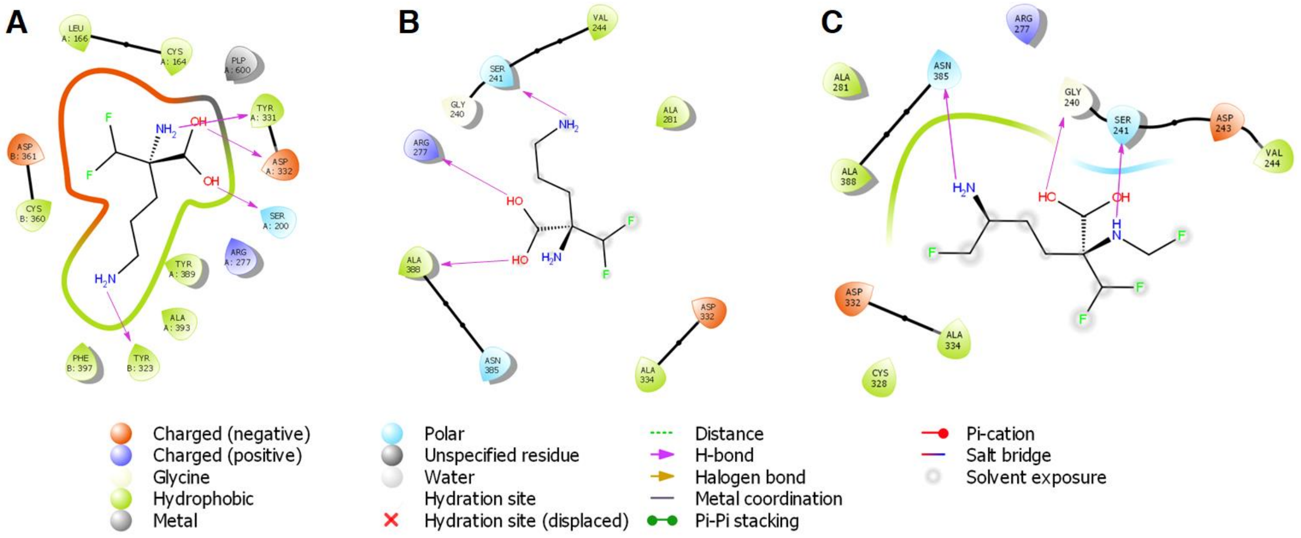

FIGURE S4 Complexes of h-ODC (PDB ID: 2OO0) (Dufe et al., 2007) with PLP-cofactor and selected top-scoring poses of *L*-DFMO (**A**), *D*-DFMO (**B**), and DFMO-analogue (**C**) with interacting amino acid residues. The 2D protein-ligand interactions maps were analyzed by using freeware for academia Maestro Version 12.6.144, MMshare Version 5.2.144, Release 2020-4, Platform Windows-x64 for academics of the Schrödinger suite.

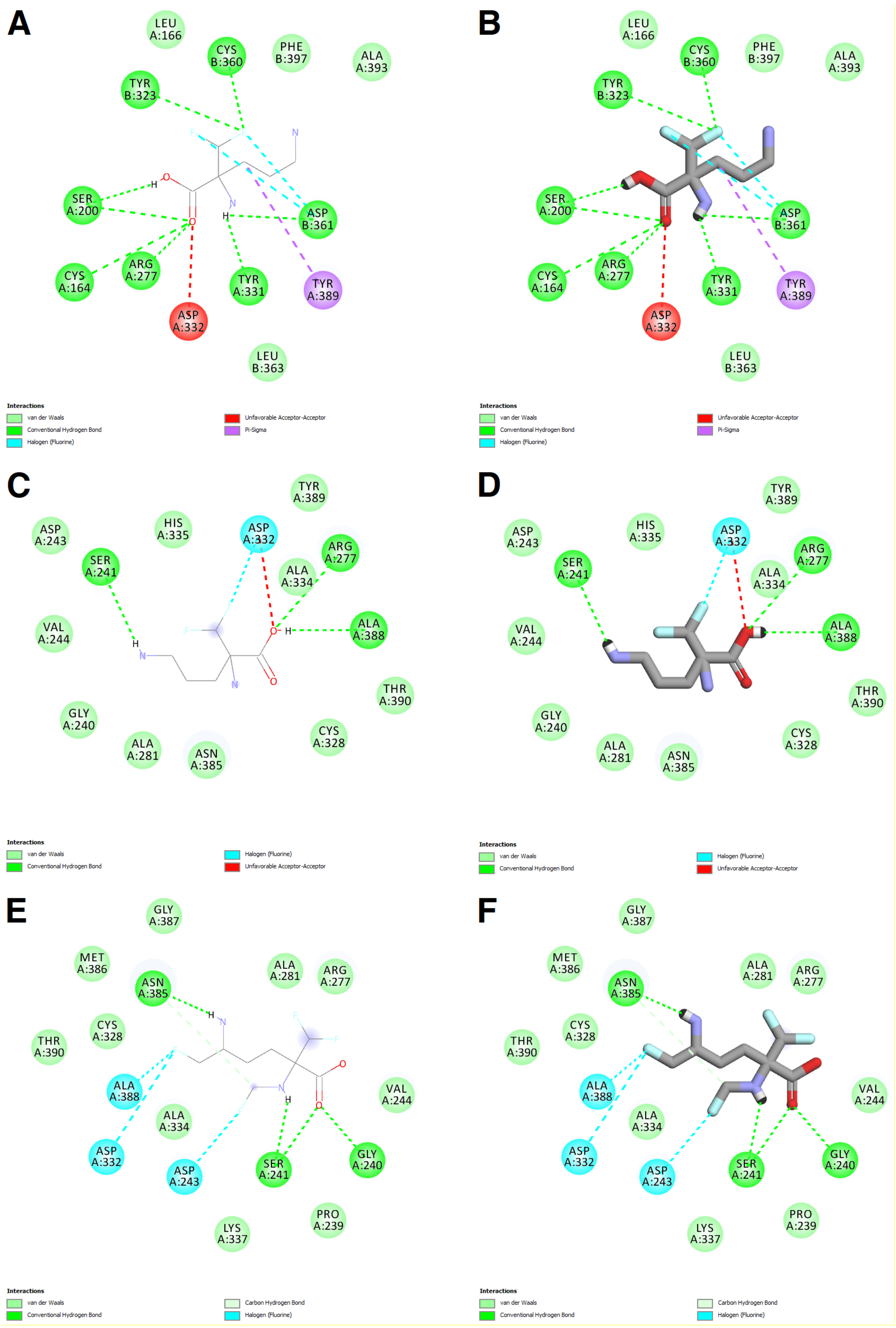
A
LEU A:166
CYS B:360
PHE B:397
ALA A:393
TYR B:323
SER A:200
ASP B:361
CYS A:164
ARG A:277
TYR A:331
TYR A:389
ASP A:332
LEU B:363
Interactions
van der Waals
Conventional Hydrogen Bond
Halogen (Fluorine)
Unfavorable Acceptor-Acceptor
Pi-Sigma
B
LEU A:166
CYS B:360
PHE B:397
ALA A:393
TYR B:323
SER A:200
ASP B:361
CYS A:164
ARG A:277
TYR A:331
TYR A:389
ASP A:332
LEU B:363
Interactions
van der Waals
Conventional Hydrogen Bond
Halogen (Fluorine)
Unfavorable Acceptor-Acceptor
Pi-Sigma
C
TYR A:389
ASP A:243
HIS A:335
ASP A:332
ARG A:277
SER A:241
ALA A:334
VAL A:244
ALA A:388
THR A:390
GLY A:240
CYS A:328
ALA A:281
ASN A:385
Interactions
van der Waals
Conventional Hydrogen Bond
Halogen (Fluorine)
Unfavorable Acceptor-Acceptor
D
TYR A:389
ASP A:243
HIS A:335
ASP A:332
ARG A:277
SER A:241
ALA A:334
VAL A:244
ALA A:388
THR A:390
GLY A:240
CYS A:328
ALA A:281
ASN A:385
Interactions
van der Waals
Conventional Hydrogen Bond
Halogen (Fluorine)
Unfavorable Acceptor-Acceptor
E
GLY A:387
MET A:386
ASN A:385
ALA A:281
ARG A:277
CYS A:328
THR A:390
ALA A:388
VAL A:244
ALA A:334
ASP A:332
ASP A:243
SER A:241
GLY A:240
PRO A:239
LYS A:337
Interactions
van der Waals
Conventional Hydrogen Bond
Carbon Hydrogen Bond
Halogen (Fluorine)
F
GLY A:387
MET A:386
ASN A:385
ALA A:281
ARG A:277
CYS A:328
THR A:390
ALA A:388
VAL A:244
ALA A:334
ASP A:332
ASP A:243
SER A:241
GLY A:240
PRO A:239
LYS A:337
Interactions
van der Waals
Conventional Hydrogen Bond
Carbon Hydrogen Bond
Halogen (Fluorine)

FIGURE S5 Complexes of h-ODC (PDB ID: 2OO0) (Dufe et al., 2007) with PLP-cofactor and selected top-scoring poses of *L*-DFMO (**A**–**B**), *D*-DFMO (**C**–**D**), and DFMO-analogue (**E**–**F**) with interacting amino acid residues. The 2D protein-ligand interactions maps were generated by using freeware for academia BIOVIA Discovery Studio Visualizer 20.1.0.19295 software (Dassault Systèmes Biovia Corp.; https://www.3ds.com).

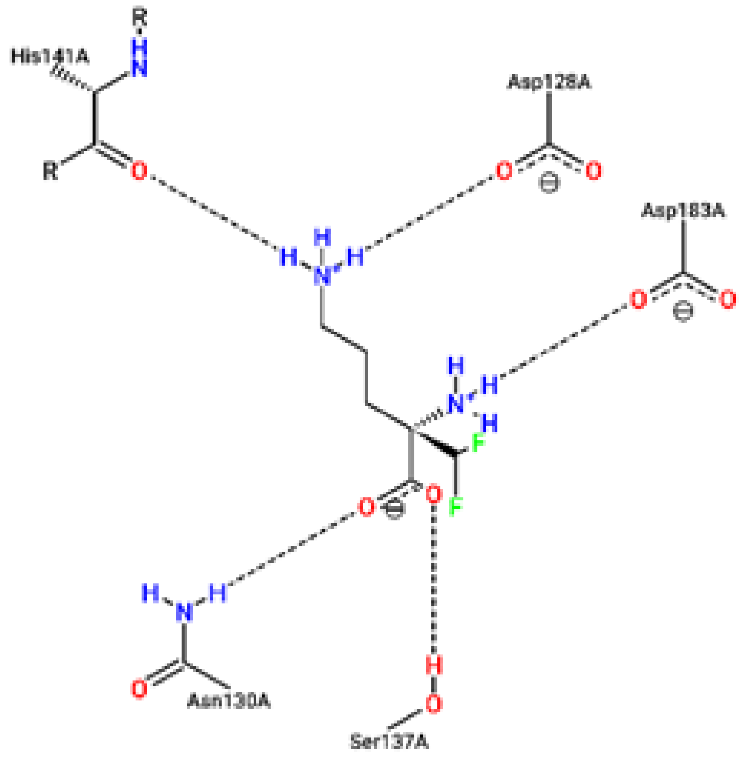

FIGURE S6 Perspective diagram of the complex of DFMO in the bound state at the human arginase I generated by PoseView (PDB code: 3gn0 (Ilies et al., 2011; Wallace et al., 1995; Laskowski & Swindells, 2011).

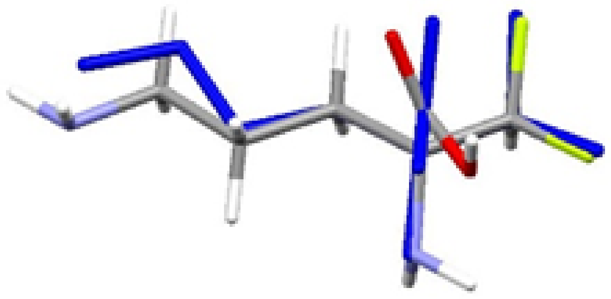

FIGURE S7 Molecular superimposition of the molecular structure of DFMO 3gn0.pdb (blue) and of the thermodynamically stable RM06/6-311++G(d,p) optimized neutral DFMO.

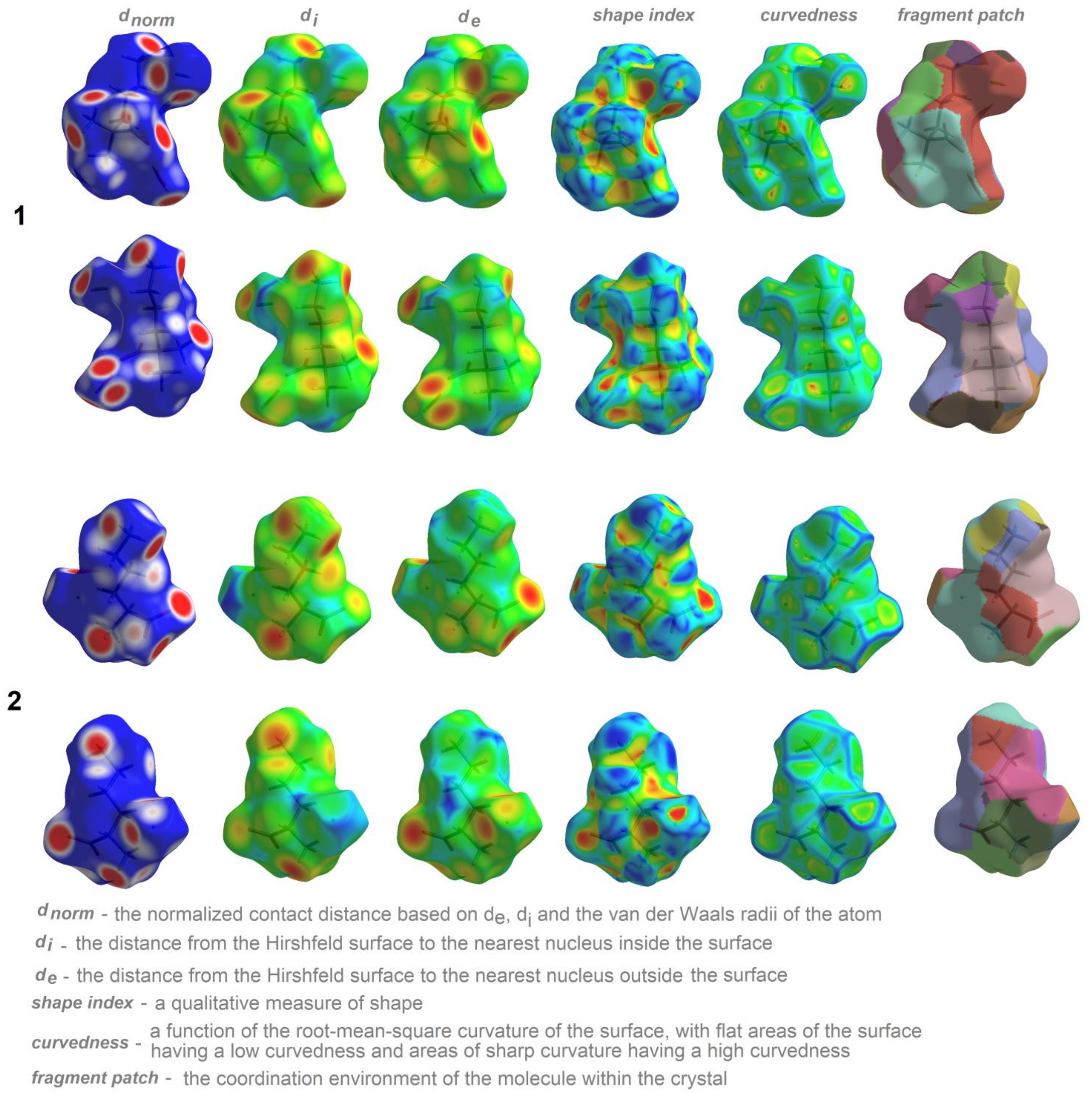

FIGURE S8 HSs mapped of d_i, d_e, d_{norm}, *curvedness, shape index*, and *fragment patch* in analysed crystals **1** and **2**.

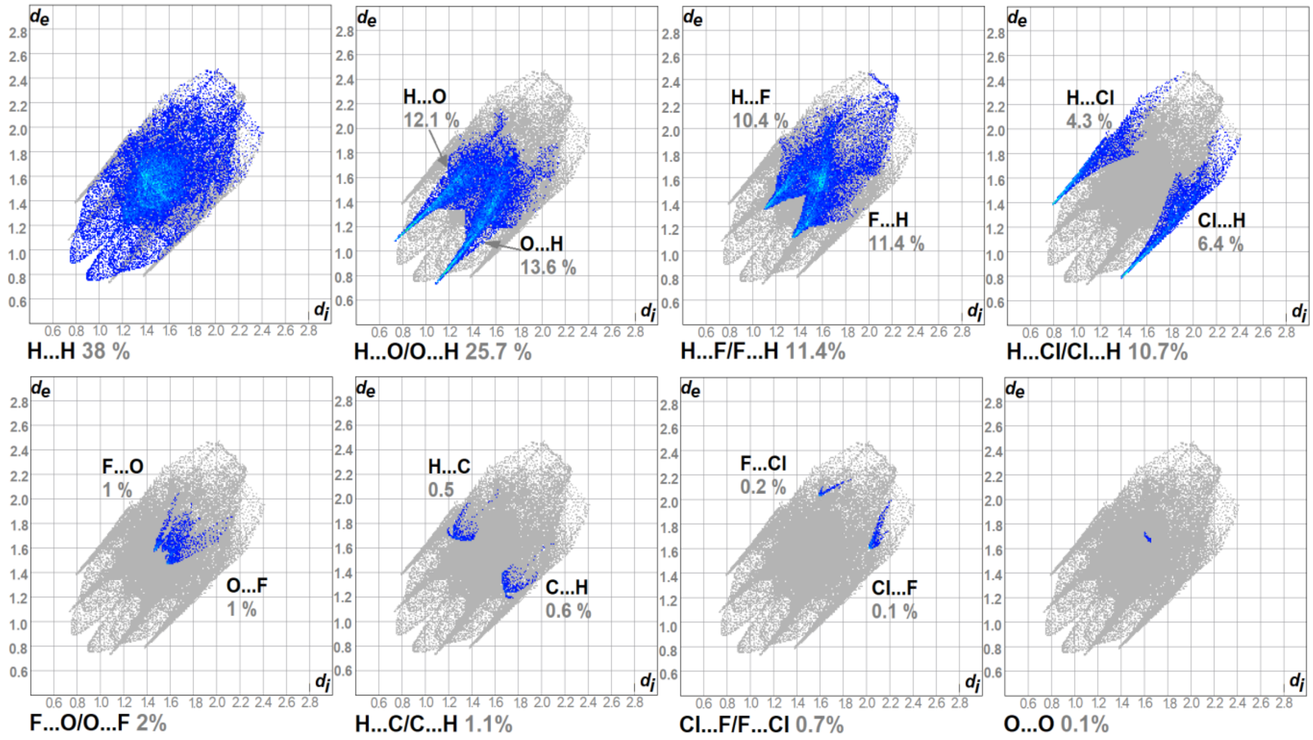

FIGURE S9 Decomposed 2D fingerprint plots representating close interactions in **1**.

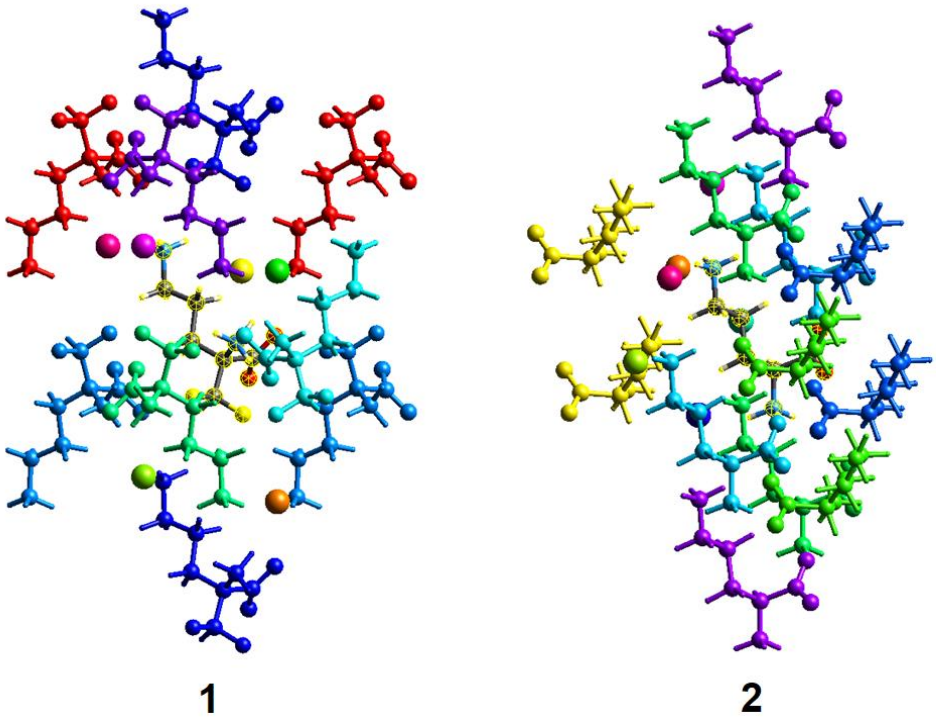

FIGURE S10 Molecular interactions between the central molecule (gray) and the neighboring molecules present in a radius 3.8 Ǻ. (For interpretation of the references to color in this figure, the reader is referred to the web version of this article.)

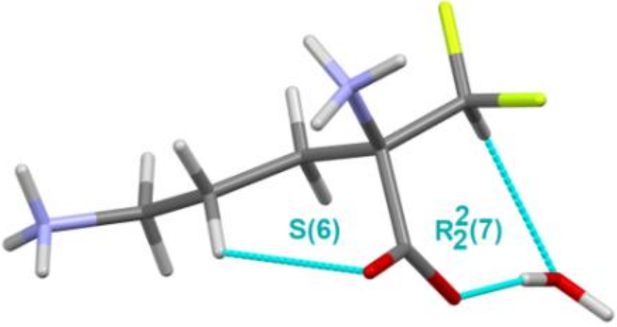

FIGURE S11 Synthonic interactions as amplification of the molecular structure of **1**.

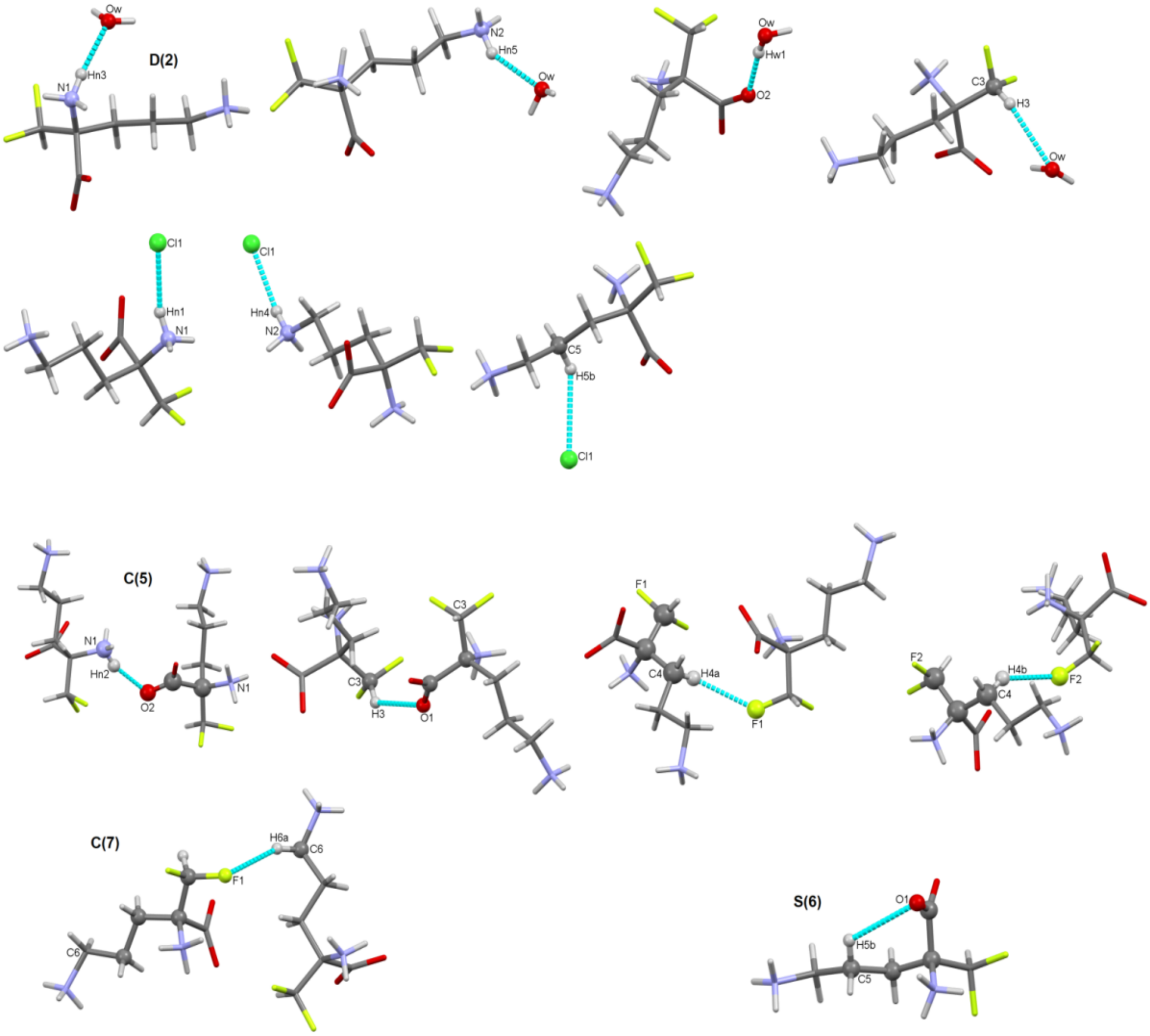

FIGURE S12 Synthons at the first level of supramolecular architecture in **1**.

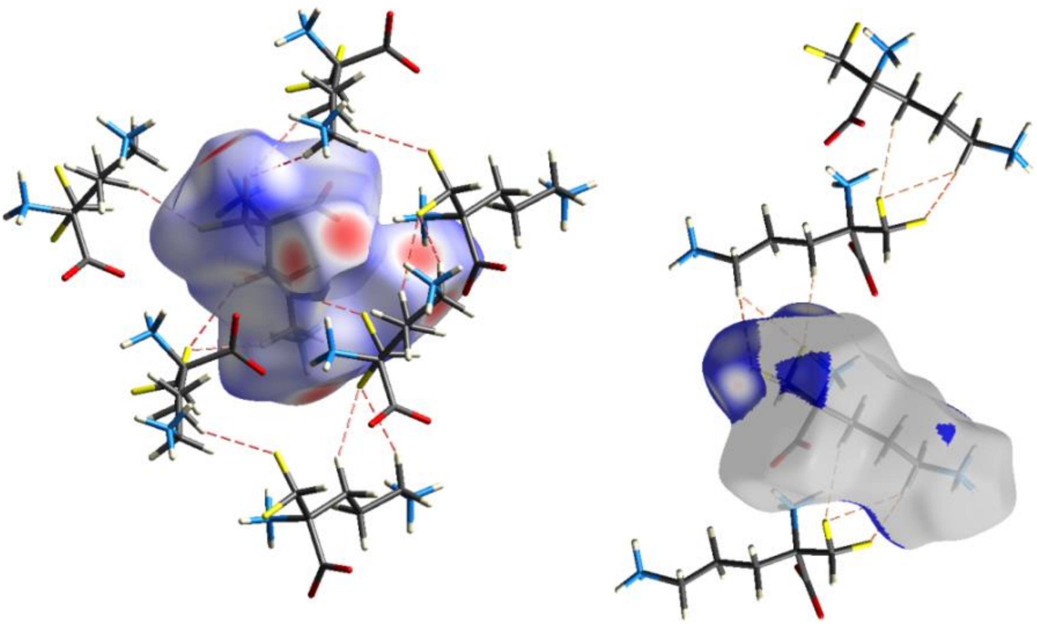

FIGURE S13 C–H···F interactions involved in the formation of supramolecular fluorine-based synthons on the 3D HS mapped over d_{norm} property in **1**.

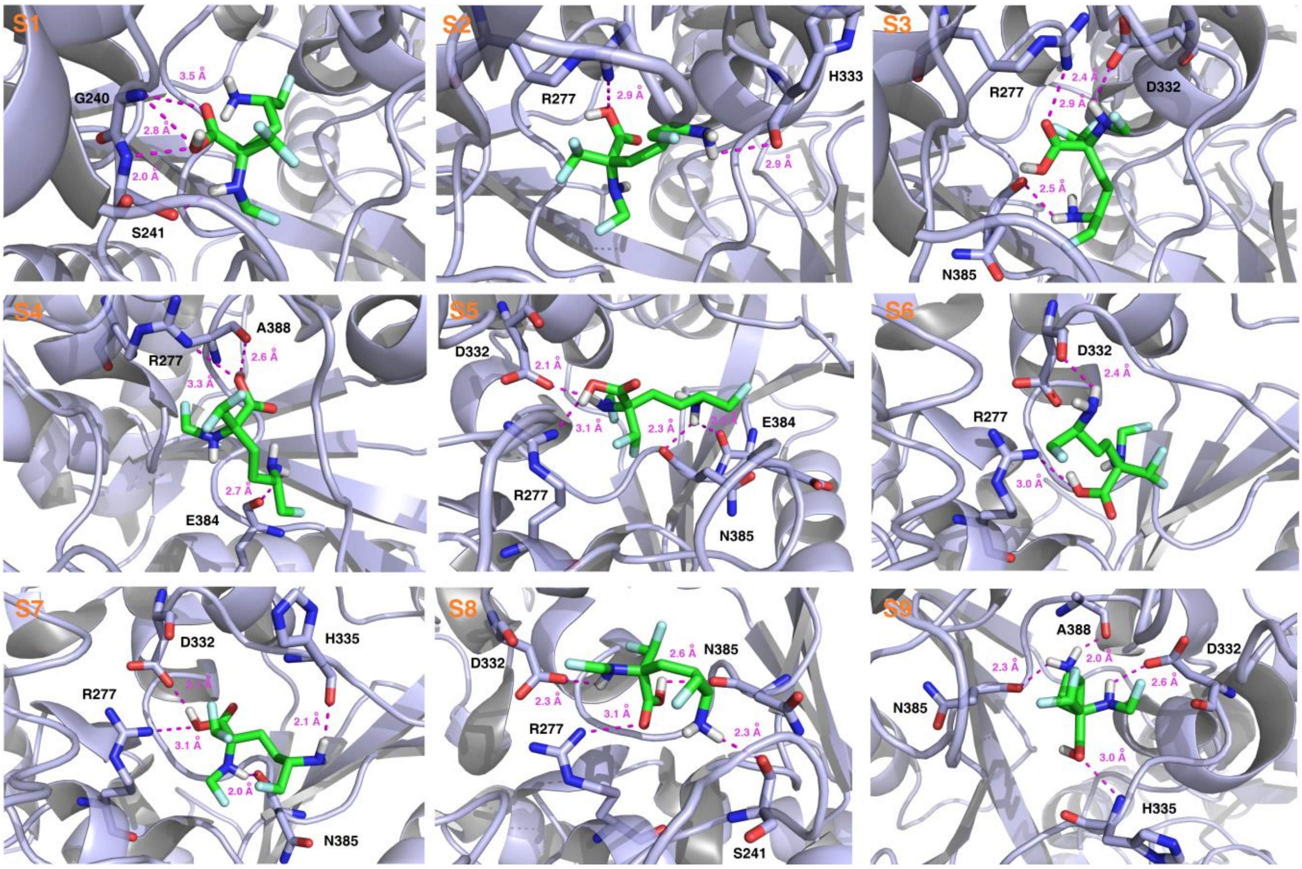

FIGURE S14 Binding mode proposals (**S1**–**S9**) for the complex of h-ODC (PDB ID: 2OO0, shown as light blue-colored cartoon diagram) (Dufe et al., 2007) without PLP-cofactor and DFMO-analogue (green sticks). The pose S1 represents the lowest value of ΔG_{calc} (kcal/mol), which means that its ligand-binding affinity to receptor (h-ODC active-site) is the highest, and in contrary, the S9 mode represent the lowest ligand-binding affinity. The formation of intermolecular hydrogen bonds is represented by magenta dashed lines. The mutual distances between the critical for catalytic active amino acid residues and the respective atoms of the DFMO-analogue are given in Ångström. Nitrogen atoms are presented in blue, oxygen atoms in red, fluorine atoms in pale cyan, whereas hydrogen atoms (attached to nitrogen and/or oxygen atom of the carboxylic group) in grey.

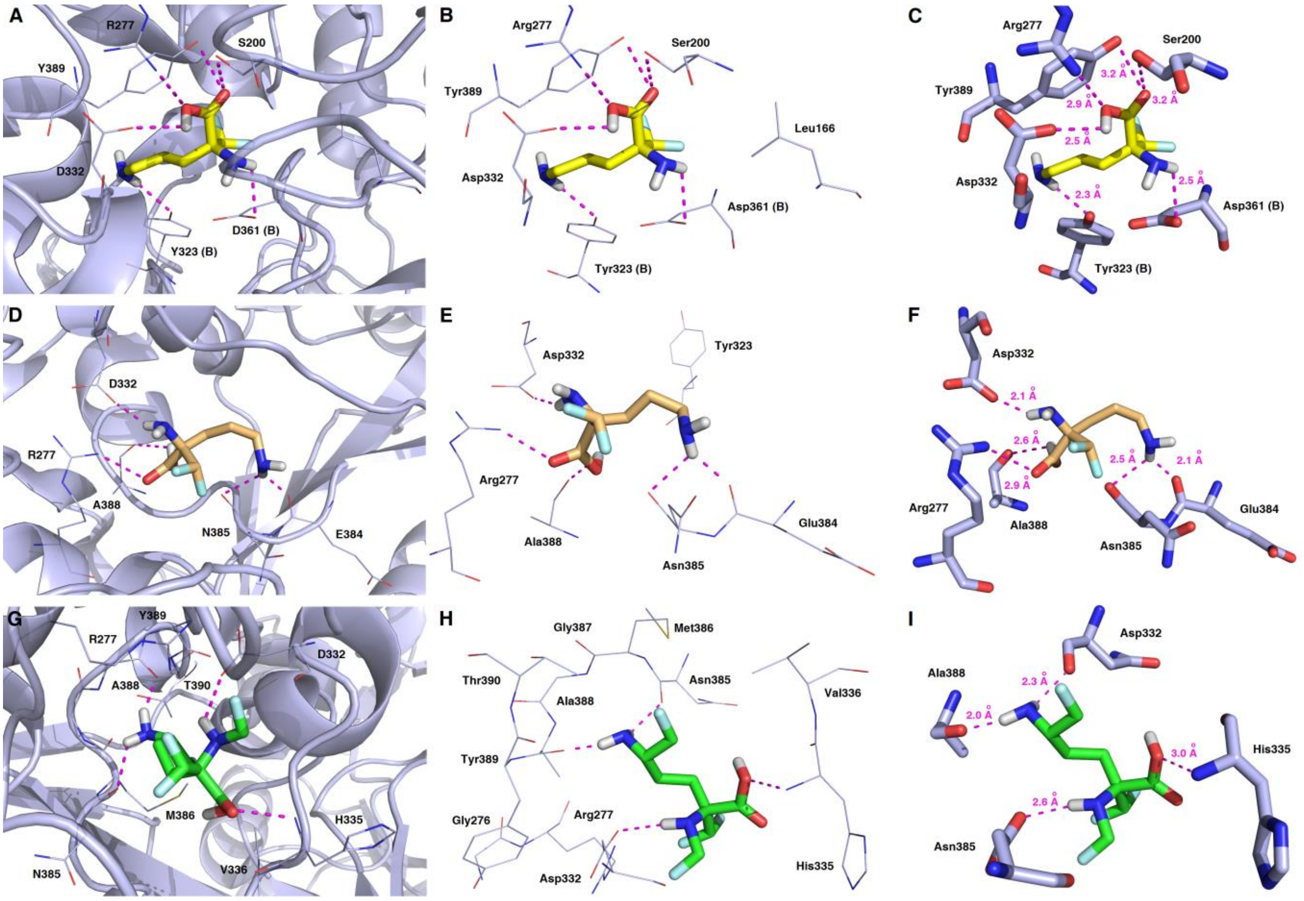

FIGURE S15 Binding modes of *L*-DFMO (**A**–**C**), *D*-DFMO (**D**–**F**), and the DFMO-analogue (**G**–**I**) to human ornithine decarboxylase without PLP-cofactor (h-ODC, PDB ID: 2OO0) (Dufe et al., 2007) with close contacts to residues in the active site. All nine *panels* (**A**–**I**) show inhibition of h-ODC by blocking substrate binding through direct interaction of the ligands with critical amino acids of the receptor. Ligands are shown as sticks coloured in yellow (*L*-DFMO), gold (*D*-DFMO) or green (DFMO-analogue), respectively. The overall enzyme structure (protein target) is shown as a light blue cartoon diagram (see **A**, **D**, **G**). The most significant amino acid residues contributing to the stabilization of the ligand molecules by polar interactions and by CH–CH van der Waals (vdW) interactions are shown in light blue lines (see **B**, **E**, **H**) or sticks (see **C**, **F**, **I**) representations. Nitrogen atoms are presented in blue, oxygen atoms in red, fluorine atoms in pale cyan, whereas hydrogen atoms in grey. The mutual distances between the amino acid residues, and the respective ligands' atoms are given in Ångström (see **C**, **F**, **I**). The formation of intermolecular hydrogen bonds is represented by magenta dashed lines.

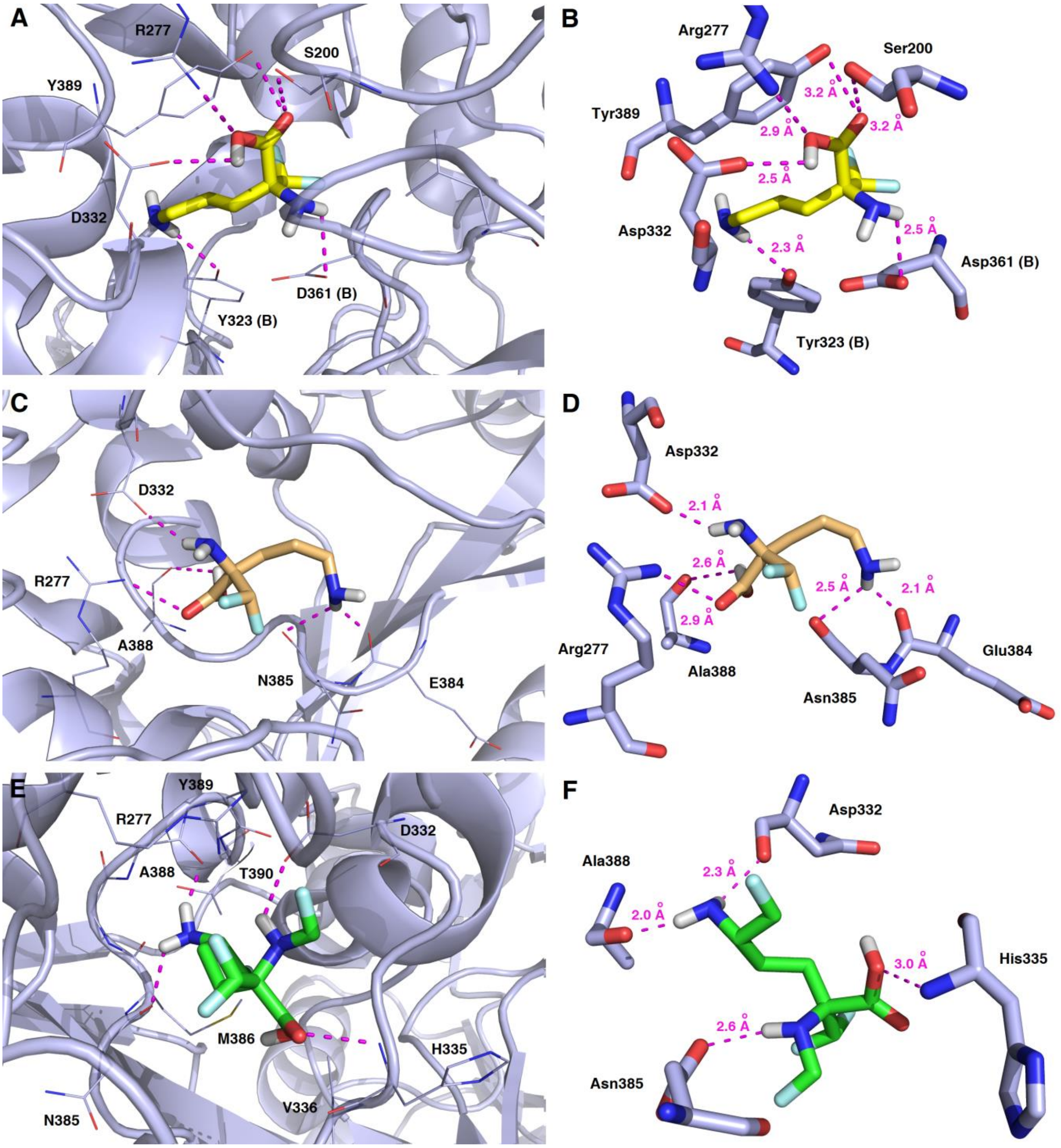

FIGURE S16 Representative binding modes of *L*-DFMO (**A**–**B**), *D*-DFMO (**C**–**D**), and the DFMO-analogue (**E**–**F**) to human ornithine decarboxylase without PLP-cofactor (h-ODC, PDB ID: 2OO0) (Dufe et al., 2007) with close contacts to residues in the active site. All six *panels* (**A**–**F**) show inhibition of h-ODC by blocking substrate-binding site through direct interaction of the ligands with critical amino acids of the receptor in catalytic cavity. Ligands are shown as sticks representation coloured in yellow (*L*-DFMO), gold (*D*-DFMO) or green (DFMO-analogue), respectively. The overall enzyme structure is shown as a light blue cartoon diagram (see **A**, **C**, **E**). The most significant amino acid residues contributing to the stabilization of the ligand molecules in the complex with h-ODC by polar interactions and by CH–CH van der Waals (vdW) interactions are shown in light blue sticks (see **B**, **D**, **F**). Nitrogen atoms are presented in blue, oxygen atoms in red, fluorine atoms in light blue, whereas hydrogen atoms (attached to nitrogen and/or oxygen atom of the carboxylic group) in grey. The mutual distances between the amino acid residues, and the respective ligands' atoms are given in Ångström (see **B**, **D**, **F**). The formation of intermolecular hydrogen bonds is represented by magenta dashed lines.

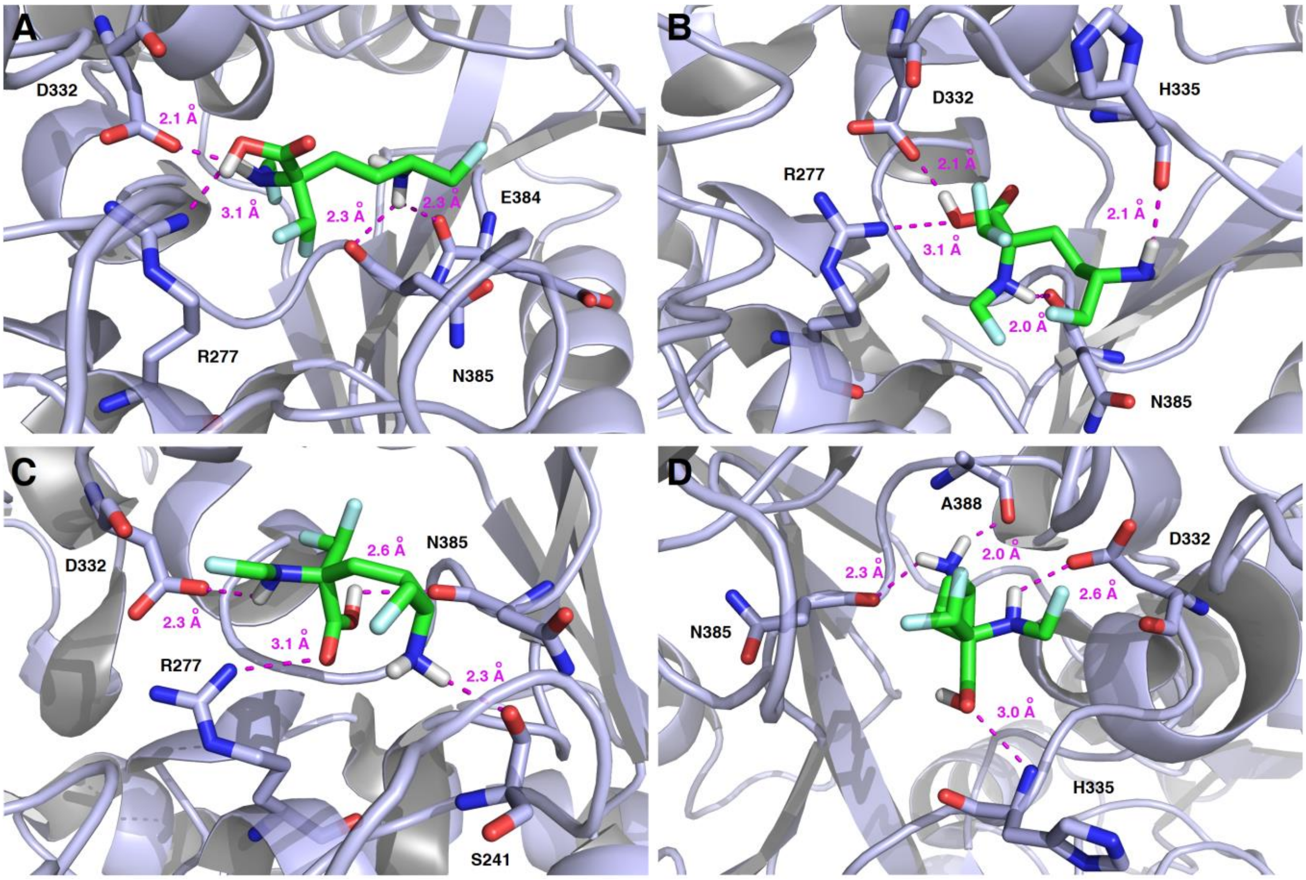

FIGURE S17 Predominant conformations of DFMO-analogue (green sticks) in the active site of h-ODC (PDB ID: 2OO0) (Dufe et al., 2007) (**A**–**D**) without PLP-cofactor. The overall target protein structure is shown as a light blue cartoon diagram with highlight color. The most significant amino acid residues contributing to the stabilization of the DFMO-analogue–h-ODC complex by polar interactions (H-bonding) and by CH–CH van der Waals (vdW) interactions are shown in light blue sticks. Nitrogen atoms are presented in blue, oxygen atoms in red, fluorine atoms in pale cyan, whereas hydrogen atoms (attached to nitrogen and/or oxygen atom of the carboxylic group) in grey. The formation of potential intermolecular hydrogen bonds is represented by magenta dashed lines. The mutual distances between the amino acid residues, and the respective ligands' atoms are given in Ångström.

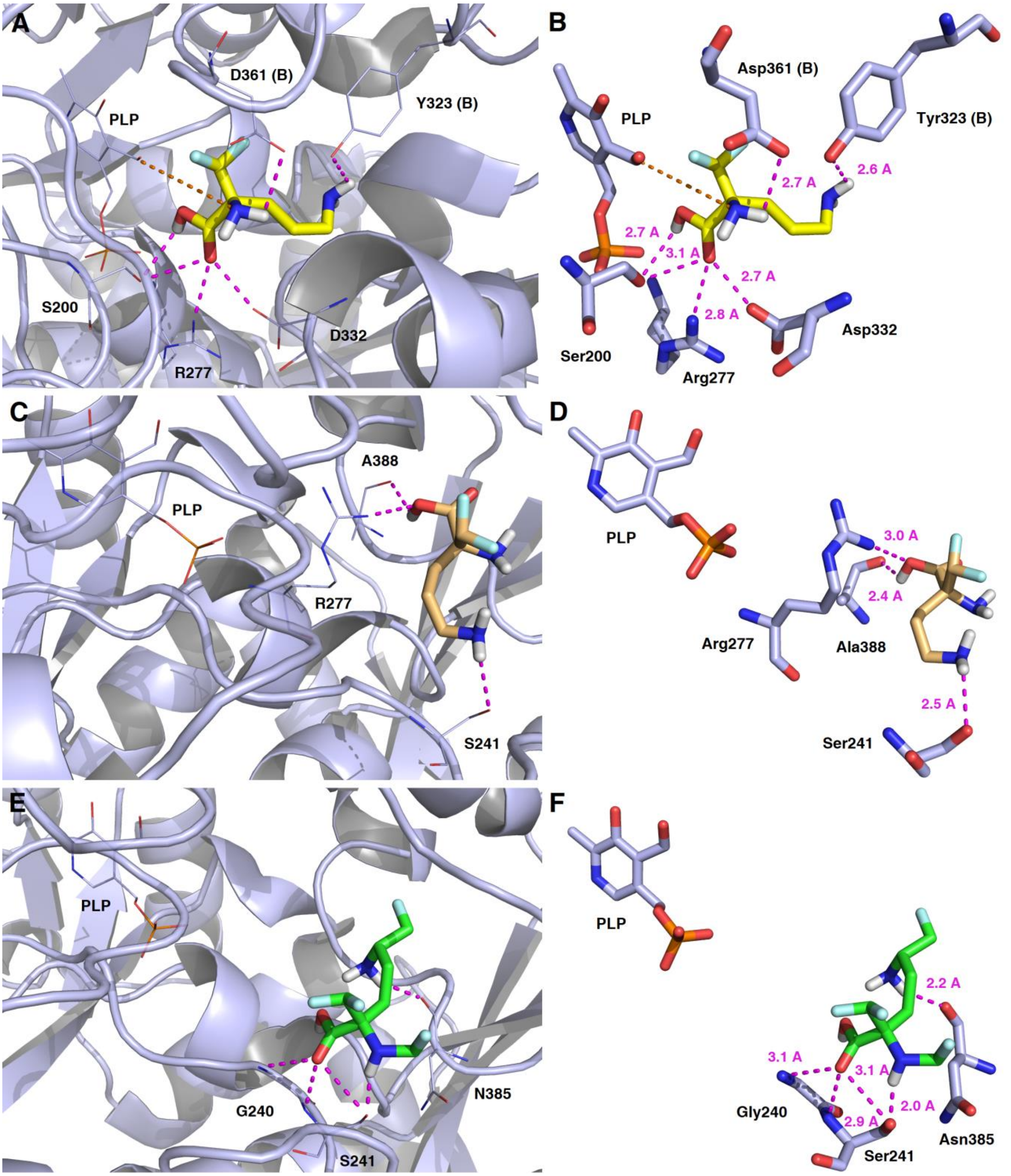

FIGURE S18 Representative binding modes of *L*-DFMO (**A**–**B**), *D*-DFMO (**C**–**D**), and the DFMO-analogue (**E**–**F**) to human ornithine decarboxylase complexed with PLP-cofactor (h-ODC, PDB ID: 2OO0) (Dufe et al., 2007) with close contacts to residues in the active site. All six *panels* (**A**–**F**) show inhibition of h-ODC by blocking substrate-binding site through direct interaction of the ligands with critical amino acids of the receptor in catalytic cavity. Ligands are shown as sticks representation coloured in yellow (*L*-DFMO), gold (*D*-DFMO) or green (DFMO-analogue), respectively. The overall enzyme structure is shown as a light blue cartoon diagram (see **A**, **C**, **E**). The most significant amino acid residues contributing to the stabilization of the ligand molecules in the complex with h-ODC by polar interactions and by CH–CH van der Waals (vdW) interactions are shown in light blue sticks (see **B**, **D**, **F**) representations. Nitrogen atoms are presented in blue, oxygen atoms in red, fluorine atoms in pale cyan, phosphorus atoms in orange, whereas hydrogen atoms (attached to

nitrogen and/or oxygen atom of the carboxylic group) in grey. The mutual distances between the amino acid residues (and PLP-cofactor) and the respective ligands' atoms are given in Ångström (see **B**, **D**, **F**). The formation of intermolecular hydrogen bonds is represented by magenta dashed lines. Orange dashed lines represents distance between α-amino group of *L*-DFMO and PLP-cofactor.

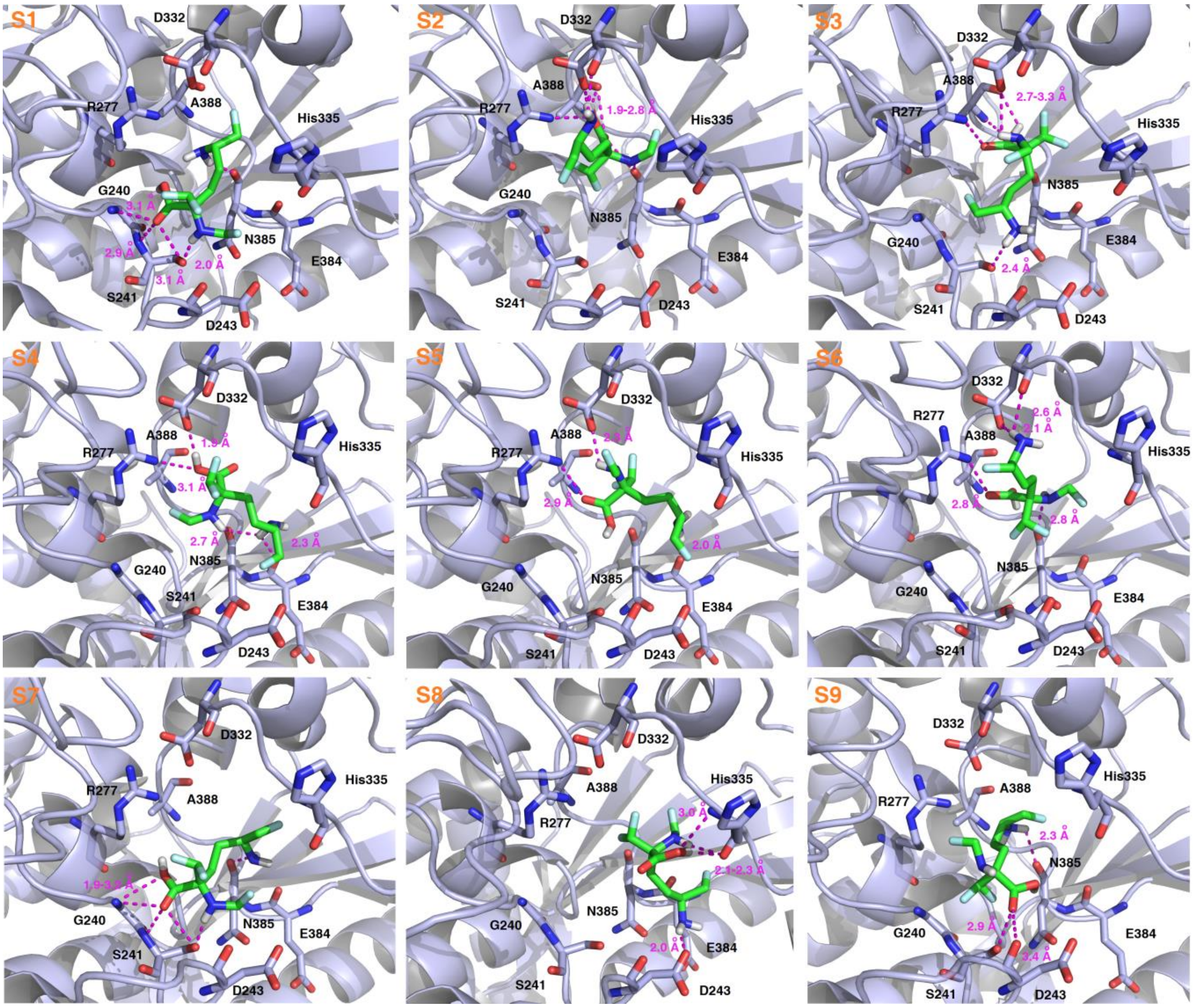

FIGURE S19 Binding mode proposals (**S1**–**S9**) for the complex of h-ODC (PDB ID: 2OO0, shown as light blue-colored cartoon diagram) (Dufe et al., 2007) with PLP-cofactor and DFMO-analogue (green sticks). The pose S1 represents the lowest value of ΔG_{calc} (kcal/mol), which means that its ligand-binding affinity to receptor (h-ODC active-site) is the highest, and in contrary, the S9 mode represent the lowest ligand-binding affinity. The formation of intermolecular hydrogen bonds is represented by magenta dashed lines. The mutual distances between the critical for catalytic active amino acid residues and the respective atoms of the DFMO-analogue are given in Ångström. Nitrogen atoms are presented in blue, oxygen atoms in red, fluorine atoms in pale cyan, phosphorus atoms in orange, whereas hydrogen atoms (attached to nitrogen and/or oxygen atom of the carboxylic group) in grey.

*
Dufe, V.T., Ingner, D., Khomutov, A.R., Heby, O., Persson, L., and Al-Karadaghi, S. (2007). A structural insight into the inhibition of human and Leishmania donovani ornithine decarboxylases by 3-aminooxy-1-aminopropane. https://doi.org/10.2210/pdb2OO0/pdb.

Dufe, V.T., Ingner, D., Heby, O., Khomutov, A.R., Persson, L., and Al-Karadaghi, S. (2007). A structural insight into the inhibition of human and Leishmania donovani ornithine decarboxylases by 1-amino-oxy-3-aminopropane. Biochem. J. 405, 261−268. https://doi.org/10.1042/BJ2007018

TABLES

TABLE S1 Results of X-ray study of **1**.

Formula	$C_6H_{15}ClF_2N_2O_3$
Molecular weight	236.65
Crystal system	orthorhombic
Space group	$Pca2_1$
a (Å)	10.41500(10)
b (Å)	8.96290(10)
c (Å)	10.70340(10)
V (Å^3)	999.147(17)
Z	4
ρ_{cal} (g/cm^3)	1.573
Temperature (K)	100
2θ range (^{o})	9.868 to 157.15
μ (mm^{-1})	3.620
Crystal dimensions (mm^3)	0.45 × 0.24 × 0.13
Collected reflections	28104
Data/restraints/parameters	2033/1/133
R_{int}	0.0436
F(000)	496
Index ranges	-12 ≤ h ≤ 13, -11 ≤ k ≤ 10, -13 ≤ l ≤ 12
GOF	1.067
R_1 for Fo^2 > 2o(Fo^2)	0.0204
wR_2 for Fo^2 > 2o(Fo2)	0.0542
Δφmax/Δφmin (θ/e Å-^{3})	0.25/-0.19

TABLE S2 Docking results of APA to h-ODC (PDB: 2OO0).

Mode[a]	*Affinity (kcal/mol)*[b]	*Dist. from best mode rmsd l.b.*	*Dist. from best mode rmsd u.b.*
**S1**	–3.8	0.000	0.000
**S2**	–3.7	1.478	2.732
**S3**	–3.7	2.041	2.652
**S4**	–3.7	2.323	2.965
**S5**	–3.6	2.383	2.999
**S6**	–3.6	1.926	2.016
**S7**	–3.4	1.550	1.820
**S8**	–3.4	1.775	2.078

**S9**	–3.3	2.494	2.663

[a] The pose S1 represents the lowest value of ΔG_{calc} (kcal/mol), which means that ligand-binding affinity to receptor (h-ODC site) is the highest, and in contrary, the S9 mode represent the lowest ligand-binding affinity.
[b] Average ΔG_{calc} = –3.57kcal/mol.

TABLE S3 Docking results of *L*-DFMO to h-ODC (PDB: 2OO0).

Mode[a]	*Affinity (kcal/mol)*[b]	*Dist. from best mode rmsd l.b.*	*Dist. from best mode rmsd u.b.*
S1	–4.7	0.000	0.000
S2	–4.6	27.697	28.323
S3	–4.6	17.755	19.190
S4	–4.5	27.751	28.955
S5	–4.5	26.074	27.726
S6	–4.4	38.143	38.910
S7	–4.4	17.632	18.411
S8	–4.4	28.543	29.603
S9	–4.4	27.986	28.427

[a] The pose S1 represents the lowest value of ΔG_{calc} (kcal/mol), which means that ligand-binding affinity to receptor (h-ODC site) is the highest, and in contrary, the S9 mode represent the lowest ligand-binding affinity.
[b] Average ΔG_{calc} = –4.50 kcal/mol.

TABLE S4 Docking results of *D*-DFMO to h-ODC (PDB: 2OO0).

Mode[a]	*Affinity (kcal/mol)*[b]	*Dist. from best mode rmsd l.b.*	*Dist. from best mode rmsd u.b.*
**S1**	–4.8	0.000	0.000
**S2**	–4.8	26.944	28.241
**S3**	–4.5	27.983	29.335
**S4**	–4.5	16.749	17.667
**S5**	–4.5	2.500	3.988
**S6**	–4.5	27.909	28.585
**S7**	–4.5	38.522	39.472
**S8**	–4.4	17.849	18.751
**S9**	–4.4	27.912	28.799

[a] see Table S1. [b] Average ΔG_{calc} = –4.54 kcal/mol.

TABLE S5 Docking results of DFMO-analogue to h-ODC (PDB: 2OO0).

Mode[a]	*Affinity (kcal/mol)*[b]	*Dist. from best mode rmsd l.b.*	*Dist. from best mode rmsd u.b.*
**S1**	–5.1	0.000	0.000
**S2**	–5.0	3.375	4.560
**S3**	–5.0	2.941	4.896
**S4**	–4.9	3.564	4.649

**S5**	–4.9	3.067	5.079
**S6**	–4.9	3.009	4.887
**S7**	–4.8	2.797	4.707
**S8**	–4.8	2.137	5.018
**S9**	–4.8	3.205	4.123

[a] see Table S1 and/or Table S2. [b] Average ΔG_{calc} = –4.91 kcal/mol.

TABLE S6 Docking results of *L*-DFMO to h-ODC with PLP (PDB: 2OO0).

Mode[a]	*Affinity (kcal/mol)*[b]	*Dist. from best mode rmsd l.b.*	*Dist. from best mode rmsd u.b.*
**S1**	–5.4	0.000	0.000
**S2**	–5.3	21.145	22.738
**S3**	–5.0	17.928	19.449
**S4**	–5.0	10.194	10.865
**S5**	–4.9	10.238	10.892
**S6**	–4.9	10.029	10.707
**S7**	–4.8	9.700	10.421
**S8**	–4.7	20.566	21.274
**S9**	–4.7	18.083	19.578

[a] The pose S1 represents the lowest value of ΔG_{calc} (kcal/mol), which means that ligand-binding affinity to receptor (h-ODC site) is the highest, and in contrary, the S9 mode represent the lowest ligand-binding affinity.
[b] Average ΔG_{calc} = –4.96 kcal/mol.

TABLE S7 Docking results of *D*-DFMO to h-ODC with PLP (PDB: 2OO0).

Mode[a]	*Affinity (kcal/mol)*[b]	*Dist. from best mode rmsd l.b.*	*Dist. from best mode rmsd u.b.*
**S1**	–5.0	0.000	0.000
**S2**	–5.0	26.974	28.226
**S3**	–4.9	2.598	3.708
**S4**	–4.8	2.593	3.712
**S5**	–4.8	12.305	13.523
**S6**	–4.7	2.664	3.717
**S7**	–4.7	27.534	28.753
**S8**	–4.7	19.942	21.118
**S9**	–4.6	12.792	14.008

[a] see Table S1. [b] Average ΔG_{calc} = –4.80 kcal/mol.

TABLE S8 Docking results of DFMO-analogue to h-ODC with PLP (PDB: 2OO0).

Mode[a]	Affinity (kcal/mol)[b]	Dist. from best mode rmsd l.b.	Dist. from best mode rmsd u.b.
**S1**	–5.1	0.000	0.000
**S2**	–5.1	3.184	4.796
**S3**	–5.1	2.418	4.971
**S4**	–5.0	3.064	4.748
**S5**	–4.9	3.133	4.991
**S6**	–4.9	2.987	4.473
**S7**	–4.9	1.529	1.639
**S8**	–4.9	3.500	5.104
**S9**	–4.8	2.260	3.071

[a] see Table S1 and/or Table S2. [b] Average ΔG_{calc} = –4.97 kcal/mol.

TABLE S9 The most important amino acid residues located among the h-ODC (PDB: 2OO0) substrate-binding site.

h-ODC-Chain A	*h-ODC-Chain B*
Lys(K)69, Asp(D)88, Arg(R)154, Leu(L)166, Lys(L)169, His(H)197, Ser(S)200, Cys(C)360, Cys(C)202, Gly(G)235, Gly(G)237, Pro(P)239, Ser(S)241, Leu(L)244, Glu(E)274, Gly(G)276, Arg(R)277, Tyr(Y)331, Asp(D)332, Tyr(Y)389.	Tyr(Y)323, Asp(D)361, Asn(N)385.

TABLE S10 The most important amino acid residues of h-ODC (PDB: 2OO0) involved in a hydrogen bond to the respective ligands.

L-DMFO	*L-DMFO*	*DMFO-analogue*
**Pose S1:** Asp(D)332, Ser(S)200, Tyr(Y)389, Arg(R)277, Tyr(Y)323[a], Asp(D)361.[a]	**Pose S1:** Asp(D)332, Arg(R)277, Ala(A)388, Asn(N)385, Glu(E)384.	**Pose S1:** Ser(S)241, Gly(G)240;<br>**Pose S2:** His(H)333, Arg(R)277;<br>**Pose S3:** Asp(D)332, Arg(R)277, Asn(N)385;<br>**Pose S4:** Arg(R)277, Ala(A)388, Glu(E)384;<br>**Pose S5:** Asp(D)332, Arg(R)277, Asn(N)385, Glu(E)384;<br>**Pose S6:** Asp(D)332, Arg(R)277;<br>**Pose S7:** Asp(D)332, His(H)335, Asn(N)385, Arg(R)277;<br>**Pose S8:** Asp(D)332, Arg(R)277, Ser(S)241, Asn(N)385;<br>**Pose S9:** Asp(D)332, His(H)335, Asn(N)385, Ala(A)388.

[a] Amino acids belonging to the subunit B of the h-ODC (h-ODC-Chain B).

TABLE S11 The most important amino acid residues of h-ODC (PDB: 2OO0) (containing PLP-cofactor) involved in a hydrogen bond to the respective ligands.

L-DMFO	*L-DMFO*	*DMFO-analogue*
**Pose S1:**	**Pose S6:**	**Pose S1:** Ser(S)241, Gly(G)240, Asn(N)385;

Asp(D)332, Ser(S)200, Arg(R)277, Tyr(Y)323[a], Asp(D)361.[a]	Arg(R)277, Ala(A)388, Ser(S)241.	**Pose S2:** Asp(D)322, Ala(A)388, Arg(R)277, Asn(N)385; **Pose S3:** Asp(D)332, Arg(R)277, Ser(S)241; **Pose S4:** Arg(R)277, Asp(D)332, Asn(N)385, Glu(E)384; **Pose S5:** Asp(D)332, Arg(R)277, Asn(N)385; **Pose S6:** Asp(D)332, Arg(R)277, Asn(N)385; **Pose S7:** Ser(S)241, Gly(G)240, Asn(N)385; **Pose S8:** Asp(D)243, His(H)335; **Pose S9:** Asp(D)332, His(H)335, Asn(N)385, Ala(A)388.

[a] Amino acids belonging to the subunit B of the h-ODC (h-ODC-Chain B).

TABLE S12 The results of the measurements of dihedral angels of *L*-DFMO docked to h-ODC (PDB: 2OO0).

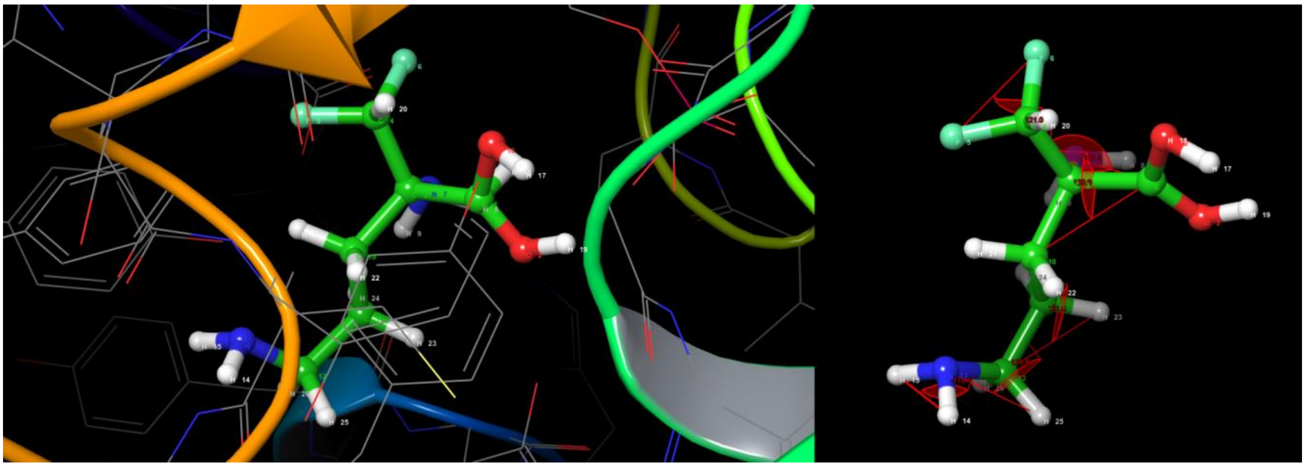

Atom 1	*Atom 2*	*Atom 3*	*Atom 4*	*Dihedral*	*Name*
C:4	C:3	C:1	C:10	120.0	C-C-C-C
C:4	C:3	N:7	C:1	116.3	C-C-N-C
F:5	F:6	C:4	C:3	121.0	F-F-C-C
C:10	C:11	H:23	C:12	121.8	C-C-1HXT-C
C:11	C:12	H:25	N:13	120.8	C-C-1HXT-N
H:15	H:14	N:13	C:12	–118.1	H-H-N-C

TABLE S13 The results of the measurements of dihedral angels of DFMO-analogue docked to h-ODC (PDB: 2OO0).

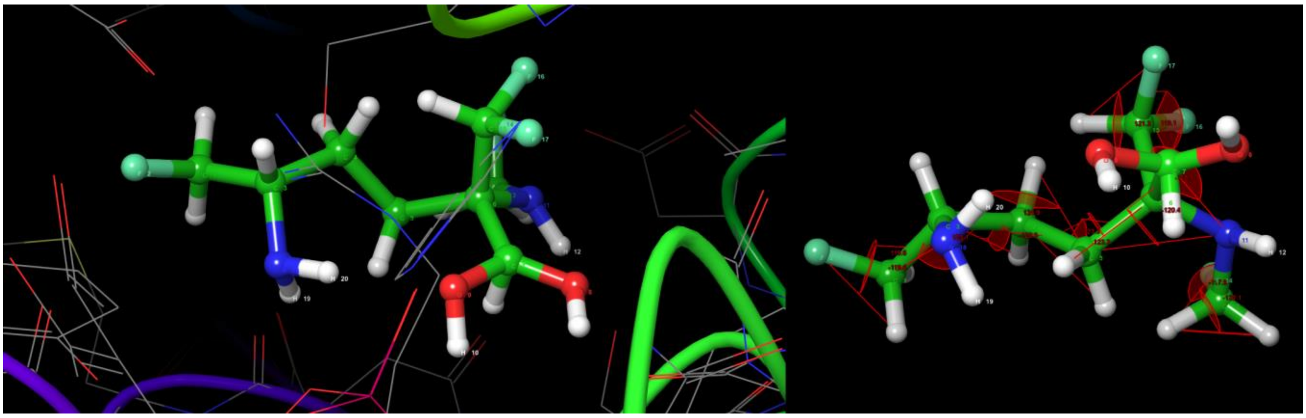

Atom 1	Atom 2	Atom 3	Atom 4	Dihedral	Name
C:3	C:1	F:2	H:21	119.6	C2-C1-F1-H1_1
C:3	C:1	F:2	H:22	–119.6	C2-C1-F1-H1_2
C:3	C:4	H:24	C:5	124.9	C2-C3-H3_1-C4
C:4	C:3	C:1	N:18	123.1	C3-C2-C1-N1
C:4	C:5	C:6	H:27	123.3	C3-C4-C5-H4_2
C:5	C:4	C:3	H:25	–122.5	C4-C3-C2-H3_2
C:6	C:5	C:4	H:26	123.3	C5-C4-C3-H4_1
C:6	C:15	H:32	F:17	121.3	C5-C7-HC7-F3
C:7	C:6	C:5	N:11	–120.4	C6-C5-C4-N2
N:11	C:13	F:14	H:30	–117.9	N2-C8-F4-H8_1
N:11	C:13	H:30	H:31	–122.1	N2-C8-H8_1-H8_2
C:15	C:6	C:7	N:11	–118.9	C7-C5-C6-N2
F:17	C:15	F:16	C:6	119.1	F3-C7-F2-C5

TABLE S14 Selected bond distances and angles of **1**.

Bond distance (Å)		*Bond angle (°)*		*Torsion angles (°)*	
F1-C3	1.367(2)	O1-C2-O2	127.12(17)	F1-C3-C1-N1	-49.90(18)
F2-C3	1.375(2)	O1-C2-C1	116.92(17)	F1-C3-C1-C2	-170.50(14)
O1-C2	1.242(2)	O2-C2-C1	115.85(15)	F1-C3-C1-C4	70.01(18)
O2-C2	1.260(2)	F1-C3-F2	105.55(14)	F2-C3-C1-N1	65.36(18)
N1-C1	1.495(2)	F1-C3-C1	109.16(14)	F2-C3-C1-C2	-55.2(2)
N2-C6	1.491(2)	F2-C3-C1	109.79(14)	F2-C3-C1-C4	-174.73(15)
C2-C1	1.557(3)	N1-C1-C2	109.68(14)	O1-C2-C1-N1	20.6(2)
C3-C1	1.522(2)	N1-C1-C3	108.02(15)	O1-C2-C1-C3	140.27(16)
C1-C4	1.540(2)	N1-C1-C4	110.63(14)	O1-C2-C1-C4	-100.36(18)
C5-C4	1.530(2)	C3-C1-C2	111.53(14)	O2-C2-C1-N1	-162.95(14)
C5-C6	1.519(3)	C3-C1-C4	108.39(14)	O2-C2-C1-C3	-43.3(2)
		C4-C1-C2	108.59(15)	O2-C2-C1-C4	76.05(19)
		C6-C5-C4	108.73(14)	N1-C1-C4-C5	-56.0(2)
		C5-C4-C1	114.48(14)	C2-C1-C4-C5	64.44(19)
		N2-C6-C5	112.02(15)	C3-C1-C4-C5	-174.24(15)
				C4-C5-C6-N2	-165.84(15)
				C6-C5-C4-C1	-171.02(16)

TABLE S15 Geometrical parameters of H-bonds in the crystal structure o*f 1.*

H-bond	*Symmetry code*	*D-H (Å)*	*H···A (Å)*	*D···A (Å)*	*H···A (°)*
N1-H1a···Cl1		0.91	2.40	3.2324	151
N1-H1a···O1*		0.91	2.24	2.6775	109
N1-H1b···O3		0.91	2.01	2.9192	174
N1-H1c···F2*		0.91	2.43	2.8571	109
N1-H1c···O2	*3/2-x, y, ½+z*	0.91	1.93	2.7991	159
N2-H2a···Cl1	*1-x, -y, -1/2+z*	0.91	2.30	3.1511	156
N2-H2b···Cl1	*-1/2+x, -y, z*	0.91	2.29	3.1531	158
N2-H2c···O3	*1-x, -y, -1/2+z*	0.91	2.08	2.9043	150
O3-H3a···O2	*1-x, 1-y, ½+z*	0.87	1.85	2.7154	172
O3-H3b···Cl1	*-1/2+x, -y, z*	0.87	2.30	3.1067	154
C3-H3···O3	*1-x, -y, -1/2+z*	1.00	2.54	3.4213	146
C3-H3···O1	*-1/2+x, 1-y, z*	1.00	2.45	3.1717	129

*Intramolecular H-bonds

TABLE S16 Optimized dihedral angles (degrees) of the DFMO species studied in the gas phase (M06) and in aqueous solution (M06/CPCM) and their comparison with experimental data.

Dihedral angle	*M06*	*M06/CPCM*	*X-ray from PDB*	*X-ray Solid State of DFMO*
**2-(Difluoromethyl)ornithine**				
α[C(3)-C(1)-C(4)-C(5)]	175.29	176.51	-179.94[a]	
β[C(1)-C(4)-C(5)-C(6)]	176.08	174.89	146.01	
γ[C(4)-C(5)-C(6)-N(2)]	178.24	179.06	-179.74	
δ[C(5)-C(4)-C(1)-C(2)]	-67.29	-66.09	-61.65	
ε[C(5)-C(4)-C(1)-N(1)]	54.14	56.26	59.70	
**2-(Difluoromethyl)ornithine protonated cation at the terminus basic nitrogen**				
α[C(3)-C(1)-C(4)-C(5)]	-175.88	-178.59		
β[C(1)-C(4)-C(5)-C(6)]	130.82	132.00		
γ[C(4)-C(5)-C(6)-N(2)]	-73.44	-75.17		
δ[C(5)-C(4)-C(1)-C(2)]	-58.45	-60.98		
ε[C(5)-C(4)-C(1)-N(1)]	64.09	61.37		
**2-(Difluoromethyl)ornithine hydrochloride hydrate (A structure)**				
α[C(3)-C(1)-C(4)-C(5)]	175.21	174.60		
β[C(1)-C(4)-C(5)-C(6)]	59.53	71.61		
γ[C(4)-C(5)-C(6)-N(2)]	-87.17	-95.10		
δ[C(5)-C(4)-C(1)-C(2)]	-66.36	-65.91		
ε[C(5)-C(4)-C(1)-N(1)]	56.09	56.83		
**2-(Difluoromethyl)ornithine hydrochloride hydrate (B structure)**				
α[C(3)-C(1)-C(4)-C(5)]	-175.16	-170.53		174.26
β[C(1)-C(4)-C(5)-C(6)]	175.33	-179.97		171.09
γ[C(4)-C(5)-C(6)-N(2)]	-175.18	-172.24		165.81

δ[C(5)-C(4)-C(1)-C(2)]	-59.20	-54.62		-64.41
ε[C(5)-C(4)-C(1)-N(1)]	61.07	64.75		55.95
**2-(difluoromethyl)ornithine hydrochloride hydrate (C structure)**				
α[C(3)-C(1)-C(4)-C(5)]	-164.14	-176.78		
β[C(1)-C(4)-C(5)-C(6)]	130.76	161.82		
γ[C(4)-C(5)-C(6)-N(2)]	-66.46	-77.78		
δ[C(5)-C(4)-C(1)-C(2)]	-48.41	-61.54		
ε[C(5)-C(4)-C(1)-N(1)]	71.88	58.29		

[a]3GN0.pdb [Ilies, 2011]

TABLE S17 Relative enthalpy, ΔH**,** entropy, ΔS, and Gibbs energy, ΔG, of the three structures of 2-(difluoromethyl)ornithine hydrochloride hydrate computed at the M06/6-311++G(d,p) level of theory (T=298.15 K).

Complex	*Gas-phase structures*			*Solvated systems*		
	ΔH, kJ/mol	*ΔS, J/K.mol*	*ΔG, kJ/mol*	*$ΔH^C_{PCM}$, kJ/mol*	*$ΔS^C_{PCM}$, J/K.mol*	*$ΔG^C_{PCM}$, kJ/mol*
A	0	-5.19	1.55	7.90	-5.71	9.60
B	5.59	18.17	0	0	0	0
C	1.52	2.88	0.66	8.36	8.75	5.75

TABLE S18 Hirshfeld interaction surfaces and ERs in **1** and **2**.

	H		*O*		*C*		*Cl*		*F*
compound	**1**	**2**	**1**	**2**	**1**	**2**	**1**	**2**	**1**
Surface (%)	67.7	70.6	12.8	15.3	0.6	0.85	5.3	13.05	12
ER									
H	**0.83**	**0.85**							
O	**1.48**	**1.42**							
C	**1.36**	**1.42**							
Cl	**1.49**	**1.42**							
F	**1.35**		**0.65**						

TABLE S19 Inter-contact energy values (kJ/mol) for **1** and **2**. *R* is the distance (Å) between molecular centroids (mean atomic position).

Symmetry code	*R*	*E_{ele}*	*E_{pol}*	*E_{dis}*	*E_{rep}*	*E_{tot}*
**1**						
x+1/2, -y, z	8.70	-10.3	-6.0	-10.9	31.4	-5.4
	4.69	1.0	-0.1	-0.5	0.0	0.6
	6.04	2.0	-0.1	-0.1	0.0	2.0
	4.26	4.1	-0.5	-4.0	2.0	1.7

-x, -y, z+1/2	6.01	-5.5	-5.1	-16.0	9.1	-17.9
-x+1/2, y, z+1/2	6.31	-85.0	-25.3	-14.5	51.4	-89.4
x+1/2, -y, z	5.57	-21.3	-12.2	-26.6	25.6	-39.0
x, y, z	8.96	4.1	-0.5	-4.0	2.0	1.7
-x, -y, z+1/2	8.99	3.7	-2.4	-5.5	5.7	0.8
	5.23	-21.3	-12.2	-26.6	25.6	-39.0
	6.76	-5.5	-5.1	-16.0	9.1	-17.9
		-134	-69.5	-124.7	161.9	-201.8
Total						
**2**						
	4.91	-0.2	-1.6	-0.9	0.0	-2.2
-x, y+1/2, -z	8.10	2.4	-0.8	-6.3	4.2	-1.0
	5.33	-144.0	-43.3	-26.9	139.8	-121.3
-x, y+1/2, -z	6.04	-49.6	-14.6	-15.5	17.7	-65.7
x, y, z	7.99	10.7	-2.3	-5.8	5.2	7.7
	5.14	-2.3	-0.1	-0.1	0.0	-2.5
x, y, z	5.00	-22.1	-26.2	-21.8	62.7	-23.1
-x, y+1/2, -z	5.15	-144.0	-43.3	-26.9	139.8	-121.3
	4.38	-3.0	-0.0	-0.0	0.0	-3.2
x, y, z	9.43	5.1	-1.1	-1.8	1.1	3.6
	6.58	-6.2	-0.7	-0.4	0.0	-7.3
	6.00	-5.9	-0.2	-0.1	0.0	-6.5
Total		-359.1	-134.2	-106.5	370.5	-342.8

Scale factors for E_{tot}: k_{ele} = 1.057, k_{pol} = 0.740, k_{disp} = 0.871, k_{rep} = 0.618 (MacKenzie et al., 2017).

TABLE S20 Molecular interaction energies (kJ/mol).

Interaction Energies (kJ/mol)
R is the distance between molecular centroids (mean atomic position) in Å.
Total energies, only reported for two benchmarked energy models, are the sum of the four energy components, scaled appropriately (see the scale factor table below)

	N	Symop	R	Electron Density	E_ele	E_pol	E_dis	E_rep	E_tot
	2	x+1/2, -y, z	8.70	B3LYP/6-31G(d,p)	-10.3	-6.0	-10.9	31.4	-5.4
	1	-	6.69	B3LYP/6-31G(d,p)	0.0	nan	0.0	0.0	nan
	1	-	4.69	B3LYP/6-31G(d,p)	1.0	-0.1	-0.5	0.0	0.6
	1	-	6.04	B3LYP/6-31G(d,p)	2.0	-0.1	-0.1	0.0	2.0
	1	-	4.26	B3LYP/6-31G(d,p)	4.1	-0.5	-4.0	2.0	1.7
	2	-x, -y, z+1/2	6.01	B3LYP/6-31G(d,p)	-5.5	-5.1	-16.0	9.1	-17.9
	2	-x+1/2, y, z+1/2	6.31	B3LYP/6-31G(d,p)	-85.0	-25.3	-14.5	51.4	-89.4
	2	x+1/2, -y, z	5.57	B3LYP/6-31G(d,p)	-21.3	-12.2	-26.6	25.6	-39.0
	2	x, y, z	8.96	B3LYP/6-31G(d,p)	4.1	-0.5	-4.0	2.0	1.7
	2	-x, -y, z+1/2	8.99	B3LYP/6-31G(d,p)	3.7	-2.4	-5.5	5.7	0.8
	1	-	5.23	B3LYP/6-31G(d,p)	-21.3	-12.2	-26.6	25.6	-39.0
	1	-	6.76	B3LYP/6-31G(d,p)	-5.5	-5.1	-16.0	9.1	-17.9

Energy Model	k_ele	k_pol	k_disp	k_rep
CE-HF ... HF/3-21G electron densities	1.019	0.651	0.901	0.811
CE-B3LYP ... B3LYP/6-31G(d,p) electron densities	1.057	0.740	0.871	0.618

1

	N	Symop	R	Electron Density	E_ele	E_pol	E_dis	E_rep	E_tot
	0	-	9.86	B3LYP/6-31G(d,p)	0.0	nan	0.0	0.0	nan
	1	-	4.91	B3LYP/6-31G(d,p)	-0.2	-1.6	-0.9	0.0	-2.2
	2	-x, y+1/2, -z	8.10	B3LYP/6-31G(d,p)	2.4	-0.8	-6.3	4.2	-1.0
	1	-	5.33	B3LYP/6-31G(d,p)	-144.0	-43.3	-26.9	139.8	-121.3
	2	-x, y+1/2, -z	6.04	B3LYP/6-31G(d,p)	-49.6	-14.6	-15.5	17.7	-65.7
	2	x, y, z	7.99	B3LYP/6-31G(d,p)	10.7	-2.3	-5.8	5.2	7.7
	1	-	5.14	B3LYP/6-31G(d,p)	-2.3	-0.1	-0.1	0.0	-2.5
	2	x, y, z	5.00	B3LYP/6-31G(d,p)	-22.1	-26.2	-21.8	62.7	-23.1
	2	-x, y+1/2, -z	5.15	B3LYP/6-31G(d,p)	-144.0	-43.3	-26.9	139.8	-121.3
	1	-	4.38	B3LYP/6-31G(d,p)	-3.0	-0.0	-0.0	0.0	-3.2
	2	x, y, z	9.43	B3LYP/6-31G(d,p)	5.1	-1.1	-1.8	1.1	3.6
	1	-	6.58	B3LYP/6-31G(d,p)	-6.2	-0.7	-0.4	0.0	-7.3
	1	-	6.00	B3LYP/6-31G(d,p)	-5.9	-0.2	-0.1	0.0	-6.5

Energy Model	k_ele	k_pol	k_disp	k_rep
CE-HF ... HF/3-21G electron densities	1.019	0.651	0.901	0.811
CE-B3LYP ... B3LYP/6-31G(d,p) electron densities	1.057	0.740	0.871	0.618

2

TABLE S21 Gallery of H-bonding patterns in analysed crystals (up to 20-membered motifs are considered).

compound	*synthon*	*interaction*
**1**	D(2)	*(H2O)Ow-Hw1···**O2(COO-)**
		*(H2O)Ow-Hw2···Cl1(Cl-)
		***(NH3+)N1**-Hn3···Ow(H2O)
		***(NH3+)N2-Hn5···Ow(H2O)**
		***(NH3+)N1**-Hn1···Cl1(Cl-)
		***(NH3+)N2**-Hn4···Cl1(Cl-)
		*(CH)C3-H3···Ow(H2O)
		*(CH2)C5-H5b···Cl1(Cl-)
	S(6)	(CH2)C5-H5b···**O1(COO-)**
	C(5)	***(NH3+)N1**-Hn1···**O2(COO-)**
		*(CH)C3-H3···**O1(COO-)**
		*(CH2)C4-H4a···F1
		*(CH2)C4-H4b···F2
	C(7)	(CH2)C6-H6a···F1
Level 2		
	$C^1_2(6)$	***(NH3+)**N1-Hn3···Ow(H2O) & (CH)C3-H3···Ow(H2O)
		***(NH3+)N2**-Hn4···Cl1(Cl-) & (CH2)C5-H5b···Cl1(Cl-)
		***(NH3+)N2**-Hn6···Cl1(Cl-) & (CH2)C5-H5b···Cl1(Cl-)
	$C^1_2(7)$	**(NH3+)N1**-H1n···Cl1(Cl-) & (CH2)C5-H5b···Cl1(Cl-)
	$C^1_2(9)$	***(NH3+)N1**-H1n···Cl1(Cl-) & **(NH3+)N2**-Hn4···Cl1(Cl-)
		***(NH3+)N1**-H1n···Cl1(Cl-) & **(NH3+)N2**-Hn6···Cl1(Cl-)
		***(NH3+)N1**-Hn3···Ow(H2O) & **(NH3+)N2**-Hn5···Ow(H2O)
		***(NH3+)N2**-Hn5···Ow(H2O) & (CH)C3-H3···Ow(H2O)
	$C^2_2(6)$	(CH2)C4-H4a···F1 & (CH)C4-H4b···F2
	$C^2_2(7)$	**(NH3+)N1**-Hn3···Ow(H2O) & (H2O)Ow-Hw1···**O2(COO-)**
	$C^2_2(8)$	***(NH3+)N1**-Hn2···O2(COO-) & (CH)C3-H3···**O1(COO-)**
		*(CH)C3-H3···**O1(COO-)** & (CH2)C4-H4a···F1
		*(CH)C3-H3···**O1(COO-)** & (CH)C4-H4b···F2
		*(CH)C4-H4b···F2 & (CH2)C6-H6a···F1
	$C^2_2(10)$	***(NH3+)N1**-Hn2···**O2(COO-)** & (CH)C3-H3···**O1(COO-)**
		*(NH3+)N1-Hn2···O2(COO-) & (CH2)C4-H4a···F1
		***(NH3+)N1**-Hn2···**O2(COO-)** & (CH2)C4-H4b···F2
		***(NH3+)N2**-Hn5···Ow(H2O) & (H2O)Ow-Hw1···**O2(COO-)**
		*(CH)C3-H3···**O1(COO-)** & (CH)C4-H4b···F2
		*(CH)C3-H3···**O1(COO-)** & (CH2)C6-H6a···F1
		*(CH2)C4-H4a···F1 & (CH)C4-H4b···F2
	$C^2_2(12)$	***(NH3+)N1**-Hn2···**O2(COO-)** & (CH2)C6-H6a···F1
		*(CH2)C4-H4a···F1 & (CH2)C6-H6a···F1
		*(CH2)C4-H4b···F2 & (CH2)C6-H6a···F1
	$C^4_4(16)$	[(CH2)C4-H4a···F1]2 & [(CH)C4-H4b···F2]2
	$C^4_4(18)$	*[**(NH3+)N1**-Hn2···**O2(COO-)**]2 & [(CH)C3-H3···**O1(COO-)**]2
		*[(CH)C3-H3···**O1(COO-)**]2 & [(CH)C4-H4b···F2]2
	$C^4_4(20)$	*[**(NH3+)N1**-Hn2···**O2(COO-)**]2 & [(CH2)C4-H4a···F1]2
		*[**(NH3+)N1**-Hn2···**O2(COO-)**]2 & [(CH2)C4-H4b···F2]2
		*[(CH2)C4-H4a···F1]2 & [(CH2)C4-H4b···F2]2
	$R^1_2(6)$	(CH2)C4-H4a···F1 & (CH2)C6-H6a···F1
	$R^2_2(7)$	(CH)C3-H3···Ow(H2O) & (H2O)Ow-Hw1···**O2(COO-)**
	$R^2_2(10)$	(CH)C3-H3···**O1(COO-)** & (CH2)C4-H4a···F1
	$R^2_2(12)$	(CH)C3-H3···**O1(COO-)** & (CH2)C6-H6a···F1
	$R^4_4(16)$	[(CH2)C4-H4a···F1]2 & [(CH)C4-H4b···F2]2
	$R^4_4(18)$	*[**(NH3+)N1**-Hn2···**O2(COO-)**]2 & [(CH)C3-H3···**O1(COO-)**]2
		*[(CH)C3-H3···O1(COO-)]2 & [(CH)C4-H4b···F2]2
	$R^4_4(20)$	*[**(NH3+)N1**-Hn2···**O2(COO-)**]2 & [(CH2)C4-H4a···F1]2
		*[**(NH3+)N1**-Hn2···**O2(COO-)**]2 & [(CH)C4-H4b···F2]2
		*[(CH)C4-H4b···F2]2 & (CH2)C6-H6a···F1
	$D^1_2(3)$	***(NH3+)N1**-H1n···Cl1(Cl-) & (H2O)Ow-Hw2···Cl1(Cl-)
		* **(NH3+)N2**-Hn6···Cl1(Cl-) & (H2O)Ow-Hw2···Cl1(Cl-)
		*(CH2)C5-H5b···Cl1(Cl-) & (H2O)Ow-Hw2···Cl1(Cl-)
	$D^2_2(4)$	***(NH3+)N1**-Hn3···Ow(H2O) & (H2O)Ow-Hw2···Cl1(Cl-)
		***(NH3+)N2**-Hn5···Ow(H2O) & (H2O)Ow-Hw2···Cl1(Cl-)
		*(CH)C3-H3···Ow(H2O) & (H2O)Ow-Hw2···Cl1(Cl-)
	$D^2_2(5)$	*(H2O)Ow-Hw1···**O2(COO-)** & (H2O)Ow-Hw2···Cl1(Cl-)
		***(NH3+)N1**-Hn3···Ow(H2O) & **(NH3+)N1**-H1n···Cl1(Cl-)

		*(NH3+)**N2**-Hn5···Ow(H2O) & (NH3+)**N2**-Hn6···Cl1(Cl-)
		* (NH3+)**N2**-Hn4···Cl1(Cl-) & (NH3+)**N2**-Hn6···Cl1(Cl-)
	$D^2_2(7)$	*(H2O)Ow-Hw1···**O2(COO-)** & (NH3+)**N1**-H1n···Cl1(Cl-)
		*(NH3+)**N1**-H1n···Cl1(Cl-) & (CH)C3-H3···Ow(H2O)
		*(NH3+)**N2**-Hn5···Ow(H2O) & (CH2)C5-H5b···Cl1(Cl-)
	$D^2_2(8)$	*(H2O)Ow-Hw1···**O2(COO-)** & (CH2)C5-H5b···Cl1(Cl-)
		*(NH3+)**N1**-Hn3···Ow(H2O) & (CH2)C5-H5b···Cl1(Cl-)
		*(CH)C3-H3···Ow(H2O) & (CH2)C5-H5b···Cl1(Cl-)
	$D^2_2(10)$	*(NH3+)**N1**-H1n···Cl1(Cl-) & (NH3+)**N2**-Hn5···Ow(H2O)
		*(NH3+)**N1**-Hn3···Ow(H2O) & (NH3+)**N2**-Hn4···Cl1(Cl-)
		*(NH3+)**N1**-Hn3···Ow(H2O) & (NH3+)**N2**-Hn6···Cl1(Cl-)
		*(NH3+)**N2**-Hn4···Cl1(Cl-) & (H2O)Ow-Hw1···**O2(COO-)**
		*(NH3+)**N2**-Hn4···Cl1(Cl-) & (CH)C3-H3···Ow(H2O)
		*(NH3+)**N2**-Hn6···Cl1(Cl-) & (CH)C3-H3···Ow(H2O)
		*[(NH3+)**N2**-Hn6···Cl1(Cl-)]$_2$ & (H2O)Ow-Hw1···**O2(COO-)**
	$D^2_3(8)$	*[(H2O)Ow-Hw1···**O2(COO-)**]$_2$ & (NH3+)**N1**-Hn2···**O2(COO-)**
		*[(CH)C3-H3···Ow(H2O)]$_2$ & (CH)C3-H3···**O1(COO-)**
	$D^3_3(10)$	*[(NH3+)**N1**-H1n···Cl1(Cl-)]$_2$ & (NH3+)**N1**-Hn2···**O2(COO-)**
		*(NH3+)**N1**-Hn2···**O2(COO-)** & [(NH3+)**N1**-Hn3···Ow(H2O)]$_2$
		*[(H2O)Ow-Hw1···**O2(COO-)**]$_2$ & (CH)C3-H3···**O1(COO-)**
		*(CH)C3-H3···Ow(H2O) & [(CH2)C4-H4a···F1]$_2$
		*[(CH)C3-H3···Ow(H2O)]$_2$ & (CH2)C4-H4b···F2
	$D^3_3(12)$	*[(H2O)Ow-Hw1···**O2(COO-)**]$_2$ & (CH)C4-H4a···F1
		*[(H2O)Ow-Hw1···**O2(COO-)**]$_2$ & (CH)C4-H4b···F2
		*[(NH3+)**N1**-H1n···Cl1(Cl-)]$_2$ & (CH)C3-H3···**O1(COO-)**
		*[(NH3+)**N1**-H1n···Cl1(Cl-)]$_2$ & (CH2)C4-H4a···F1
		*[(NH3+)**N1**-H1n···Cl1(Cl-)]$_2$ & (CH2)C4-H4b···F2
		*(NH3+)**N1**-Hn2···O2(COO-) & [(CH)C3-H3···Ow(H2O)]$_2$
		*[(NH3+)**N1**-Hn3···Ow(H2O)]$_2$ & (CH)C3-H3···**O1(COO-)**
		*[(NH3+)**N1**-Hn3···Ow(H2O)] & (CH2)C4-H4a···F1
		*[(NH3+)**N1**-Hn3···Ow(H2O)] & (CH)C4-H4b···F2
		* (CH)C3-H3···Ow(H2O) & (CH2)C6-H6a···F1
		*(CH)C4-H4a···F1 & [(CH2)C5-H5b···Cl1(Cl-)]$_2$
		*(CH)C4-H4b···F2 & [(CH2)C5-H5b···Cl1(Cl-)]$_2$
		*[(CH2)C5-H5b···Cl1(Cl-)]$_2$ & (CH2)C6-H6a···F1
	$D^3_3(14)$	*(NH3+)**N1**-Hn2···**O2(COO-)** & [(CH2)C5-H5b···Cl1(Cl-)]$_2$
		*[(NH3+)**N1**-Hn3···Ow(H2O)]$_2$ & (CH2)C6-H6a···F1
		*[(NH3+)**N1**-H1n···Cl1(Cl-)]$_2$ & (CH2)C6-H6a···F1
		*[(NH3+)**N2**-Hn5···Ow(H2O)]$_2$ & (CH2)C6-H6a···F1
		*[(NH3+)**N2**-Hn4···Cl1(Cl-)]$_2$ & (CH2)C6-H6a···F1
		*[(NH3+)**N2**-Hn6···Cl1(Cl-)]$_2$ & (CH2)C6-H6a···F1
		*[(H2O)Ow-Hw1···**O2(COO-)**]$_2$ & (CH2)C6-H6a···F1
		*(CH)C3-H3···**O1(COO-)** & [(CH2)C5-H5b···Cl1(Cl-)]$_2$
	$D^3_3(16)$	*[(NH3+)**N2**-Hn4···Cl1(Cl-)]$_2$ & (CH2)C4-H4a···F1
		*[(NH3+)**N2**-Hn4···Cl1(Cl-)]$_2$ & (CH)C4-H4b···F2
		*[(NH3+)**N2**-Hn5···Ow(H2O)]$_2$ & (CH2)C4-H4a···F1
		*(NH3+)**N2**-Hn5···Ow(H2O) & (CH)C4-H4b···F2
		*[(NH3+)**N2**-Hn6···Cl1(Cl-)]$_2$ & (CH2)C4-H4a···F1
		*[(NH3+)**N2**-Hn6···Cl1(Cl-)]$_2$ & (CH)C4-H4b···F2
	$D^3_3(18)$	*(NH3+)**N1**-Hn2···**O2(COO-)** & [(NH3+)**N1**-Hn3···Ow(H2O)]$_2$
		*(NH3+)**N1**-Hn2···**O2(COO-)** & (NH3+)**N2**-Hn5···Ow(H2O)
		*(NH3+)**N1**-Hn2···**O2(COO-)** & [(NH3+)**N2**-Hn4···Cl1(Cl-)]$_2$
		*(NH3+)**N1**-Hn2···**O2(COO-)** & [(NH3+)**N2**-Hn6···Cl1(Cl-)]$_2$
		*[(NH3+)**N2**-Hn5···Ow(H2O)]$_2$ & (CH)C3-H3···**O1(COO-)**
		*[(NH3+)**N2**-Hn4···Cl1(Cl-)]$_2$ & (CH)C3-H3···**O1(COO-)**
		*[(NH3+)**N2**-Hn6···Cl1(Cl-)]$_2$ & (CH)C3-H3···**O1(COO-)**
**2**		
	C(5)	*(NH3+)N1-H1···O1(COO-)
		*(NH3+)N1-H3···O1(COO-)
		*(CH2)C3-H6···O1(COO-)
	C(6)	*(CH2)C4-H8···O2(COO-)
	C(7)	(CH2)C5-H10···O2(COO-)
	C(8)	*(NH3+)N2-H13···O2(COO-)
	D(2)	*(NH3+)N1-H2···Cl1

		*$_{(NH3+)}$N2-H11···Cl1
		*$_{(NH3+)}$N2-H12···Cl1
		*$_{(CH2)}$C3-H5···Cl1
		*$_{(CH2)}$C4-H7···Cl1
		*$_{(CH2)}$C5-H9···Cl1
Level 2		
	$C^1_2(4)$	*$_{(NH3+)}$N1-H1···O1$_{(COO-)}$ & $_{(NH3+)}$N1-H3···O1$_{(COO-)}$
		*$_{(NH3+)}$N2-H11···Cl1 & $_{(NH3+)}$N2-H12···Cl1
	$C^1_2(5)$	*$_{(NH3+)}$N2-H11···Cl1 & $_{(CH2)}$C5-H9···Cl1
		*$_{(NH3+)}$N2-H12···Cl1 & $_{(CH2)}$C5-H9···Cl1
		*$_{(NH3+)}$N2-H13···O2$_{(COO-)}$ & $_{(CH2)}$C5-H10···O2$_{(COO-)}$
		*[$_{(CH2)}$C3-H5···Cl1]$_2$ & $_{(CH2)}$C4-H7···Cl1
		*[$_{(CH2)}$C4-H7···Cl1]$_2$ & $_{(CH2)}$C5-H9···Cl1
		*$_{(CH2)}$C4-H8···O2$_{(COO-)}$ & $_{(CH2)}$C5-H10···O2$_{(COO-)}$
	$C^1_2(6)$	*$_{(NH3+)}$N1-H1···O1$_{(COO-)}$ & $_{(CH2)}$C3-H6···O1$_{(COO-)}$
		*$_{(NH3+)}$N2-H11···Cl1 & $_{(CH2)}$C4-H7···Cl1
		*$_{(CH2)}$C5-H9···Cl1 & $_{(CH2)}$C3-H5···Cl1
	$C^1_2(7)$	*$_{(NH3+)}$N1-H2···Cl1 & $_{(CH2)}$C4-H7···Cl1
		*$_{(NH3+)}$N2-H11···Cl1 & $_{(CH2)}$C3-H5···Cl1
		*$_{(NH3+)}$N2-H12···Cl1 & $_{(CH2)}$C3-H5···Cl1
	$C^1_2(8)$	$_{(NH3+)}$N1-H2···Cl1 & $_{(CH2)}$C5-H9···Cl1
	$C^1_2(9)$	*$_{(NH3+)}$N1-H2···Cl1 & $_{(NH3+)}$N2-H11···Cl1
		*[$_{(NH3+)}$N1-H2···Cl1]$_2$ & $_{(NH3+)}$N2-H12···Cl1
	$C^2_2(7)$	$_{(CH2)}$C4-H8···O2$_{(COO-)}$ & $_{(CH2)}$C3-H6···O1$_{(COO-)}$
		$_{(CH2)}$C5-H10···O2$_{(COO-)}$ & $_{(CH2)}$C3-H6···O1$_{(COO-)}$
	$C^2_2(9)$	*$_{(NH3+)}$N1-H1···O1$_{(COO-)}$ & $_{(CH2)}$C4-H8···O2$_{(COO-)}$
		*$_{(NH3+)}$N1-H3···O1$_{(COO-)}$ & $_{(CH2)}$C4-H8···O2$_{(COO-)}$
		*$_{(NH3+)}$N2-H13···O2$_{(COO-)}$ & $_{(CH2)}$C3-H6···O1$_{(COO-)}$
	$C^2_2(10)$	*$_{(NH3+)}$N1-H1···O1$_{(COO-)}$ & $_{(NH3+)}$N1-H3···O1$_{(COO-)}$
		*$_{(NH3+)}$N1-H1···O1$_{(COO-)}$ & $_{(CH2)}$C3-H6···O1$_{(COO-)}$
		*$_{(NH3+)}$N1-H1···O1$_{(COO-)}$ & $_{(CH2)}$C5-H10···O2$_{(COO-)}$
		*$_{(NH3+)}$N1-H3···O1$_{(COO-)}$ & $_{(CH2)}$C3-H6···O1$_{(COO-)}$
		*$_{(NH3+)}$N1-H3···O1$_{(COO-)}$ & $_{(CH2)}$C5-H10···O2$_{(COO-)}$
	$C^2_2(11)$	*$_{(NH3+)}$N1-H1···O1$_{(COO-)}$ & [$_{(NH3+)}$N2-H13···O2$_{(COO-)}$]$_2$
		*$_{(NH3+)}$N1-H3···O1$_{(COO-)}$ & $_{(NH3+)}$N2-H13···O2$_{(COO-)}$
		*$_{(NH3+)}$N1-H3···O1$_{(COO-)}$ & $_{(CH2)}$C4-H8···O2$_{(COO-)}$
		*$_{(CH2)}$C4-H8···O2$_{(COO-)}$ & $_{(CH2)}$C3-H6···O1$_{(COO-)}$
	$C^2_2(12)$	*$_{(NH3+)}$N1-H1···O1$_{(COO-)}$ & $_{(CH2)}$C5-H10···O2$_{(COO-)}$
		*$_{(NH3+)}$N1-H3···O1$_{(COO-)}$ & $_{(CH2)}$C5-H10···O2$_{(COO-)}$
		*$_{(CH2)}$C5-H10···O2$_{(COO-)}$ & $_{(CH2)}$C3-H6···O1$_{(COO-)}$
	$C^2_2(13)$	*$_{(NH3+)}$N1-H3···O1$_{(COO-)}$ & $_{(NH3+)}$N2-H13···O2$_{(COO-)}$
		*$_{(NH3+)}$N2-H13···O2$_{(COO-)}$ & $_{(CH2)}$C3-H6···O1$_{(COO-)}$
		*$_{(CH2)}$C4-H8···O2$_{(COO-)}$ & $_{(CH2)}$C5-H10···O2$_{(COO-)}$
	$C^2_2(14)$	*$_{(NH3+)}$N2-H13···O2$_{(COO-)}$ & $_{(CH2)}$C4-H8···O2$_{(COO-)}$
	$C^2_2(15)$	*$_{(NH3+)}$N2-H13···O2$_{(COO-)}$ & $_{(CH2)}$C5-H10···O2$_{(COO-)}$
	$C^3_4(14)$	[$_{(NH3+)}$N1-H1···O1$_{(COO-)}$]$_2$ & [$_{(NH3+)}$N1-H3···O1$_{(COO-)}$]$_2$
	$C^3_4(16)$	[$_{(NH3+)}$N1-H1···O1$_{(COO-)}$]$_2$ & [$_{(CH2)}$C3-H6···O1$_{(COO-)}$]$_2$
	$C^3_4(18)$	[$_{(CH2)}$C4-H8···O2$_{(COO-)}$]$_2$ & [$_{(CH2)}$C5-H10···O2$_{(COO-)}$]$_2$
	$C^3_4(20)$	[$_{(NH3+)}$N2-H13···O2$_{(COO-)}$]$_2$ & [$_{(CH2)}$C5-H10···O2$_{(COO-)}$]$_2$
	$C^4_4(18)$	[$_{(CH2)}$C4-H8···O2$_{(COO-)}$]$_2$ & [$_{(CH2)}$C3-H6···O1$_{(COO-)}$]$_2$
	$C^4_4(20)$	[$_{(NH3+)}$N1-H3···O1$_{(COO-)}$]$_2$ & [$_{(CH2)}$C4-H8···O2$_{(COO-)}$]$_2$
		[$_{(CH2)}$C5-H10···O2$_{(COO-)}$]$_2$ & [$_{(CH2)}$C3-H6···O1$_{(COO-)}$]$_2$
	$R^1_2(6)$	*$_{(NH3+)}$N1-H2···Cl1 & $_{(CH2)}$C3-H5···Cl1
		*$_{(NH3+)}$N1-H3···O1$_{(COO-)}$ & $_{(CH2)}$C3-H6···O1$_{(COO-)}$
		*[$_{(NH3+)}$N2-H12···Cl1]$_2$ & $_{(CH2)}$C4-H7···Cl1
		*$_{(NH3+)}$N2-H13···O2$_{(COO-)}$ & $_{(CH2)}$C4-H8···O2$_{(COO-)}$
	$R^2_2(11)$	$_{(NH3+)}$N1-H1···O1$_{(COO-)}$ & $_{(CH2)}$C4-H8···O2$_{(COO-)}$
	$R^2_2(13)$	$_{(NH3+)}$N1-H1···O1$_{(COO-)}$ & $_{(NH3+)}$N2-H13···O2$_{(COO-)}$
	$R^3_4(14)$	[$_{(NH3+)}$N1-H1···O1$_{(COO-)}$]$_2$ & [$_{(NH3+)}$N1-H3···O1$_{(COO-)}$]$_2$
	$R^3_4(16)$	[$_{(NH3+)}$N1-H1···O1$_{(COO-)}$]$_2$ & [$_{(CH2)}$C3-H6···O1$_{(COO-)}$]$_2$
	$R^3_4(18)$	[$_{(CH2)}$C4-H8···O2$_{(COO-)}$]$_2$ & [$_{(CH2)}$C5-H10···O2$_{(COO-)}$]$_2$
	$R^3_4(20)$	[$_{(NH3+)}$N2-H13···O2$_{(COO-)}$]$_2$ & [$_{(CH2)}$C5-H10···O2$_{(COO-)}$]$_2$
	$R^4_4(18)$	[$_{(CH2)}$C4-H8···O2$_{(COO-)}$]$_2$ & [$_{(CH2)}$C3-H6···O1$_{(COO-)}$]$_2$
	$R^4_4(20)$	[$_{(NH3+)}$N1-H3···O1$_{(COO-)}$]$_2$ & [$_{(CH2)}$C4-H8···O2$_{(COO-)}$]$_2$
		[$_{(CH2)}$C5-H10···O2$_{(COO-)}$]$_2$ & [$_{(CH2)}$C3-H6···O1$_{(COO-)}$]$_2$
	$D^3_3(10)$	*$_{(NH3+)}$N1-H1···O1$_{(COO-)}$ & [$_{(NH3+)}$N1-H2···Cl1]$_2$
		*$_{(NH3+)}$N1-H3···O1$_{(COO-)}$ & [$_{(NH3+)}$N1-H2···Cl1]$_2$

	*$_{(CH2)}$C3-H6···O1$_{(COO-)}$ & [$_{(CH2)}$C3-H5···Cl1]$_2$
D^3_3(11)	*$_{(CH2)}$C4-H8···O2$_{(COO-)}$ & [$_{CH2)}$C3-H5···Cl1]$_2$
	*[$_{(CH2)}$C4-H7···Cl1]$_2$ & $_{(CH2)}$C4-H8···O2$_{(COO-)}$
D^3_3(12)	*$_{(NH3+)}$N1-H1···O1$_{(COO-)}$ & [$_{(CH2)}$C3-H5···Cl1]$_2$
	*$_{(NH3+)}$N1-H2···Cl1 & $_{(CH2)}$C3-H6···O1$_{(COO-)}$
	*$_{(NH3+)}$N1-H3···O1$_{(COO-)}$ & [$_{(CH2)}$C3-H5···Cl1]$_2$
	*$_{(CH2)}$C5-H10···O2$_{(COO-)}$ & [$_{(CH2)}$C3-H5···Cl1]$_2$
	*[$_{(CH2)}$C4-H7···Cl1]$_2$ & $_{(CH2)}$C3-H6···O1$_{(COO-)}$
	*[$_{(CH2)}$C4-H7···Cl1]$_2$ & $_{(CH2)}$C5-H10···O2$_{(COO-)}$
	*$_{(CH2)}$C5-H10···O2$_{(COO-)}$ & [$_{(CH2)}$C5-H9···Cl1]$_2$
D^3_3(13)	*$_{(NH3+)}$N1-H2···Cl1 & [$_{(CH2)}$C4-H8···O2$_{(COO-)}$]$_2$
	*[$_{(NH3+)}$N2-H11···Cl1]$_2$ & $_{(NH3+)}$N2-H13···O2$_{(COO-)}$
	*$_{(NH3+)}$N2-H13···O2$_{(COO-)}$ & [$_{(NH3+)}$N2-H12···Cl1]$_2$
	*$_{(NH3+)}$N2-H13···O2$_{(COO-)}$ & [$_{CH2)}$C3-H5···Cl1]$_2$
	*$_{(NH3+)}$N2-H13···O2$_{(COO-)}$ & [$_{(CH2)}$C4-H7···Cl1]$_2$
	*$_{(NH3+)}$N2-H13···O2$_{(COO-)}$ & [$_{(CH2)}$C5-H9···Cl1]$_2$
	*$_{(CH2)}$C4-H8···O2$_{(COO-)}$ & [$_{(CH2)}$C5-H9···Cl1]$_2$
D^3_3(14)	*$_{(NH3+)}$N1-H1···O1$_{(COO-)}$ & [$_{(CH2)}$C4-H7···Cl1]$_2$
	*[$_{(NH3+)}$N1-H2···Cl1]$_2$ & $_{(CH2)}$C5-H10···O2$_{(COO-)}$
	*$_{(NH3+)}$N1-H3···O1$_{(COO-)}$ & [$_{(CH2)}$C4-H7···Cl1]$_2$
	*[$_{(NH3+)}$N2-H11···Cl1]$_2$ & $_{(CH2)}$C5-H10···O2$_{(COO-)}$
	*[$_{(NH3+)}$N2-H12···Cl1]$_2$ & $_{(CH2)}$C5-H10···O2$_{(COO-)}$
	*$_{(CH2)}$C3-H6···O1$_{(COO-)}$ & [$_{(CH2)}$C5-H9···Cl1]$_2$
D^3_3(15)	*$_{(NH3+)}$N1-H2···Cl1 & $_{(NH3+)}$N2-H13···O2$_{(COO-)}$
	*[$_{(NH3+)}$N2-H11···Cl1]$_2$ & $_{(CH2)}$C4-H8···O2$_{(COO-)}$
	*[$_{(NH3+)}$N2-H12···Cl1]$_2$ & $_{(CH2)}$C4-H8···O2$_{(COO-)}$
D^3_3(16)	*$_{(NH3+)}$N1-H1···O1$_{(COO-)}$ & [$_{(CH2)}$C5-H9···Cl1]$_2$
	*$_{(NH3+)}$N1-H3···O1$_{(COO-)}$ & [$_{(CH2)}$C5-H9···Cl1]$_2$
	*[$_{(NH3+)}$N2-H11···Cl1]$_2$ & $_{(CH2)}$C3-H6···O1$_{(COO-)}$
	*[$_{(NH3+)}$N2-H12···Cl1]$_2$ & $_{(CH2)}$C3-H6···O1$_{(COO-)}$
D^3_3(18)	*$_{(NH3+)}$N1-H1···O1$_{(COO-)}$ & [$_{(NH3+)}$N2-H11···Cl1]$_2$
	*$_{(NH3+)}$N1-H1···O1$_{(COO-)}$ & [$_{(NH3+)}$N2-H12···Cl1]$_2$
	*$_{(NH3+)}$N1-H3···O1$_{(COO-)}$ & [$_{(NH3+)}$N2-H11···Cl1]$_2$
	*$_{(NH3+)}$N1-H3···O1$_{(COO-)}$ & [$_{(NH3+)}$N2-H12···Cl1]$_2$

TABLE S22 Supramolecular synthons involving fluorine in **1**.

Synthon	***level 1***	***level 2***
$_{(CH2)}$C-H···F	C(5), C(7)	R^1_2(6), C^2_2(12), C^4_4(16), C^4_4(20)
$_{(CH2/CH)}$C-H···F & $_{(H2O)}$**O-H···O**$_{(COO-)}$		D^3_3(12), D^3_3(14)
$_{(CH2)}$C-H···F & $_{(NH3+)}$**N-H···O**$_{(COO-)}$		C^2_2(10), C^2_2(12), C^4_4(20), R^4_4(20)
$_{(CH2)}$C-H···F & $_{(NH3+)}$N-H···O$_{(H2O)}$		D^3_3(12), D^3_3(14), D^3_3(16)
$_{(CH2)}$C-H···F & $_{(NH3+)}$**N-H···Cl$^-$**$_{(Cl-)}$		D^3_3(12), D^3_3(14), D^3_3(16)
$_{(CH2/CH)}$C-H···F & $_{(CH)}$**C-H···O**$_{(COO-)}$		C^2_2(8), C^2_2(10), C^4_4(18), R^2_2(10), R^2_2(12), R^4_4(18)
$_{(CH2)}$C-H···F & $_{(CH)}$C-H···O$_{(H2O)}$		D^3_3(10), D^3_3(12)
$_{(CH2)}$C-H···F & $_{(CH)}$C-H···F homosynthon		C^2_2(6), C^2_2(8), C^2_2(10), R^4_4(16), R^4_4(20)
$_{(CH2/CH)}$C-H···F & $_{(CH2)}$**C-H···Cl$^-$**$_{(Cl-)}$		D^3_3(12)

TABLE S23 SwissADME evaluation of ornithine derivatives.

	Formula<br><br>*Compound name*	*Bioavailability radar*	*BOILED egg*	*Lipophilicity iLOGP*	*Hydrophilicity ESOL*	*BBB Permeability*

1	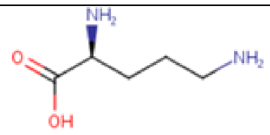 *L*-ornithine	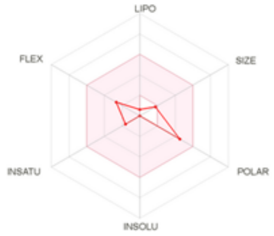	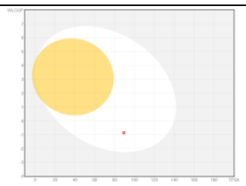	0.49	2.38	No
2	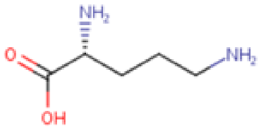 *D*-ornithine	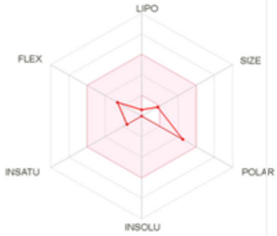	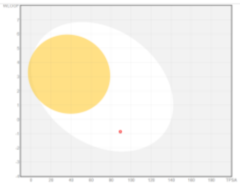	0.62	2.38	No
3	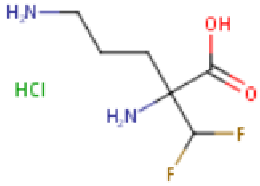 *D*,*L*-DFMO hydrochloride	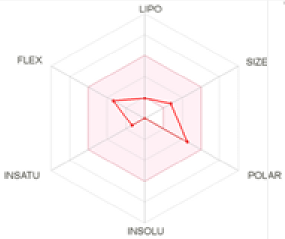	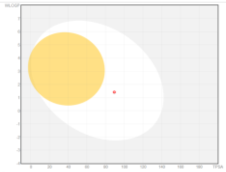	0	0.47	No
4	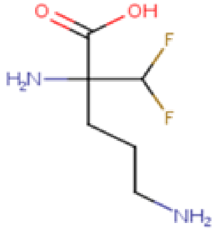 *D*,*L*-DFMO uncharged	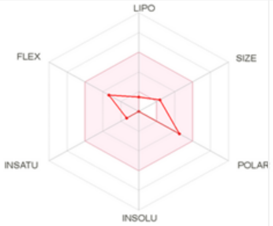	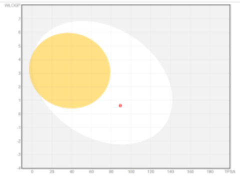	0.53	1.2	No
5	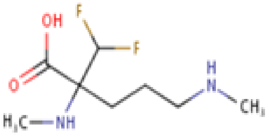 *D*,*L*-DFMO alpha delta NMe	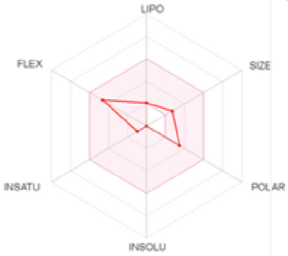	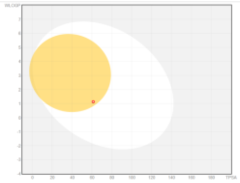	1.48	0.51	Yes
6		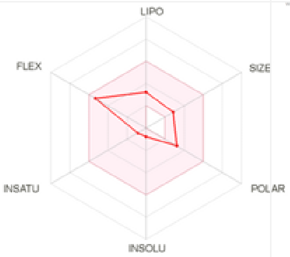	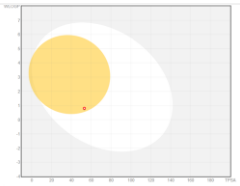	1.95	-0.78	Yes

	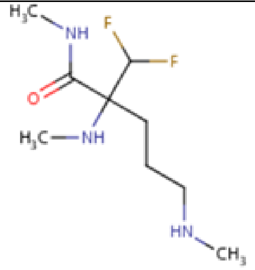 *D,L*-DFMO Me-amide alpha delta NMe					
7	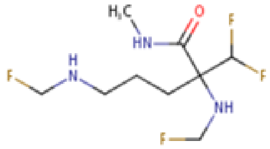 *D,L*-DFMO Me-amide alpha delta NMeF	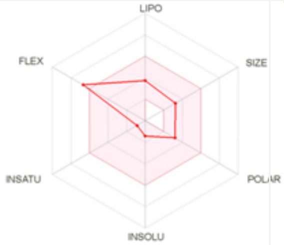	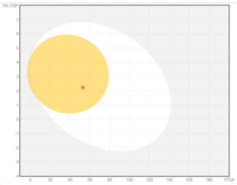	1.42	-1.43	Yes
8	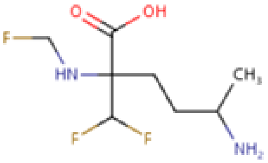 *D,L*-DFMO alpha NMeF delta Me	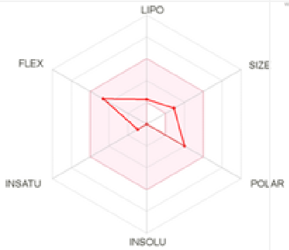	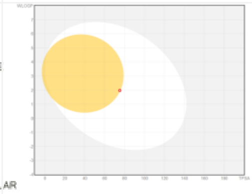	1.27	0.17	Yes
9	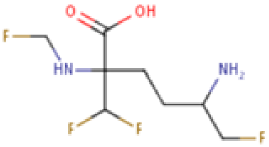 *D,L*-DFMO alpha NMe delta MeF	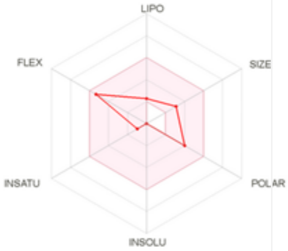	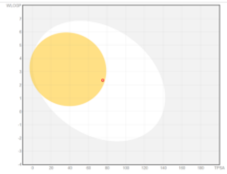	1.2	0.14	Yes
